# Supplementary material for: The Evolution and Characterization of the RNA Interference Pathways in Lophotrochozoa
Source: Genome Biol Evol. 2024 May 7;16(5):evae098. doi: 10.1093/gbe/evae098 (PMC11114477; doi:10.1093/gbe/evae098)
Supplement: evae098_Supplementary_Data [file evae098_supplementary_data.zip › Supplementary_materials.zip!/selected_miRNAs/NoGe_selected_miRNAs/mirdeep_pdf_files/NMRB01001187.1_Notospermus_geniculatus_strain_Ushimado_scaffold1187,_whole_genome_shotgun_sequence_26013.pdf]

Provisional ID : NMRB01001187.1_Notospermus_geniculatus_strain_Ushimado_scaffold1187,_whole_genome_shotgun_sequence_26013
Score total : 2368407.4
Score for star read(s) : 3.9
Score for read counts : 2368398.9
Score for mfe : 1.7
Score for randfold : 0
Score for cons. seed : 3
Total read count : 4645524
Mature read count : 4645442
Loop read count : 0
Star read count : 82

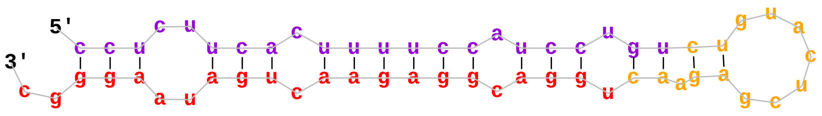

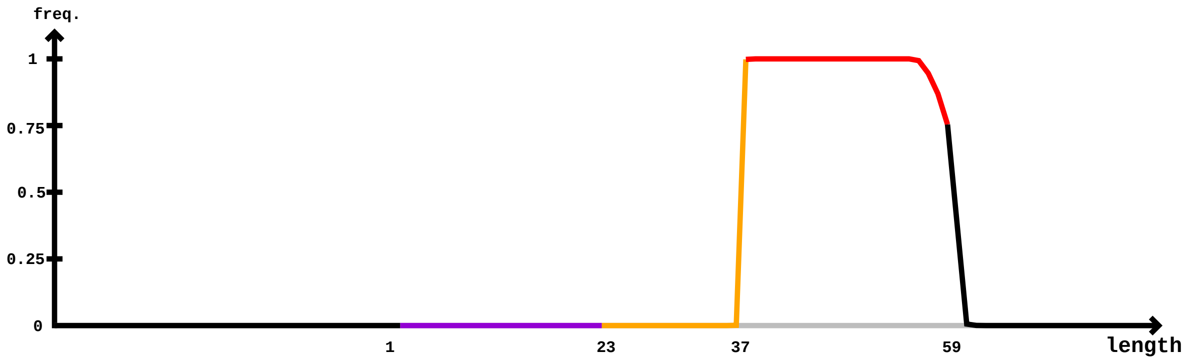


```
                                    Star                                  Mature
5'- cauuugugacaucagaaguaagucuguuucguguccucuucacuuuuccauccugucuguacucgagaacuggacggagaacugauaagggcauagggcagaccacaucaaca   -3'   obs
    cauuugugacaucagaaguaagucuguuucguguccucuucacuuuuccauccugucuguacucgagaacuggacggagaacugauaagggcauagggcagaccacaucaaca         exp
    .((((.((....)).))))..((((((((.(((((((..(((.((((((.(((.((((.......)).)).))).)))))).)))..))))))).))))))))..........   reads   mm   sample
    ..................................ccucuucacuuuuccauccugu.........................................................   5       0    Fm3
    ..................................ccucuucacuuuuUcauccugu.........................................................   2       1    Fm3
    ...................................cucuucacuuuuccauccugu.........................................................   1       0    Fm3
    ...................................................................aacuggacggagaacugauaaU........................   1       1    Fm3
    ...................................................................aacuggacggagaacugauaagU.......................   1       1    Fm3
    ...................................................................aUcuggacggagaacugauaagggc.....................   4       1    Fm3
    ....................................................................acuggacggagaacugaua..........................   2       0    Fm3
    ....................................................................acuggacggagaacugauaag........................   3       0    Fm3
    ....................................................................acuggacggagaacugauaagg.......................   6       0    Fm3
    ....................................................................acuggacggagaacugauaagU.......................   6       1    Fm3
    ....................................................................Ucuggacggagaacugauaagg.......................   1       1    Fm3
    ....................................................................Ucuggacggagaacugauaaggg......................   2       1    Fm3
    ....................................................................acuggacggagaacugauaaggA......................   4       1    Fm3
    ....................................................................acuggacggagaacugauaaggU......................   7       1    Fm3
    ....................................................................Ucuggacggagaacugauaagggc.....................   1       1    Fm3
    ....................................................................acuggacggagaacugauaagggc.....................   1       0    Fm3
    .....................................................................cuggacggagaacugauU..........................   1       1    Fm3
    .....................................................................cuggacggagaacugaua..........................   12      0    Fm3
    .....................................................................cuggacggagaacugaAa..........................   5       1    Fm3
    .....................................................................cuggacggagaacugauaG.........................   1       1    Fm3
    .....................................................................cuggacggagaacugauaa.........................   16      0    Fm3
    .....................................................................cuggacggagaacugauaaU........................   11      1    Fm3
    .....................................................................Guggacggagaacugauaag........................   1       1    Fm3
    .....................................................................cuggacggagaacugauUag........................   1       1    Fm3
    .....................................................................cuggacggagaacugauaag........................   103     0    Fm3
    .....................................................................cuggacggagaacugauaaA........................   23      1    Fm3
    .....................................................................cuggacggagaacugauGagg.......................   1       1    Fm3
    .....................................................................cuggacggagaacugauCagg.......................   2       1    Fm3
    .....................................................................cuggacggagaacugaAaagg.......................   1       1    Fm3
    .....................................................................cuggacggagGacugauaagg.......................   1       1    Fm3
    .....................................................................cuggacggagaacugaCaagg.......................   3       1    Fm3
    .....................................................................cuggacggagCacugauaagg.......................   1       1    Fm3
    .....................................................................cuggacggagaacugauaaAg.......................   1       1    Fm3
```

Star Mature

cauuugugacaucagaaguaagucuguuucguguccucuucacuuuuccauccugucuguacucgagaacuggacggagaacugauaagggcauagggcagaccacaucaaca			
....cuggacggagaacugauUagg.......................	1	1	Fm3
....cuggacggagaacugauaagU.......................	9	1	Fm3
....cuggacggagaacugauaagA.......................	70	1	Fm3
....cuggacggagaacugauaagg.......................	238	0	Fm3
....cuggacggagCacugauaaggg......................	2	1	Fm3
....cuggacggagaacugauaaggg......................	284	0	Fm3
....cuggacggagaacugauaaggC......................	3	1	Fm3
....Nuggacggagaacugauaaggg......................	1	1	Fm3
....cuggacggagaacugauCaggg......................	1	1	Fm3
....cuggacggagaacugaGaaggg......................	1	1	Fm3
....cuggacggagaaAugauaaggg......................	1	1	Fm3
....cuggacggagaacugauaaggA......................	67	1	Fm3
....cuggacggagGacugauaaggg......................	2	1	Fm3
....cuggacggagaacugauGaggg......................	3	1	Fm3
....cuggacggagaacugauaaggU......................	46	1	Fm3
....cuggacggagaacugaAaagggc.....................	1	1	Fm3
....cuggUcggagaacugauaagggc.....................	1	1	Fm3
....cuggacggagaacugauaagggU.....................	26	1	Fm3
....cuggacggagaacugauaagggA.....................	64	1	Fm3
....cuggacggagaacugauaagggc.....................	212	0	Fm3
....cuggacggagaacugauaGgggc.....................	2	1	Fm3
....cuggacggGgaacugauaagggc.....................	1	1	Fm3
....cuggacggagaacugaCaagggc.....................	1	1	Fm3
....cuggacggagaacugauCagggca....................	1	1	Fm3
....cuggacggagaacugauaagggca....................	51	0	Fm3
....cuggacggagaacugauaUgggca....................	1	1	Fm3
....cuggacggagaacugauaagggAa....................	1	1	Fm3
....cuggacggagaaUugauaagggca....................	1	1	Fm3
....cuggacggagaUcugauaagggca....................	3	1	Fm3
....uggacggagaacuCauaa.........................	1	1	Fm3
....uggacggagaacugauGa.........................	23	1	Fm3
....uggacggagaacugaAaa.........................	1643	1	Fm3
....uggacggagaacugauUa.........................	54	1	Fm3
....Nggacggagaacugauaa.........................	6	1	Fm3
....uggacggagaacugUuaa.........................	5	1	Fm3
....uggGcggagaacugauaa.........................	2	1	Fm3
....uggacggCgaacugauaa.........................	1	1	Fm3
....uggacggagaacGgauaa.........................	1	1	Fm3
....ugUacggagaacugauaa.........................	9	1	Fm3
....uggacggagaacCgauaa.........................	3	1	Fm3
....uggacggaCaacugauaa.........................	2	1	Fm3
....Cggacggagaacugauaa.........................	1	1	Fm3
....uggacggagCacugauaa.........................	2	1	Fm3
....uggacggagGacugauaa.........................	15	1	Fm3
....uggacggagaaUugauaa.........................	1	1	Fm3
....uggacgNagaacugauaa.........................	1	1	Fm3
....uggCcggagaacugauaa.........................	6	1	Fm3
....uggacgUagaacugauaa.........................	7	1	Fm3
....uggacgAagaacugauaa.........................	3	1	Fm3
....uggacggagaacugauaa.........................	18785	0	Fm3
....uggacggagaacugauaU.........................	591	1	Fm3
....uCgacggagaacugauaa.........................	3	1	Fm3
....uAgacggagaacugauaa.........................	14	1	Fm3
....uggacggaUaacugauaa.........................	3	1	Fm3
....uggacggagaacugauaC.........................	58	1	Fm3
....uggacAgagaacugauaa.........................	2	1	Fm3
....uggacggagaGcugauaa.........................	27	1	Fm3
....uggaGggagaacugauaa.........................	1	1	Fm3
....uggacggUgaacugauaa.........................	7	1	Fm3
....ugCacggagaacugauaa.........................	1	1	Fm3
....uggacggagaacuAauaa.........................	6	1	Fm3
....uUgacggagaacugauaa.........................	4	1	Fm3
....uggacggagaacugauCa.........................	41	1	Fm3
....uggaUggagaacugauaa.........................	6	1	Fm3
....uggUcggagaacugauaa.........................	1	1	Fm3
....uggacggaAaacugauaa.........................	2	1	Fm3
....uggacggagaCcugauaa.........................	13	1	Fm3
....Aggacggagaacugauaa.........................	2	1	Fm3
....uggacgCagaacugauaa.........................	1	1	Fm3
....uggacggagaacugaGaa.........................	5	1	Fm3

Star Mature

cauuugugacaucagaaguaagucuguuucguguccucuucacuuuuccauccuguCuguacucgagaacuggacggagaacugauaagggcauagggcagaccacaucaaca			
....ugAacggagaacugauaa.........................	5	1	Fm3
....uggacggagaacugGuaa.........................	1	1	Fm3
....uggacggagaacugauaG.........................	105	1	Fm3
....uggacUgagaacugauaa.........................	8	1	Fm3
....uggacggGgaacugauaa.........................	3	1	Fm3
....uggUcggagaacugauaag........................	14	1	Fm3
....Gggacggagaacugauaag........................	4	1	Fm3
....uggacggagaacAgauaag........................	7	1	Fm3
....uggacggagaacugauaCg........................	298	1	Fm3
....uggacggagaacugauaaC........................	52	1	Fm3
....uggacNgagaacugauaag........................	7	1	Fm3
....uggacggagaacGgauaag........................	1	1	Fm3
....uggacggagaacugUuaag........................	10	1	Fm3
....uAgacggagaacugauaag........................	53	1	Fm3
....uggacCgagaacugauaag........................	2	1	Fm3
....uCgacggagaacugauaag........................	6	1	Fm3
....uggacggagaacugGuaag........................	9	1	Fm3
....ugAacggagaacugauaag........................	17	1	Fm3
....uggacggagaacugauaaA........................	48211	1	Fm3
....ugNacggagaacugauaag........................	1	1	Fm3
....uggaAggagaacugauaag........................	11	1	Fm3
....uggacggagaacugCuaag........................	4	1	Fm3
....uggacUgagaacugauaag........................	28	1	Fm3
....uggacggagaacugauGag........................	102	1	Fm3
....uggGcggagaacugauaag........................	17	1	Fm3
....uggacggagaacugaGaag........................	2	1	Fm3
....uggacggagaacugauaag........................	75493	0	Fm3
....uggacggCgaacugauaag........................	6	1	Fm3
....uggacggagaacuCauaag........................	7	1	Fm3
....uggacggagaaGugauaag........................	3	1	Fm3
....uggacggaAaacugauaag........................	4	1	Fm3
....ugCacggagaacugauaag........................	8	1	Fm3
....uggacggagaCcugauaag........................	116	1	Fm3
....ugUacggagaacugauaag........................	27	1	Fm3
....Cggacggagaacugauaag........................	9	1	Fm3
....uggaGggagaacugauaag........................	7	1	Fm3
....uggacggaCaacugauaag........................	2	1	Fm3
....uggacggagaacCgauaag........................	9	1	Fm3
....uggacggagaacugaAaag........................	60	1	Fm3
....uggaUggagaacugauaag........................	21	1	Fm3
....uggacgNagaacugauaag........................	3	1	Fm3
....uggacgUagaacugauaag........................	35	1	Fm3
....uUgacggagaacugauaag........................	37	1	Fm3
....uggacggagaaUugauaag........................	23	1	Fm3
....uggacggGgaacugauaag........................	9	1	Fm3
....uggacggagaacugauUag........................	116	1	Fm3
....uggacggagaacugauaaU........................	15180	1	Fm3
....uggacAgagaacugauaag........................	17	1	Fm3
....uggacggagaacuUauaag........................	8	1	Fm3
....uggacggaUaacugauaag........................	19	1	Fm3
....Aggacggagaacugauaag........................	41	1	Fm3
....uggCcggagaacugauaag........................	11	1	Fm3
....uggacggagaacugauaUg........................	256	1	Fm3
....uggacggagaacugaCaag........................	8	1	Fm3
....uggacggagaacugauCag........................	167	1	Fm3
....uggacggagCacugauaag........................	5	1	Fm3
....uggacggUgaacugauaag........................	17	1	Fm3
....uggacggagaacugauaGg........................	245	1	Fm3
....uggacgAagaacugauaag........................	6	1	Fm3
....uggacgCagaacugauaag........................	7	1	Fm3
....uggacggagaaAugauaag........................	2	1	Fm3
....uggacggagUacugauaag........................	18	1	Fm3
....uggacggagGacugauaag........................	72	1	Fm3
....uggacggagaGcugauaag........................	243	1	Fm3
....Nggacggagaacugauaag........................	30	1	Fm3
....uggacggagaacuAauaag........................	10	1	Fm3
....uNgacggagaacugauaag........................	2	1	Fm3
....uggUcggagaacugauaagg.......................	25	1	Fm3
....uggacggagaacugauCagg.......................	354	1	Fm3
....uggacggagaacugauaCgg.......................	648	1	Fm3

Star Mature

cauuugugacaucagaaguaagucuguuucguguccucuucacuuuuccauccugucuguacucgagaacuggacggagaacugauaagggcauagggcagaccacaucaaca			
....uggacggagGacugauaagg.......................	235	1	Fm3
....uggacggagaacugauaagU.......................	5388	1	Fm3
....Nggacggagaacugauaagg.......................	83	1	Fm3
....uggacggaCaacugauaagg.......................	11	1	Fm3
....Aggacggagaacugauaagg.......................	638	1	Fm3
....uggacggagaacugaAaagg.......................	7	1	Fm3
....uggacggagaaGugauaagg.......................	4	1	Fm3
....uggacggagaacugauaaAg.......................	936	1	Fm3
....uggacggagCacugauaagg.......................	14	1	Fm3
....uggaGggagaacugauaagg.......................	8	1	Fm3
....uggacNgagaacugauaagg.......................	17	1	Fm3
....uggacggagaCcugauaagg.......................	1074	1	Fm3
....uggacggagaacugauaaCg.......................	5	1	Fm3
....uggacggagaacAgauaagg.......................	4	1	Fm3
....uggacggUgaacugauaagg.......................	37	1	Fm3
....uggacggagaacGgauaagg.......................	7	1	Fm3
....uggacggaUaacugauaagg.......................	32	1	Fm3
....uggacgAagaacugauaagg.......................	12	1	Fm3
....uggaUggagaacugauaagg.......................	57	1	Fm3
....uggacggagaacugauaagC.......................	22	1	Fm3
....uggacggagaacCgauaagg.......................	19	1	Fm3
....uggacAgagaacugauaagg.......................	37	1	Fm3
....Gggacggagaacugauaagg.......................	16	1	Fm3
....uggacggagaacugauaGgg.......................	663	1	Fm3
....uggacggagaacugauUagg.......................	249	1	Fm3
....uCgacggagaacugauaagg.......................	18	1	Fm3
....uggacggagaacugauaagg.......................	154018	0	Fm3
....uggacggaAaacugauaagg.......................	10	1	Fm3
....uggacggagaacuCauaagg.......................	9	1	Fm3
....ugCacggagaacugauaagg.......................	13	1	Fm3
....uggacggCgaacugauaagg.......................	9	1	Fm3
....uggacggGgaacugauaagg.......................	22	1	Fm3
....uggacgNagaacugauaagg.......................	9	1	Fm3
....uggacUgagaacugauaagg.......................	50	1	Fm3
....uggacgUagaacugauaagg.......................	46	1	Fm3
....uggacggagaacugaGaagg.......................	6	1	Fm3
....uggacCgagaacugauaagg.......................	6	1	Fm3
....uggacggagaacugauaUgg.......................	476	1	Fm3
....uggacggagaacuAauaagg.......................	33	1	Fm3
....uggacggagaacugUuaagg.......................	33	1	Fm3
....uggacggagaacugCuaagg.......................	10	1	Fm3
....uggacggagaGcugauaagg.......................	1812	1	Fm3
....uggCcggagaacugauaagg.......................	34	1	Fm3
....ugAacggagaacugauaagg.......................	36	1	Fm3
....uggacggagaacuUauaagg.......................	17	1	Fm3
....uNgacggagaacugauaagg.......................	1	1	Fm3
....uggacggagaacugauaagA.......................	43430	1	Fm3
....uUgacggagaacugauaagg.......................	57	1	Fm3
....uggacggagUacugauaagg.......................	32	1	Fm3
....uggacggagaaAugauaagg.......................	10	1	Fm3
....uggacggagaacugauaaUg.......................	45	1	Fm3
....Cggacggagaacugauaagg.......................	26	1	Fm3
....uggacggagaaUugauaagg.......................	31	1	Fm3
....uAgacggagaacugauaagg.......................	79	1	Fm3
....uggacggagaacugGuaagg.......................	15	1	Fm3
....uggacggagaacugaCaagg.......................	19	1	Fm3
....ugNacggagaacugauaagg.......................	1	1	Fm3
....ugUacggagaacugauaagg.......................	42	1	Fm3
....uggacgCagaacugauaagg.......................	11	1	Fm3
....uggacggagaacugauGagg.......................	266	1	Fm3
....uggaAggagaacugauaagg.......................	20	1	Fm3
....uggGcggagaacugauaagg.......................	36	1	Fm3
....ugAacggagaacugauaaggg......................	49	1	Fm3
....uggacggagaaUugauaaggg......................	50	1	Fm3
....uggacggagaacugaCaaggg......................	20	1	Fm3
....uggacggagaacugauaUggg......................	709	1	Fm3
....uggacggagaacugauCaggg......................	465	1	Fm3
....ugCacggagaacugauaaggg......................	14	1	Fm3
....uggacggagGacugauaaggg......................	195	1	Fm3
....uggGcggagaacugauaaggg......................	44	1	Fm3

Star Mature

```
cauuugugacaucagaaguaagucuguuucguguccucuucacuuuuccauccugucuguacucgagaacuggacggagaacugauaagggcauagggcagaccacaucaaca
......................................................................uggacggagaacugauaaCgg......................    5       1    Fm3
......................................................................Gggacggagaacugauaaggg......................    12      1    Fm3
......................................................................uggacggagaacugauaagAg......................    189     1    Fm3
......................................................................uggacgUagaacugauaaggg......................    63      1    Fm3
......................................................................uggacggagaacugauUaggg......................    327     1    Fm3
......................................................................uggCcggagaacugauaaggg......................    29      1    Fm3
......................................................................uggacggagaacugauaaggU......................    7452    1    Fm3
......................................................................uggaAggagaacugauaaggg......................    20      1    Fm3
......................................................................uNgacggagaacugauaaggg......................    3       1    Fm3
......................................................................uggacggaUaacugauaaggg......................    41      1    Fm3
......................................................................uggacggagaacugauaaUgg......................    103     1    Fm3
......................................................................uggacggUgaacugauaaggg......................    90      1    Fm3
......................................................................ugNacggagaacugauaaggg......................    1       1    Fm3
......................................................................Cggacggagaacugauaaggg......................    31      1    Fm3
......................................................................uggUcggagaacugauaaggg......................    33      1    Fm3
......................................................................uggacggagaacugauaGggg......................    1166    1    Fm3
......................................................................uggacggagaacugauGaggg......................    294     1    Fm3
......................................................................uggacggGgaacugauaaggg......................    44      1    Fm3
......................................................................uggaUggagaacugauaaggg......................    66      1    Fm3
......................................................................Aggacggagaacugauaaggg......................    389     1    Fm3
......................................................................uggacggagaacuCauaaggg......................    14      1    Fm3
......................................................................uggacggagaacugauaagUg......................    79      1    Fm3
......................................................................uggacggagaacugaAaaggg......................    13      1    Fm3
......................................................................uggacggagaCcugauaaggg......................    674     1    Fm3
......................................................................uggacggagaacugUuaaggg......................    48      1    Fm3
......................................................................uggacggagaacugGuaaggg......................    33      1    Fm3
......................................................................uggacggaCaacugauaaggg......................    8       1    Fm3
......................................................................uggacAgagaacugauaaggg......................    34      1    Fm3
......................................................................uggacggagaacugauaaggg......................    200595  0    Fm3
......................................................................ugUacggagaacugauaaggg......................    57      1    Fm3
......................................................................uggacgNagaacugauaaggg......................    10      1    Fm3
......................................................................uggacggCgaacugauaaggg......................    14      1    Fm3
......................................................................uUgacggagaacugauaaggg......................    58      1    Fm3
......................................................................uggacggagaacuUauaaggg......................    25      1    Fm3
......................................................................uggacgAagaacugauaaggg......................    13      1    Fm3
......................................................................uggacggagUacugauaaggg......................    41      1    Fm3
......................................................................uggacUgagaacugauaaggg......................    73      1    Fm3
......................................................................uggacggagaacAgauaaggg......................    10      1    Fm3
......................................................................uggacggagaacugCuaaggg......................    11      1    Fm3
......................................................................Nggacggagaacugauaaggg......................    111     1    Fm3
......................................................................uggacggagaacugauaaggC......................    78      1    Fm3
......................................................................uggacggagaacugauaaAgg......................    22      1    Fm3
......................................................................uggaGggagaacugauaaggg......................    5       1    Fm3
......................................................................uggacggagaGcugauaaggg......................    1292    1    Fm3
......................................................................uggacggagCacugauaaggg......................    17      1    Fm3
......................................................................uggacggagaacGgauaaggg......................    11      1    Fm3
......................................................................uggacgCagaacugauaaggg......................    12      1    Fm3
......................................................................uggacggaAaacugauaaggg......................    13      1    Fm3
......................................................................uggacggagaacugaGaaggg......................    4       1    Fm3
......................................................................uggacCgagaacugauaaggg......................    7       1    Fm3
......................................................................uggacNgagaacugauaaggg......................    30      1    Fm3
......................................................................uggacggagaacuAauaaggg......................    52      1    Fm3
......................................................................uAgacggagaacugauaaggg......................    98      1    Fm3
......................................................................uggacggagaaGugauaaggg......................    6       1    Fm3
......................................................................uggacggagaacugauaaggA......................    98094   1    Fm3
......................................................................uggacggagaacugauaCggg......................    780     1    Fm3
......................................................................uCgacggagaacugauaaggg......................    18      1    Fm3
......................................................................uggacggagaacCgauaaggg......................    22      1    Fm3
......................................................................uggacggagaacugauaagCg......................    15      1    Fm3
......................................................................uggacggagaaAugauaaggg......................    6       1    Fm3
......................................................................Nggacggagaacugauaagggc.....................    405     1    Fm3
......................................................................uggacggagaacugauaaNggc.....................    1       1    Fm3
......................................................................uggacNgagaacugauaagggc.....................    104     1    Fm3
......................................................................uggacgCagaacugauaagggc.....................    46      1    Fm3
......................................................................uggUcggagaacugauaagggc.....................    147     1    Fm3
......................................................................uggacggagaGcugauaagggc.....................    1460    1    Fm3
......................................................................uggacggagaacugauGagggc.....................    1829    1    Fm3
......................................................................uggacggagaacugauaaggAc.....................    157     1    Fm3
......................................................................uggacggCgaacugauaagggc.....................    42      1    Fm3
......................................................................uggacggaUaacugauaagggc.....................    182     1    Fm3
```

```
                                  Star                              Mature
cauuugugacaucagaaguaagucuguuucguguccucuucacuuuuccauccugucuguacucgagaacuggacggagaacugauaagggcauagggcagaccacaucaaca
......................................................................uggacggagaacugauaagUgc.....................	281	1	Fm3
......................................................................uggaGggagaacugauaagggc.....................	32	1	Fm3
......................................................................uggacggagaacugauaagAgc.....................	104	1	Fm3
......................................................................uggaUggagaacugauaagggc.....................	278	1	Fm3
......................................................................uggacggagUacugauaagggc.....................	123	1	Fm3
......................................................................uggacggagaacugauaaggCc.....................	71	1	Fm3
......................................................................uggacggagaacugauUagggc.....................	911	1	Fm3
......................................................................uggacggagaacugaGaagggc.....................	19	1	Fm3
......................................................................uggacggagaacugauCagggc.....................	1554	1	Fm3
......................................................................uggacggagaacugauaaCggc.....................	38	1	Fm3
......................................................................uggacggagaacAgauaagggc.....................	51	1	Fm3
......................................................................uggacggagaacugauaGgggc.....................	5830	1	Fm3
......................................................................uCgacggagaacugauaagggc.....................	85	1	Fm3
......................................................................uggacggagaacugauaaUggc.....................	308	1	Fm3
......................................................................uggacggagaacuAauaagggc.....................	167	1	Fm3
......................................................................uggacggagaacugauaagCgc.....................	49	1	Fm3
......................................................................uggGcggagaacugauaagggc.....................	198	1	Fm3
......................................................................ugNacggagaacugauaagggc.....................	1	1	Fm3
......................................................................Cggacggagaacugauaagggc.....................	141	1	Fm3
......................................................................uggacggaCaacugauaagggc.....................	45	1	Fm3
......................................................................ugCacggagaacugauaagggc.....................	61	1	Fm3
......................................................................uggacggagaacugauaagggA.....................	100006	1	Fm3
......................................................................uggacggagCacugauaagggc.....................	55	1	Fm3
......................................................................uUgacggagaacugauaagggc.....................	256	1	Fm3
......................................................................uggacggaAaacugauaagggc.....................	51	1	Fm3
......................................................................uggacggagaacugGuaagggc.....................	95	1	Fm3
......................................................................uggacggagaacugaCaagggc.....................	82	1	Fm3
......................................................................uggacggagaacCgauaagggc.....................	90	1	Fm3
......................................................................uggacggagaacugauaUgggc.....................	2622	1	Fm3
......................................................................uggacggagaacugCuaagggc.....................	28	1	Fm3
......................................................................uggacggagaacugauaaggUc.....................	273	1	Fm3
......................................................................uggacggUgaacugauaagggc.....................	214	1	Fm3
......................................................................uggacggagaacugauaagggc.....................	790111	0	Fm3
......................................................................uggacggagaacugUuaagggc.....................	135	1	Fm3
......................................................................uggacggagaacugauNagggc.....................	3	1	Fm3
......................................................................uggacggagaaAugauaagggc.....................	34	1	Fm3
......................................................................uAgacggagaacugauaagggc.....................	387	1	Fm3
......................................................................uggacgNagaacugauaagggc.....................	41	1	Fm3
......................................................................uggaAggagaacugauaagggc.....................	91	1	Fm3
......................................................................uggacgUagaacugauaagggc.....................	263	1	Fm3
......................................................................uggacggagaacugauaagggG.....................	97	1	Fm3
......................................................................uggacggagGacugauaagggc.....................	581	1	Fm3
......................................................................uggacggGgaacugauaagggc.....................	180	1	Fm3
......................................................................ugUacggagaacugauaagggc.....................	244	1	Fm3
......................................................................uggacggagaacGgauaagggc.....................	25	1	Fm3
......................................................................uggacggagaacugauaaAggc.....................	51	1	Fm3
......................................................................uggacCgagaacugauaagggc.....................	40	1	Fm3
......................................................................uggacggagaaUugauaagggc.....................	189	1	Fm3
......................................................................uggCcggagaacugauaagggc.....................	161	1	Fm3
......................................................................uggacggagaacuUauaagggc.....................	83	1	Fm3
......................................................................uggacggagaCcugauaagggc.....................	696	1	Fm3
......................................................................Aggacggagaacugauaagggc.....................	237	1	Fm3
......................................................................uggacggagaaGugauaagggc.....................	15	1	Fm3
......................................................................uNgacggagaacugauaagggc.....................	10	1	Fm3
......................................................................uggacgAagaacugauaagggc.....................	78	1	Fm3
......................................................................uggacggagaacugauaCgggc.....................	3025	1	Fm3
......................................................................uggacAgagaacugauaagggc.....................	155	1	Fm3
......................................................................ugAacggagaacugauaagggc.....................	192	1	Fm3
......................................................................uggacggagaacugauaagggU.....................	12581	1	Fm3
......................................................................Gggacggagaacugauaagggc.....................	40	1	Fm3
......................................................................uggacUgagaacugauaagggc.....................	245	1	Fm3
......................................................................uggacggagaacugaAaagggc.....................	29	1	Fm3
......................................................................uggacggagaacuCauaagggc.....................	58	1	Fm3
......................................................................uggacNgagaacugauaagggca....................	17	1	Fm3
......................................................................uggacggagaaUugauaagggca....................	22	1	Fm3
......................................................................uggacUgagaacugauaagggca....................	51	1	Fm3
......................................................................uggacggagaacCgauaagggca....................	7	1	Fm3
......................................................................uAgacggagaacugauaagggca....................	78	1	Fm3
......................................................................uggacggagaacugauCagggca....................	330	1	Fm3
......................................................................uggacCgagaacugauaagggca....................	9	1	Fm3
```

Star **Mature**

cauuugugacaucagaaguaagucuguuucguguccucuucacuuuuccauccugucuguacucgagaacuggacggagaacugauaagggcauagggcagaccacaucaaca			
....uggCcggagaacugauaagggca....................	19	1	Fm3
....uggacggagaacugauaaggAca....................	29	1	Fm3
....uggacggagaacugauaaAggca....................	14	1	Fm3
....uggacggagaacugauaagggUa....................	363	1	Fm3
....uggacggagCacugauaagggca....................	20	1	Fm3
....ugNacggagaacugauaagggca....................	2	1	Fm3
....uggacggagaacAgauaagggca....................	7	1	Fm3
....uggacggagaacugauaUgggca....................	546	1	Fm3
....uggacggagaacugauaagggGa....................	30	1	Fm3
....uggacgNagaacugauaagggca....................	9	1	Fm3
....Nggacggagaacugauaagggca....................	77	1	Fm3
....uggacggagaacugauaaUggca....................	69	1	Fm3
....uggacggagaacugauaaggCca....................	16	1	Fm3
....uggacggagaacugauaagggcG....................	246	1	Fm3
....uggacggagUacugauaagggca....................	32	1	Fm3
....uggacggagaacugauaagAgca....................	26	1	Fm3
....uggacggagaacuAauaagggca....................	27	1	Fm3
....uggacggagaacuCauaagggca....................	9	1	Fm3
....uggacggaCaacugauaagggca....................	7	1	Fm3
....uggacggagaacugaCaagggca....................	24	1	Fm3
....uggacggUgaacugauaagggca....................	49	1	Fm3
....Gggacggagaacugauaagggca....................	4	1	Fm3
....uggacggagaacugauaaggUca....................	39	1	Fm3
....uggacggagaacugauaagUgca....................	52	1	Fm3
....uggacggGgaacugauaagggca....................	41	1	Fm3
....uggaUggagaacugauaagggca....................	59	1	Fm3
....uggacggagaacugauaagggAa....................	3429	1	Fm3
....uggGcggagaacugauaagggca....................	34	1	Fm3
....uggacggagaGcugauaagggca....................	257	1	Fm3
....uggacggagaacugUuaagggca....................	26	1	Fm3
....uggacgCagaacugauaagggca....................	11	1	Fm3
....uggacggagaacugauaGgggca....................	818	1	Fm3
....uCgacggagaacugauaagggca....................	17	1	Fm3
....uggacggaAaacugauaagggca....................	10	1	Fm3
....Cggacggagaacugauaagggca....................	37	1	Fm3
....uggacggagaacugauaCgggca....................	617	1	Fm3
....uggacggagaacugGuaagggca....................	19	1	Fm3
....uUgacggagaacugauaagggca....................	63	1	Fm3
....uggacggagaacuUauaagggca....................	14	1	Fm3
....uggacggagaUcugauaagggca....................	14451	1	Fm3
....uggacggagaacugauaagggcC....................	86	1	Fm3
....uggacggagaacugauaaCggca....................	8	1	Fm3
....ugCacggagaacugauaagggca....................	16	1	Fm3
....uggaGggagaacugauaagggca....................	16	1	Fm3
....uggUcggagaacugauaagggca....................	28	1	Fm3
....uggacggagaCcugauaagggca....................	127	1	Fm3
....uNgacggagaacugauaagggca....................	3	1	Fm3
....uggacggagaaGugauaagggca....................	6	1	Fm3
....ugAacggagaacugauaagggca....................	32	1	Fm3
....uggacggagaacugaGaagggca....................	6	1	Fm3
....uggacggagaacugauaagCgca....................	9	1	Fm3
....uggacgAagaacugauaagggca....................	9	1	Fm3
....uggacggCgaacugauaagggca....................	10	1	Fm3
....uggacgUagaacugauaagggca....................	59	1	Fm3
....uggacggaUaacugauaagggca....................	32	1	Fm3
....uggaAggagaacugauaagggca....................	19	1	Fm3
....uggacggagaacGgauaagggca....................	5	1	Fm3
....uggacggagGacugauaagggca....................	72	1	Fm3
....uggacggagaacugaAaagggca....................	2	1	Fm3
....ugUacggagaacugauaagggca....................	58	1	Fm3
....uggacggagaacugauaagggca....................	165997	0	Fm3
....uggacggagaacugauGagggca....................	343	1	Fm3
....Aggacggagaacugauaagggca....................	26	1	Fm3
....uggacggagaacugCuaagggca....................	7	1	Fm3
....uggacAgagaacugauaagggca....................	33	1	Fm3
....uggacggagaaAugauaagggca....................	6	1	Fm3
....uggacggagaacugauUagggca....................	198	1	Fm3
....ugCacggagaacugauaagggcau...................	2	1	Fm3
....Aggacggagaacugauaagggcau...................	2	1	Fm3
....uggacggagaCcugauaagggcau...................	2	1	Fm3

Star **Mature**

cauuugugacaucagaaguaagucuguuucguguccucuucacuuuuccauccugucuguacucgagaacuggacggagaacugauaagggcauagggcagaccacaucaaca			
....uggacggagaacugauaagggcCu...................	4	1	Fm3
....uggacggagaacugauaagCgcau...................	1	1	Fm3
....uggacggagaacugauCagggcau...................	15	1	Fm3
....uggacggagaacugaCaagggcau...................	1	1	Fm3
....uggacggagaacugauaagggcGu...................	5	1	Fm3
....uggacggagaaUugauaagggcau...................	1	1	Fm3
....uggacggagaacugauaagggcaA...................	231	1	Fm3
....uggacggagaacugauaGgggcau...................	26	1	Fm3
....uggacggagaacugauaagggUau...................	434	1	Fm3
....uggGcggagaacugauaagggcau...................	1	1	Fm3
....uggacggagaacCgauaagggcau...................	2	1	Fm3
....uggacggagaacugauaagggcau...................	4351	0	Fm3
....uggacUgagaacugauaagggcau...................	2	1	Fm3
....uggacNgagaacugauaagggcau...................	2	1	Fm3
....uggacggagaGcugauaagggcau...................	7	1	Fm3
....Nggacggagaacugauaagggcau...................	3	1	Fm3
....uggacggagaUcugauaagggcau...................	513	1	Fm3
....uggacggagaacugauaagggcaC...................	9	1	Fm3
....uggacggagaacugauaaUggcau...................	1	1	Fm3
....uggacggagaacugauaUgggcau...................	19	1	Fm3
....uggaUggagaacugauaagggcau...................	2	1	Fm3
....uggacggagaacugauGagggcau...................	7	1	Fm3
....uUgacggagaacugauaagggcau...................	1	1	Fm3
....uggacggagaacuAauaagggcau...................	2	1	Fm3
....uggacggagaacugauUagggcau...................	4	1	Fm3
....uggacggagaacugauaagAgcau...................	1	1	Fm3
....uggacggagaacugauaagggAau...................	2005	1	Fm3
....uggacggagCacugauaagggcau...................	1	1	Fm3
....uggacggagaacAgauaagggcau...................	1	1	Fm3
....uggacggagaacugauaCgggcau...................	16	1	Fm3
....uggaAggagaacugauaagggcau...................	2	1	Fm3
....uAgacggagaacugauaagggcau...................	2	1	Fm3
....uggacggagaacugauaagUgcau...................	1	1	Fm3
....ugUacggagaacugauaagggcau...................	1	1	Fm3
....uggacgUagaacugauaagggcau...................	3	1	Fm3
....uggacggagaacugauaagggcaG...................	63	1	Fm3
....uggacggagGacugauaagggcau...................	4	1	Fm3
....uggacggagaacugauaagggcauC..................	2	1	Fm3
....uggacggagaacugauaagggcaAa..................	7	1	Fm3
....uggacggagaacugauaagggcaGa..................	2	1	Fm3
....uggacggagaacugauaagggcUua..................	2	1	Fm3
....uggacggagaacugauaagggcauU..................	159	1	Fm3
....uggacggagaUcugauaagggcaua..................	3	1	Fm3
....uggacggagaacugauaagggcaua..................	7	0	Fm3
....uggaUggagaacugauaagggcaua..................	1	1	Fm3
....uggacggagaacugauaagggcauaU.................	12	1	Fm3
....ggGcggagaacugauaag........................	1	1	Fm3
....ggacggaAaacugauaag........................	1	1	Fm3
....ggaUggagaacugauaag........................	1	1	Fm3
....Ugacggagaacugauaag........................	6	1	Fm3
....ggacggagaacugauaaC........................	1	1	Fm3
....ggacggagaacugauaag........................	135	0	Fm3
....ggacggagaacugauaaA........................	68	1	Fm3
....ggacggagaacugauaCg........................	1	1	Fm3
....ggacggagCacugauaag........................	1	1	Fm3
....ggacggagaacugaAaag........................	1	1	Fm3
....ggacggagaacugauaaU........................	21	1	Fm3
....ggacggagaacugaGaag........................	1	1	Fm3
....ggacggagaacugauCag........................	1	1	Fm3
....ggacggagaaUugauaagg.......................	1	1	Fm3
....ggacggagaacugaAaagg.......................	1	1	Fm3
....Ugacggagaacugauaagg.......................	9	1	Fm3
....ggacggagaacCgauaagg.......................	2	1	Fm3
....ggacggagaacugauaGgg.......................	2	1	Fm3
....ggacggagaacugauCagg.......................	3	1	Fm3
....ggacggagaacGgauaagg.......................	1	1	Fm3
....ggacggagaacugauaUgg.......................	1	1	Fm3
....ggacggagaacugauaagU.......................	8	1	Fm3
....ggacggagaacugauaaAg.......................	1	1	Fm3
....ggacggCgaacugauaagg.......................	1	1	Fm3

```
                                   Star                                Mature
cauuugugacaucagaaguaagucuguuucguguccucuucacuuuuccauccugucuguacucgagaacuggacggagaacugauaagggcauagggcagaccacaucaaca
.......................................................................ggacggagaacugauaagA.......................	108	1	Fm3
.......................................................................ggacggagaaAugauaagg.......................	3	1	Fm3
.......................................................................ggaAggagaacugauaagg.......................	1	1	Fm3
.......................................................................Ngacggagaacugauaagg.......................	1	1	Fm3
.......................................................................ggGcggagaacugauaagg.......................	1	1	Fm3
.......................................................................ggacggagaacugauaagg.......................	317	0	Fm3
.......................................................................ggacggagaGcugauaagg.......................	6	1	Fm3
.......................................................................ggacggagaCcugauaagg.......................	7	1	Fm3
.......................................................................ggacggaCaacugauaagg.......................	1	1	Fm3
.......................................................................ggacggagaacugauaaUg.......................	2	1	Fm3
.......................................................................ggacggaAaacugauaagg.......................	1	1	Fm3
.......................................................................ggacggagaacugauaaggA......................	253	1	Fm3
.......................................................................ggacggagaacGgauaaggg......................	1	1	Fm3
.......................................................................ggacggUgaacugauaaggg......................	1	1	Fm3
.......................................................................ggacggagaacugauaCggg......................	3	1	Fm3
.......................................................................ggacggagaCcugauaaggg......................	5	1	Fm3
.......................................................................ggacggagaacCgauaaggg......................	2	1	Fm3
.......................................................................ggacggagaacugauaaggC......................	5	1	Fm3
.......................................................................ggacggagaacugauaaggg......................	386	0	Fm3
.......................................................................ggacggagUacugauaaggg......................	1	1	Fm3
.......................................................................ggacggagaacugaAaaggg......................	1	1	Fm3
.......................................................................ggacggagaacugauaGggg......................	3	1	Fm3
.......................................................................ggacggagaaAugauaaggg......................	1	1	Fm3
.......................................................................ggacgAagaacugauaaggg......................	1	1	Fm3
.......................................................................ggacggagaacugauCaggg......................	5	1	Fm3
.......................................................................ggacggagaacugGuaaggg......................	1	1	Fm3
.......................................................................ggacggagCacugauaaggg......................	1	1	Fm3
.......................................................................ggacggagaacugUuaaggg......................	1	1	Fm3
.......................................................................ggacggagaacugauUaggg......................	2	1	Fm3
.......................................................................ggacggagaacugauaaggU......................	16	1	Fm3
.......................................................................ggacggagaacugauaaAgg......................	1	1	Fm3
.......................................................................Ugacggagaacugauaaggg......................	14	1	Fm3
.......................................................................ggacggagaaGugauaaggg......................	2	1	Fm3
.......................................................................ggacggagaGcugauaaggg......................	4	1	Fm3
.......................................................................ggacggagaacugauaagggc.....................	1615	0	Fm3
.......................................................................ggGcggagaacugauaagggc.....................	3	1	Fm3
.......................................................................ggCcggagaacugauaagggc.....................	1	1	Fm3
.......................................................................ggacggagaacugauaGgggc.....................	7	1	Fm3
.......................................................................ggacggagaaAugauaagggc.....................	7	1	Fm3
.......................................................................ggacggagaacugauaagggU.....................	23	1	Fm3
.......................................................................ggacggagaacugauaUgggc.....................	1	1	Fm3
.......................................................................ggacggagaacugauaagggA.....................	230	1	Fm3
.......................................................................Ngacggagaacugauaagggc.....................	1	1	Fm3
.......................................................................ggacggaAaacugauaagggc.....................	2	1	Fm3
.......................................................................ggacggagaacugauaaggAc.....................	8	1	Fm3
.......................................................................ggacggagaacugauaagAgc.....................	3	1	Fm3
.......................................................................ggacggUgaacugauaagggc.....................	1	1	Fm3
.......................................................................ggacggagaacugauaaAggc.....................	2	1	Fm3
.......................................................................ggaAggagaacugauaagggc.....................	4	1	Fm3
.......................................................................ggacCgagaacugauaagggc.....................	3	1	Fm3
.......................................................................ggacggagaCcugauaagggc.....................	4	1	Fm3
.......................................................................ggacggagaacAgauaagggc.....................	1	1	Fm3
.......................................................................ggacggagGacugauaagggc.....................	1	1	Fm3
.......................................................................ggacggagaacugauGagggc.....................	2	1	Fm3
.......................................................................ggacggagaacugauaCgggc.....................	3	1	Fm3
.......................................................................Ugacggagaacugauaagggc.....................	34	1	Fm3
.......................................................................ggacggagCacugauaagggc.....................	15	1	Fm3
.......................................................................ggacggagaacugaGaagggc.....................	2	1	Fm3
.......................................................................ggacggagaacugaAaagggc.....................	1	1	Fm3
.......................................................................ggacggCgaacugauaagggc.....................	6	1	Fm3
.......................................................................ggacggagaacugauUagggc.....................	2	1	Fm3
.......................................................................ggacggagaacugauaaggUc.....................	6	1	Fm3
.......................................................................ggacggagaaGugauaagggc.....................	4	1	Fm3
.......................................................................ggacggagaGcugauaagggc.....................	3	1	Fm3
.......................................................................ggacggagaacugUuaagggc.....................	1	1	Fm3
.......................................................................ggacggagaaUugauaagggc.....................	3	1	Fm3
.......................................................................ggacggagaacGgauaagggc.....................	6	1	Fm3
.......................................................................ggacggaCaacugauaagggc.....................	1	1	Fm3
.......................................................................ggacggagaacugauaaggCc.....................	7	1	Fm3
.......................................................................ggacggagaacugauaaUggc.....................	1	1	Fm3
```

Star Mature

```
cauuugugacaucagaaguaagucuguuucguguccucuucacuuuuccauccugucuguacucgagaacuggacggagaacugauaagggcauagggcagaccacaucaaca
.......................................................................ggacggagaacCgauaagggc.....................  1  1  Fm3
.......................................................................ggacggagUacugauaagggc.....................  2  1  Fm3
.......................................................................ggaGggagaacugauaagggc.....................  1  1  Fm3
.......................................................................ggacggagaacuCauaagggc.....................  1  1  Fm3
.......................................................................ggacggagaacugauaagCgc.....................  1  1  Fm3
.......................................................................ggacggagaacuUauaagggc.....................  2  1  Fm3
.......................................................................ggacggagaacugCuaagggca....................  1  1  Fm3
.......................................................................ggacggagaacugauaagUgca....................  1  1  Fm3
.......................................................................ggacggagaacuAauaagggca....................  1  1  Fm3
.......................................................................ggGcggagaacugauaagggca....................  1  1  Fm3
.......................................................................ggacggagaacugauaaggUca....................  1  1  Fm3
.......................................................................ggacggagaacugauGagggca....................  1  1  Fm3
.......................................................................ggacggagaaAugauaagggca....................  1  1  Fm3
.......................................................................ggacggagaacugauaaUggca....................  2  1  Fm3
.......................................................................ggacggagaacugauaagggca....................  334  0  Fm3
.......................................................................ggaAggagaacugauaagggca....................  1  1  Fm3
.......................................................................Ugacggagaacugauaagggca....................  11  1  Fm3
.......................................................................ggacggagCacugauaagggca....................  3  1  Fm3
.......................................................................ggacggagaacugauaagggAa....................  2  1  Fm3
.......................................................................ggacggagaacuCauaagggca....................  1  1  Fm3
.......................................................................ggacggagaacuUauaagggca....................  2  1  Fm3
.......................................................................ggacggagaacugauaaggAca....................  1  1  Fm3
.......................................................................ggacggagaUcugauaagggca....................  46  1  Fm3
.......................................................................ggacggagaacugauaaggCca....................  1  1  Fm3
.......................................................................ggacggagaaUugauaagggca....................  2  1  Fm3
.......................................................................ggacggagaacugauCagggca....................  1  1  Fm3
.......................................................................ggacggagaacugauaagggcau...................  11  0  Fm3
.......................................................................ggacggagaacuCauaagggcau...................  1  1  Fm3
.......................................................................ggacggagaacugauaagggAau...................  5  1  Fm3
.......................................................................ggacggagaUcugauaagggcau...................  3  1  Fm3
.......................................................................ggacggagaacugauaagggcaA...................  1  1  Fm3
.......................................................................ggacggagaacugauaagggcaC...................  1  1  Fm3
........................................................................Uacggagaacugauaaggg......................  3  1  Fm3
........................................................................gacggagaacugauaaggg......................  3  0  Fm3
........................................................................gacggagaaUugauaagggc.....................  2  1  Fm3
........................................................................gacggagaacugauaagggA.....................  1  1  Fm3
........................................................................gacggagaacugauaagggc.....................  9  0  Fm3
........................................................................Uacggagaacugauaagggc.....................  6  1  Fm3
.........................................................................acggagaacugauaagggc.....................  1  0  Fm3
..........................................................................Uggagaacugauaagggc.....................  10  1  Fm3
..........................................................................cggagaacugauaagggc.....................  1  0  Fm3
..........................................................................Uggagaacugauaagggca....................  3  1  Fm3
...........................................................................ggagaUcugauaagggca....................  1  1  Fm3
...........................................................................ggagaacugauaagggca....................  2  0  Fm3

..................................ccucuucacuuuuccauccugu.........................................................  8  0  Mm3
..................................ccucuucacuuuuccauccugucug......................................................  1  0  Mm3
...............................................................cgagaacuggacggagaacugauaagggc.....................  1  0  Mm3
....................................................................Ucuggacggagaacugauaagggc.....................  6  1  Mm3
.....................................................................cuggacggagaacugaua..........................  1  0  Mm3
.....................................................................cuggacggagaacugauaa.........................  3  0  Mm3
.....................................................................cuggacggagaacugauaaA........................  5  1  Mm3
.....................................................................cuggacggagaacugauaag........................  13  0  Mm3
.....................................................................cuggacggagaacugauaagA.......................  9  1  Mm3
.....................................................................cuggacggagaacugauUagg.......................  1  1  Mm3
.....................................................................cuggacggagaacugauaagg.......................  18  0  Mm3
.....................................................................cuggaNggagaacugauaagg.......................  1  1  Mm3
.....................................................................cuggacggagaacugauaaggg......................  33  0  Mm3
.....................................................................cuggacggagaacugauaaggA......................  4  1  Mm3
.....................................................................cuggacggagaacugauaaggC......................  1  1  Mm3
.....................................................................cuggacggagaacugauaaggU......................  3  1  Mm3
.....................................................................cuggacggagaacugauCaggg......................  2  1  Mm3
.....................................................................cuggacggagaacugauaGggg......................  1  1  Mm3
.....................................................................cuggacggagaacugauaagggA.....................  2  1  Mm3
.....................................................................cuggacggagaacugauaagggU.....................  2  1  Mm3
.....................................................................cuggCcggagaacugauaagggc.....................  1  1  Mm3
.....................................................................cAggacggagaacugauaagggc.....................  2  1  Mm3
.....................................................................cuggacggagaacugauaagggc.....................  112  0  Mm3
.....................................................................cuggacggagaacugauaagggca....................  7  0  Mm3
.....................................................................cuggacggagaUcugauaagggca....................  2  1  Mm3
```

```
                                  Star                                Mature
cauuugugacaucagaaguaagucuguuucguguccucuucacuuuuccauccugucuguacucgagaacuggacggagaacugauaagggcauagggcagaccacaucaaca
......................................................................uggacggagaaUugauaa.........................   1      1   Mm3
......................................................................uggacggagaacugauGa.........................   2      1   Mm3
......................................................................uggacggagaacCgauaa.........................   1      1   Mm3
......................................................................uggacggagaacugauaU.........................   62     1   Mm3
......................................................................uggacggagaacugaAaa.........................   66     1   Mm3
......................................................................uggacggagaGcugauaa.........................   3      1   Mm3
......................................................................uggacggagaacugauCa.........................   6      1   Mm3
......................................................................uggacggagCacugauaa.........................   1      1   Mm3
......................................................................uggacUgagaacugauaa.........................   1      1   Mm3
......................................................................uggacggaCaacugauaa.........................   1      1   Mm3
......................................................................uggacggUgaacugauaa.........................   1      1   Mm3
......................................................................uggacggagaacugauaG.........................   13     1   Mm3
......................................................................uggacggagaacugaCaa.........................   2      1   Mm3
......................................................................Nggacggagaacugauaa.........................   2      1   Mm3
......................................................................uggacggagaacugauaC.........................   4      1   Mm3
......................................................................uggacggagaacugauaa.........................   2074   0   Mm3
......................................................................uggaUggagaacugauaa.........................   2      1   Mm3
......................................................................Aggacggagaacugauaa.........................   2      1   Mm3
......................................................................uggacggagaacugauUa.........................   5      1   Mm3
......................................................................uggacggagaCcugauaa.........................   1      1   Mm3
......................................................................uggCcggagaacugauaa.........................   1      1   Mm3
......................................................................uggacggagaacugauaaU........................   1351   1   Mm3
......................................................................uggacggagaacugauUag........................   14     1   Mm3
......................................................................uggacggagaacugauCag........................   11     1   Mm3
......................................................................uggacggagaacugCuaag........................   1      1   Mm3
......................................................................uAgacggagaacugauaag........................   3      1   Mm3
......................................................................ugAacggagaacugauaag........................   4      1   Mm3
......................................................................uggacggagaacugauaaC........................   15     1   Mm3
......................................................................uggacggagaacugUuaag........................   1      1   Mm3
......................................................................uggacggagaacCgauaag........................   1      1   Mm3
......................................................................Cggacggagaacugauaag........................   2      1   Mm3
......................................................................uggacggagaacugauaaA........................   5374   1   Mm3
......................................................................uggCcggagaacugauaag........................   1      1   Mm3
......................................................................uggacggagaacugauaUg........................   30     1   Mm3
......................................................................uggacggaUaacugauaag........................   2      1   Mm3
......................................................................uggacggagaacuAauaag........................   4      1   Mm3
......................................................................uggacAgagaacugauaag........................   4      1   Mm3
......................................................................uggacggUgaacugauaag........................   3      1   Mm3
......................................................................uggacggagaacugauGag........................   10     1   Mm3
......................................................................Aggacggagaacugauaag........................   11     1   Mm3
......................................................................uggacggagGacugauaag........................   9      1   Mm3
......................................................................uggacggagUacugauaag........................   4      1   Mm3
......................................................................uggacggagaacugauaCg........................   30     1   Mm3
......................................................................Nggacggagaacugauaag........................   3      1   Mm3
......................................................................uggacggagaGcugauaag........................   30     1   Mm3
......................................................................uggacggagaaUugauaag........................   2      1   Mm3
......................................................................uggUcggagaacugauaag........................   1      1   Mm3
......................................................................uggacUgagaacugauaag........................   2      1   Mm3
......................................................................uggacggagaaAugauaag........................   1      1   Mm3
......................................................................uggacggagaCcugauaag........................   14     1   Mm3
......................................................................uggacggGgaacugauaag........................   3      1   Mm3
......................................................................uggaAggagaacugauaag........................   1      1   Mm3
......................................................................uggacggagaacugaAaag........................   3      1   Mm3
......................................................................uggacggagaacuUauaag........................   4      1   Mm3
......................................................................uggacggagaacugaCaag........................   1      1   Mm3
......................................................................uggacggagaacugauaGg........................   40     1   Mm3
......................................................................uggacggagaacugauaag........................   8431   0   Mm3
......................................................................uggGcggagaacugauaag........................   1      1   Mm3
......................................................................uUgacggagaacugauaag........................   2      1   Mm3
......................................................................uggacggagCacugauaag........................   1      1   Mm3
......................................................................uggacggagUacugauaagg.......................   2      1   Mm3
......................................................................uggacggagaacugauGagg.......................   21     1   Mm3
......................................................................uCgacggagaacugauaagg.......................   1      1   Mm3
......................................................................uggacggagaacugauaaUg.......................   2      1   Mm3
......................................................................uggacggagaacugUuaagg.......................   3      1   Mm3
......................................................................uAgacggagaacugauaagg.......................   11     1   Mm3
......................................................................uggacggagaacugauCagg.......................   31     1   Mm3
......................................................................uggacggaCaacugauaagg.......................   1      1   Mm3
......................................................................uggacggagaacugauaagA.......................   3650   1   Mm3
......................................................................uggacggagaacugauaGgg.......................   91     1   Mm3
```

```
                                  Star                                Mature
cauuugugacaucagaaguaagucuguuucguguccucuucacuuuuccauccugucuguacucgagaacuggacggagaacugauaagggcauagggcagaccacaucaaca
......................................................................uggacggagaacugauUagg.......................    12      1     Mm3
......................................................................uggacggagaaUugauaagg.......................    3       1     Mm3
......................................................................uUgacggagaacugauaagg.......................    7       1     Mm3
......................................................................uggacggagaacugauaagC.......................    10      1     Mm3
......................................................................uggacggagaacugauaagU.......................    617     1     Mm3
......................................................................Gggacggagaacugauaagg.......................    1       1     Mm3
......................................................................uggCcggagaacugauaagg.......................    3       1     Mm3
......................................................................uggacggagaacGgauaagg.......................    2       1     Mm3
......................................................................uggacggagGacugauaagg.......................    27      1     Mm3
......................................................................uggacggagaacCgauaagg.......................    1       1     Mm3
......................................................................uggacggaUaacugauaagg.......................    1       1     Mm3
......................................................................uggacAgagaacugauaagg.......................    2       1     Mm3
......................................................................uggGcggagaacugauaagg.......................    5       1     Mm3
......................................................................uggaUggagaacugauaagg.......................    7       1     Mm3
......................................................................uggacggagaacuAauaagg.......................    1       1     Mm3
......................................................................ugCacggagaacugauaagg.......................    2       1     Mm3
......................................................................uggacggagaacugGuaagg.......................    1       1     Mm3
......................................................................uggacggagaacugauaCgg.......................    58      1     Mm3
......................................................................uggacggUgaacugauaagg.......................    6       1     Mm3
......................................................................uNgacggagaacugauaagg.......................    1       1     Mm3
......................................................................uggacggagaacuUauaagg.......................    1       1     Mm3
......................................................................uggacggCgaacugauaagg.......................    2       1     Mm3
......................................................................uggacgAagaacugauaagg.......................    2       1     Mm3
......................................................................uggacggagaacugauaaAg.......................    91      1     Mm3
......................................................................uggacggagaCcugauaagg.......................    76      1     Mm3
......................................................................uggacggagCacugauaagg.......................    5       1     Mm3
......................................................................uggacggagaacugauaUgg.......................    64      1     Mm3
......................................................................uggacNgagaacugauaagg.......................    3       1     Mm3
......................................................................uggacggaAaacugauaagg.......................    2       1     Mm3
......................................................................uggacCgagaacugauaagg.......................    1       1     Mm3
......................................................................ugUacggagaacugauaagg.......................    3       1     Mm3
......................................................................uggacgUagaacugauaagg.......................    1       1     Mm3
......................................................................uggacggGgaacugauaagg.......................    2       1     Mm3
......................................................................Nggacggagaacugauaagg.......................    6       1     Mm3
......................................................................Aggacggagaacugauaagg.......................    140     1     Mm3
......................................................................uggacggagaGcugauaagg.......................    140     1     Mm3
......................................................................uggacUgagaacugauaagg.......................    11      1     Mm3
......................................................................Cggacggagaacugauaagg.......................    4       1     Mm3
......................................................................uggacggagaaAugauaagg.......................    1       1     Mm3
......................................................................uggacggagaacugauaagg.......................    14584   0     Mm3
......................................................................uggacgCagaacugauaagg.......................    3       1     Mm3
......................................................................uggUcggagaacugauaagg.......................    4       1     Mm3
......................................................................uUgacggagaacugauaaggg......................    5       1     Mm3
......................................................................uggacggagaacugauaaAgg......................    3       1     Mm3
......................................................................ugAacggagaacugauaaggg......................    7       1     Mm3
......................................................................uggacggagaacugauGaggg......................    40      1     Mm3
......................................................................uggacggGgaacugauaaggg......................    11      1     Mm3
......................................................................uggacggagaacugauaaUgg......................    3       1     Mm3
......................................................................Cggacggagaacugauaaggg......................    11      1     Mm3
......................................................................uAgacggagaacugauaaggg......................    9       1     Mm3
......................................................................ugUacggagaacugauaaggg......................    3       1     Mm3
......................................................................uggacNgagaacugauaaggg......................    2       1     Mm3
......................................................................uggacggagaacCgauaaggg......................    4       1     Mm3
......................................................................uggacggagaacugauaaggU......................    657     1     Mm3
......................................................................uggacggagaacugauaaggg......................    27231   0     Mm3
......................................................................uggacggagaacugauaGggg......................    205     1     Mm3
......................................................................uggacggagaacugUuaaggg......................    5       1     Mm3
......................................................................uggacgNagaacugauaaggg......................    2       1     Mm3
......................................................................uggacUgagaacugauaaggg......................    13      1     Mm3
......................................................................uggacggagaacugaCaaggg......................    1       1     Mm3
......................................................................uggacggUgaacugauaaggg......................    15      1     Mm3
......................................................................uggacggagaacuAauaaggg......................    6       1     Mm3
......................................................................uggacggaUaacugauaaggg......................    6       1     Mm3
......................................................................uggacggagaCcugauaaggg......................    71      1     Mm3
......................................................................uggCcggagaacugauaaggg......................    3       1     Mm3
......................................................................uggaAggagaacugauaaggg......................    2       1     Mm3
......................................................................uggacggagaacugaAaaggg......................    1       1     Mm3
......................................................................uggacggagaacugauaagAg......................    28      1     Mm3
......................................................................uggacAgagaacugauaaggg......................    5       1     Mm3
......................................................................uggacggagaacugauCaggg......................    60      1     Mm3
```

```
                                  Star                              Mature
cauuugugacaucagaaguaagucuguuucguguccucuucacuuuuccauccugucuguacucgagaacuggacggagaacugauaagggcauagggcagaccacaucaaca
......................................................................Gggacggagaacugauaaggg......................   1       1   Mm3
......................................................................Nggacggagaacugauaaggg......................   13      1   Mm3
......................................................................uggUcggagaacugauaaggg......................   3       1   Mm3
......................................................................uggacggagaacugauaCggg......................   118     1   Mm3
......................................................................uggacggagaacugauaagUg......................   2       1   Mm3
......................................................................uggacggaCaacugauaaggg......................   1       1   Mm3
......................................................................Aggacggagaacugauaaggg......................   102     1   Mm3
......................................................................uggacggagaacugauUaggg......................   38      1   Mm3
......................................................................uggacggagCacugauaaggg......................   5       1   Mm3
......................................................................uggacggagaacuUauaaggg......................   5       1   Mm3
......................................................................uggacggaAaacugauaaggg......................   1       1   Mm3
......................................................................uggacggagaaAugauaaggg......................   2       1   Mm3
......................................................................uggacggagGacugauaaggg......................   35      1   Mm3
......................................................................uggacggagaacuCauaaggg......................   2       1   Mm3
......................................................................uggacgUagaacugauaaggg......................   5       1   Mm3
......................................................................uggacggagaacugauaaggA......................   4536    1   Mm3
......................................................................uggacggCgaacugauaaggg......................   4       1   Mm3
......................................................................uggacggagaacugCuaaggg......................   1       1   Mm3
......................................................................uCgacggagaacugauaaggg......................   1       1   Mm3
......................................................................uggacggagUacugauaaggg......................   9       1   Mm3
......................................................................uggacCgagaacugauaaggg......................   2       1   Mm3
......................................................................uggacggagaaUugauaaggg......................   7       1   Mm3
......................................................................uggacggagaacugauaUggg......................   74      1   Mm3
......................................................................uggacgAagaacugauaaggg......................   5       1   Mm3
......................................................................uggaUggagaacugauaaggg......................   3       1   Mm3
......................................................................uggGcggagaacugauaaggg......................   6       1   Mm3
......................................................................uggacggagaacugGuaaggg......................   1       1   Mm3
......................................................................uggacggagaacugauaagCg......................   1       1   Mm3
......................................................................uggacggagaacAgauaaggg......................   3       1   Mm3
......................................................................uggacggagaacugauaaggC......................   89      1   Mm3
......................................................................uggacggagaGcugauaaggg......................   145     1   Mm3
......................................................................uggacggaUaacugauaagggc.....................   47      1   Mm3
......................................................................uggacggagaGcugauaagggc.....................   541     1   Mm3
......................................................................uNgacggagaacugauaagggc.....................   3       1   Mm3
......................................................................uggacggagaacugauaCgggc.....................   1119    1   Mm3
......................................................................uggacggagaacugauaagggA.....................   8184    1   Mm3
......................................................................uggacggagaacugauCagggc.....................   629     1   Mm3
......................................................................uggacggagaacugaAaagggc.....................   10      1   Mm3
......................................................................uggaUggagaacugauaagggc.....................   74      1   Mm3
......................................................................uggacgUagaacugauaagggc.....................   53      1   Mm3
......................................................................uggaAggagaacugauaagggc.....................   29      1   Mm3
......................................................................uUgacggagaacugauaagggc.....................   58      1   Mm3
......................................................................uggacUgagaacugauaagggc.....................   32      1   Mm3
......................................................................uggacggagaacugauaaggCc.....................   161     1   Mm3
......................................................................Cggacggagaacugauaagggc.....................   55      1   Mm3
......................................................................uggacgAagaacugauaagggc.....................   36      1   Mm3
......................................................................uggacggagaacugaGaagggc.....................   2       1   Mm3
......................................................................Nggacggagaacugauaagggc.....................   148     1   Mm3
......................................................................uggacggagaacugauUagggc.....................   428     1   Mm3
......................................................................uggacggagaacugauaagCgc.....................   22      1   Mm3
......................................................................uggacggagaacugauaagAgc.....................   49      1   Mm3
......................................................................uggacggagCacugauaagggc.....................   54      1   Mm3
......................................................................uggGcggagaacugauaagggc.....................   58      1   Mm3
......................................................................uggacggagaCcugauaagggc.....................   247     1   Mm3
......................................................................uggacggagaacugauaagggG.....................   144     1   Mm3
......................................................................ugCacggagaacugauaagggc.....................   24      1   Mm3
......................................................................uggacggagaacugauaaCggc.....................   7       1   Mm3
......................................................................ugAacggagaacugauaagggc.....................   81      1   Mm3
......................................................................uggacggagUacugauaagggc.....................   126     1   Mm3
......................................................................uCgacggagaacugauaagggc.....................   32      1   Mm3
......................................................................uggacggagaacugaCaagggc.....................   20      1   Mm3
......................................................................Aggacggagaacugauaagggc.....................   284     1   Mm3
......................................................................uggacggagaaAugauaagggc.....................   9       1   Mm3
......................................................................ugNacggagaacugauaagggc.....................   2       1   Mm3
......................................................................uggacggagaacuAauaagggc.....................   60      1   Mm3
......................................................................uggacggaAaacugauaagggc.....................   19      1   Mm3
......................................................................uggacggGgaacugauaagggc.....................   76      1   Mm3
......................................................................uggacggagaacAgauaagggc.....................   17      1   Mm3
......................................................................uggacggagaacugauaagggc.....................   293785  0   Mm3
......................................................................uggacggagaacGgauaagggc.....................   12      1   Mm3
```

```
                                                                  Star                              Mature
cauuugugacaucagaaguaagucuguuucguguccucuucacuuuuccauccugucuguacucgagaacuggacggagaacugauaagggcauagggcagaccacaucaaca
......................................................................uggacggagaacugauaaAggc.....................    31      1    Mm3
......................................................................uggacggagaacugauaagUgc.....................    69      1    Mm3
......................................................................uggacggagaacugCuaagggc.....................    9       1    Mm3
......................................................................uggacggagaacugauaNgggc.....................    2       1    Mm3
......................................................................uggacggagaacugauaaUggc.....................    50      1    Mm3
......................................................................uggacggagaacugauaGgggc.....................    2855    1    Mm3
......................................................................uggacCgagaacugauaagggc.....................    7       1    Mm3
......................................................................uggacggagaacugauaaggAc.....................    60      1    Mm3
......................................................................uggacggagaacuCauaagggc.....................    19      1    Mm3
......................................................................uggacggCgaacugauaagggc.....................    43      1    Mm3
......................................................................uggacggagaacugUuaagggc.....................    56      1    Mm3
......................................................................uggacggagaaUugauaagggc.....................    74      1    Mm3
......................................................................uggacggagaacCgauaagggc.....................    35      1    Mm3
......................................................................uggacggagaacugGuaagggc.....................    29      1    Mm3
......................................................................uggacggUgaacugauaagggc.....................    168     1    Mm3
......................................................................Gggacggagaacugauaagggc.....................    15      1    Mm3
......................................................................uggUcggagaacugauaagggc.....................    42      1    Mm3
......................................................................uggCcggagaacugauaagggc.....................    70      1    Mm3
......................................................................uggacggagGacugauaagggc.....................    464     1    Mm3
......................................................................uggacggagaacugauaUgggc.....................    986     1    Mm3
......................................................................uggacggaCaacugauaagggc.....................    13      1    Mm3
......................................................................uggacggagaacugauaagggU.....................    2911    1    Mm3
......................................................................uggacgNagaacugauaagggc.....................    22      1    Mm3
......................................................................ugUacggagaacugauaagggc.....................    39      1    Mm3
......................................................................uggacNgagaacugauaagggc.....................    38      1    Mm3
......................................................................uggacggagaacugauGagggc.....................    556     1    Mm3
......................................................................uggacggagaacugauaaggUc.....................    74      1    Mm3
......................................................................uggacggagaacuUauaagggc.....................    28      1    Mm3
......................................................................uggacgCagaacugauaagggc.....................    17      1    Mm3
......................................................................uAgacggagaacugauaagggc.....................    111     1    Mm3
......................................................................uggaGggagaacugauaagggc.....................    14      1    Mm3
......................................................................uggacAgagaacugauaagggc.....................    50      1    Mm3
......................................................................uggacggagaaGugauaagggc.....................    6       1    Mm3
......................................................................uggacAgagaacugauaagggca....................    5       1    Mm3
......................................................................uggacggCgaacugauaagggca....................    4       1    Mm3
......................................................................uggacggagaacuUauaagggca....................    2       1    Mm3
......................................................................uUgacggagaacugauaagggca....................    6       1    Mm3
......................................................................uggacggagaacugauaaggAca....................    4       1    Mm3
......................................................................uggacggagaacugauaagggca....................    26505   0    Mm3
......................................................................ugAacggagaacugauaagggca....................    5       1    Mm3
......................................................................uggacCgagaacugauaagggca....................    2       1    Mm3
......................................................................uggacggagUacugauaagggca....................    8       1    Mm3
......................................................................uggacggagaacugauaaCggca....................    1       1    Mm3
......................................................................uggacggagaacugauaagUgca....................    5       1    Mm3
......................................................................uggacggagaacugauaagggcG....................    434     1    Mm3
......................................................................Aggacggagaacugauaagggca....................    14      1    Mm3
......................................................................uCgacggagaacugauaagggca....................    2       1    Mm3
......................................................................uggacggaUaacugauaagggca....................    4       1    Mm3
......................................................................uggacggagaacugauaCgggca....................    92      1    Mm3
......................................................................uggacggagaacuAauaagggca....................    5       1    Mm3
......................................................................uggacggagaacugauaagggGa....................    3       1    Mm3
......................................................................uggacggagaacAgauaagggca....................    2       1    Mm3
......................................................................uggacggagaaUugauaagggca....................    12      1    Mm3
......................................................................uggacggagaacugauaaggUca....................    7       1    Mm3
......................................................................uggaAggagaacugauaagggca....................    2       1    Mm3
......................................................................uggacggagCacugauaagggca....................    8       1    Mm3
......................................................................uggacNgagaacugauaagggca....................    4       1    Mm3
......................................................................uggacggagGacugauaagggca....................    29      1    Mm3
......................................................................uggacggagaacugauaagggAa....................    420     1    Mm3
......................................................................uggacUgagaacugauaagggca....................    3       1    Mm3
......................................................................Cggacggagaacugauaagggca....................    4       1    Mm3
......................................................................uggacggagaacugauaagCgca....................    2       1    Mm3
......................................................................uggacggagaacugauaagggcC....................    175     1    Mm3
......................................................................ugCacggagaacugauaagggca....................    2       1    Mm3
......................................................................uggacggagaacugauGagggca....................    54      1    Mm3
......................................................................uggacggGgaacugauaagggca....................    4       1    Mm3
......................................................................uggacggagaacugauaagAgca....................    10      1    Mm3
......................................................................uggacggaCaacugauaagggca....................    1       1    Mm3
......................................................................uggGcggagaacugauaagggca....................    4       1    Mm3
......................................................................uggacggagaacugauaGgggca....................    185     1    Mm3
```

Star **Mature**

```
cauuugugacaucagaaguaagucuguuucguguccucuucacuuuuccauccugucuguacucgagaacuggacggagaacugauaagggcauagggcagaccacaucaaca
......................................................................uggacggagaacugauCagggca....................	79	1	Mm3
......................................................................Nggacggagaacugauaagggca....................	10	1	Mm3
......................................................................uAgacggagaacugauaagggca....................	9	1	Mm3
......................................................................uggacggagaacugUuaagggca....................	5	1	Mm3
......................................................................uggCcggagaacugauaagggca....................	2	1	Mm3
......................................................................uggacggagaacugauaaUggca....................	4	1	Mm3
......................................................................uggacggagaacugauaagggUa....................	75	1	Mm3
......................................................................uggacgNagaacugauaagggca....................	3	1	Mm3
......................................................................uggacggagaCcugauaagggca....................	22	1	Mm3
......................................................................uggUcggagaacugauaagggca....................	5	1	Mm3
......................................................................uggacggagaGcugauaagggca....................	44	1	Mm3
......................................................................uggaGggagaacugauaagggca....................	1	1	Mm3
......................................................................uggacggagaacugauaaggCca....................	26	1	Mm3
......................................................................uggacggagaacugGuaagggca....................	4	1	Mm3
......................................................................uggacggagaacugauaUgggca....................	78	1	Mm3
......................................................................uggacggagaUcugauaagggca....................	2237	1	Mm3
......................................................................uggacggagaacugaCaagggca....................	6	1	Mm3
......................................................................ugUacggagaacugauaagggca....................	3	1	Mm3
......................................................................uggacggagaacugaAaagggca....................	1	1	Mm3
......................................................................uggaUggagaacugauaagggca....................	6	1	Mm3
......................................................................uggacggagaacugauUagggca....................	35	1	Mm3
......................................................................uggacggUgaacugauaagggca....................	11	1	Mm3
......................................................................uggacgUagaacugauaagggca....................	3	1	Mm3
......................................................................uggacgAagaacugauaagggca....................	2	1	Mm3
......................................................................uggacggagaacugauaagUgcau...................	1	1	Mm3
......................................................................uggCcggagaacugauaagggcau...................	1	1	Mm3
......................................................................uggUcggagaacugauaagggcau...................	1	1	Mm3
......................................................................uCgacggagaacugauaagggcau...................	1	1	Mm3
......................................................................uggacggUgaacugauaagggcau...................	2	1	Mm3
......................................................................uggacggagaacugauaagggcaA...................	59	1	Mm3
......................................................................uggacggaUaacugauaagggcau...................	1	1	Mm3
......................................................................uggacggagaUcugauaagggcau...................	247	1	Mm3
......................................................................uggacggagaacugauaagggcaC...................	20	1	Mm3
......................................................................uggacggagGacugauaagggcau...................	1	1	Mm3
......................................................................uggaUggagaacugauaagggcau...................	1	1	Mm3
......................................................................uggacggagCacugauaagggcau...................	2	1	Mm3
......................................................................uggacggagaacugauaagggcau...................	2304	0	Mm3
......................................................................Nggacggagaacugauaagggcau...................	1	1	Mm3
......................................................................uggacggagaacugauaagggcaG...................	22	1	Mm3
......................................................................uggacggagaacugauaaggUcau...................	2	1	Mm3
......................................................................uggacggagaacugauaGgggcau...................	24	1	Mm3
......................................................................uggacggagaGcugauaagggcau...................	5	1	Mm3
......................................................................uggacggagaacugauUagggcau...................	2	1	Mm3
......................................................................uggacNgagaacugauaagggcau...................	1	1	Mm3
......................................................................uggacggagaacugauCagggcau...................	7	1	Mm3
......................................................................uggacggagaCcugauaagggcau...................	1	1	Mm3
......................................................................uAgacggagaacugauaagggcau...................	1	1	Mm3
......................................................................uggacggagaacugauaagggcCu...................	24	1	Mm3
......................................................................uggacggagaacugauaagggAau...................	323	1	Mm3
......................................................................uggacggagaacugauaagggcGu...................	19	1	Mm3
......................................................................uggacggagaacCgauaagggcau...................	1	1	Mm3
......................................................................uggacggagaacugauaagggUau...................	167	1	Mm3
......................................................................uggacggagaacugauaUgggcau...................	6	1	Mm3
......................................................................Aggacggagaacugauaagggcau...................	1	1	Mm3
......................................................................uggacggagaacugauaCgggcau...................	13	1	Mm3
......................................................................uggacggagaacuAauaagggcau...................	1	1	Mm3
......................................................................uggacggagaacugauaagggcauC..................	4	1	Mm3
......................................................................uggacggagaacugauaagggcaua..................	2	0	Mm3
......................................................................uggacggagaacugauaagggcauG..................	1	1	Mm3
......................................................................uggacggagaacugauaagggcaAa..................	2	1	Mm3
......................................................................uggacggagaacugauaagggcUua..................	10	1	Mm3
......................................................................uggacggagaUcugauaagggcaua..................	1	1	Mm3
......................................................................uggacggagaacugauaagggcauU..................	360	1	Mm3
......................................................................uggacggagaacugauaagggcaAag.................	1	1	Mm3
......................................................................uggacggagaacugauaagggcauaU.................	4	1	Mm3
.......................................................................ggacggagaacugauaaU........................	3	1	Mm3
.......................................................................ggacggagaacugauaag........................	18	0	Mm3
.......................................................................ggacggagaacugauaaA........................	12	1	Mm3
.......................................................................ggacggagaCcugauaagg.......................	1	1	Mm3
.......................................................................ggacggagaacugauaagC.......................	1	1	Mm3
```

Star **Mature**

```
cauuugugacaucagaaguaagucuguuucguguccucuucacuuuuccauccugucuguacucgagaacuggacggagaacugauaagggcauagggcagaccacaucaaca
.......................................................................ggacggagaacugauaagA.......................	16	1	Mm3
.......................................................................ggacggCgaacugauaagg.......................	1	1	Mm3
.......................................................................ggacggagaacugauaagg.......................	33	0	Mm3
.......................................................................ggacggagCacugauaagg.......................	1	1	Mm3
.......................................................................Ugacggagaacugauaagg.......................	1	1	Mm3
.......................................................................ggacggagCacugauaaggg......................	2	1	Mm3
.......................................................................ggacggagaacuUauaaggg......................	1	1	Mm3
.......................................................................ggacggagaacugauaaggU......................	1	1	Mm3
.......................................................................Ugacggagaacugauaaggg......................	2	1	Mm3
.......................................................................ggacggagaacugauaaggg......................	61	0	Mm3
.......................................................................ggacgCagaacugauaaggg......................	1	1	Mm3
.......................................................................ggacggagaGcugauaaggg......................	2	1	Mm3
.......................................................................ggacggagaacugauaaggA......................	14	1	Mm3
.......................................................................ggacggagGacugauaagggc.....................	4	1	Mm3
.......................................................................ggacggagaaGugauaagggc.....................	1	1	Mm3
.......................................................................ggacggagaacugauaUgggc.....................	1	1	Mm3
.......................................................................ggacggagaacugauaCgggc.....................	2	1	Mm3
.......................................................................ggacggagaacugaAaagggc.....................	1	1	Mm3
.......................................................................gUacggagaacugauaagggc.....................	2	1	Mm3
.......................................................................ggacggaUaacugauaagggc.....................	1	1	Mm3
.......................................................................ggacCgagaacugauaagggc.....................	1	1	Mm3
.......................................................................Ngacggagaacugauaagggc.....................	1	1	Mm3
.......................................................................ggCcggagaacugauaagggc.....................	1	1	Mm3
.......................................................................ggacggagaaAugauaagggc.....................	1	1	Mm3
.......................................................................ggacggUgaacugauaagggc.....................	1	1	Mm3
.......................................................................ggacggagaacugauaagUgc.....................	1	1	Mm3
.......................................................................ggacggagaacugauaagAgc.....................	2	1	Mm3
.......................................................................Ugacggagaacugauaagggc.....................	12	1	Mm3
.......................................................................ggacggagaaUugauaagggc.....................	1	1	Mm3
.......................................................................ggacggagaacugauGagggc.....................	1	1	Mm3
.......................................................................ggacggagaacugauaagggG.....................	1	1	Mm3
.......................................................................ggaAggagaacugauaagggc.....................	1	1	Mm3
.......................................................................ggacggagaacugauaagggU.....................	9	1	Mm3
.......................................................................ggacggagaacugauaaUggc.....................	1	1	Mm3
.......................................................................ggacggagaacugauaaggUc.....................	1	1	Mm3
.......................................................................ggacggagaCcugauaagggc.....................	1	1	Mm3
.......................................................................ggacggaAaacugauaagggc.....................	1	1	Mm3
.......................................................................ggacggagaacugauaaggAc.....................	2	1	Mm3
.......................................................................ggacggagaacugauaagggc.....................	621	0	Mm3
.......................................................................ggacggagaacugauCagggc.....................	4	1	Mm3
.......................................................................ggacggagaacugauaaAggc.....................	1	1	Mm3
.......................................................................ggacggagaacAgauaagggc.....................	1	1	Mm3
.......................................................................ggacggagCacugauaagggc.....................	6	1	Mm3
.......................................................................ggacggagaacugauaagggA.....................	13	1	Mm3
.......................................................................ggacggagaacugauaGgggc.....................	5	1	Mm3
.......................................................................ggacggagaUcugauaagggca....................	7	1	Mm3
.......................................................................ggGcggagaacugauaagggca....................	1	1	Mm3
.......................................................................ggacggagaacCgauaagggca....................	1	1	Mm3
.......................................................................ggacggagaacugauaagggAa....................	1	1	Mm3
.......................................................................ggacggagaacugauaagggca....................	61	0	Mm3
.......................................................................ggacggagaacugauUagggca....................	1	1	Mm3
.......................................................................ggacggagaacugauaagggcau...................	8	0	Mm3
.......................................................................ggacggagaacugauaUgggcau...................	1	1	Mm3
.......................................................................ggacggaUaacugauaagggcau...................	1	1	Mm3
.......................................................................ggaUggagaacugauaagggcau...................	1	1	Mm3
.......................................................................ggacggagaacugauaagggcauU..................	1	1	Mm3
........................................................................gacggagaacugauaagggc.....................	3	0	Mm3
.........................................................................acggagaacugauaagggc.....................	2	0	Mm3
.........................................................................acggagaacugauaagggca....................	2	0	Mm3

..................................ccucuucacuuuuccauccugu.........................................................	15	0	Fm2
..................................ccucuucacuuuuccaCccugu.........................................................	1	1	Fm2
..................................ccucuucacuuuuccauccugucuguacucgagaac...........................................	11	0	Fm2
..................................ccucuucacuuuuccauccugucuCuacucgagaac...........................................	1	1	Fm2
....................................................................acuggacggagaacugaGaagg.......................	1	1	Fm2
....................................................................acuggacggagaacugauaagg.......................	13	0	Fm2
.....................................................................cuggacggagaacugaua..........................	9	0	Fm2
.....................................................................cuggacggagaacugauU..........................	2	1	Fm2
.....................................................................cuggacggagaacugauaa.........................	17	0	Fm2
.....................................................................cuggacggagaacugaCaag........................	2	1	Fm2
```

```
                                               Star                            Mature
cauuugugacaucagaaguaagucuguuucguguccucuucacuuuuccauccugucuguacucgagaacuggacggagaacugauaagggcauagggcagaccacaucaaca
.....................................................................cuggacggagaacugauaaA........................   3     1   Fm2
.....................................................................cuggacggagaacugauCag........................   1     1   Fm2
.....................................................................cuggacggagaacugauaaU........................   11    1   Fm2
.....................................................................cuggacggagaacugauaag........................   51    0   Fm2
.....................................................................cuggacggGgaacugauaagg.......................   1     1   Fm2
.....................................................................cuggacggagaacugaGaagg.......................   4     1   Fm2
.....................................................................cuggacggagaacugauGagg.......................   2     1   Fm2
.....................................................................cuggacggagCacugauaagg.......................   1     1   Fm2
.....................................................................cuggacggagaacugauaagU.......................   3     1   Fm2
.....................................................................cuggacggagGacugauaagg.......................   1     1   Fm2
.....................................................................cuggacggagaacugauaagg.......................   228   0   Fm2
.....................................................................cuggacggagaacugauaaAg.......................   6     1   Fm2
.....................................................................cuAgacggagaacugauaagg.......................   1     1   Fm2
.....................................................................cuggacggagaacugauaagA.......................   6     1   Fm2
.....................................................................cuggacggagaacugauaagAg......................   1     1   Fm2
.....................................................................Uuggacggagaacugauaaggg......................   1     1   Fm2
.....................................................................cuggacggagaacugauaaAgg......................   7     1   Fm2
.....................................................................cuggacggagaacugauaaggg......................   210   0   Fm2
.....................................................................cuggacggagaacugaGaaggg......................   1     1   Fm2
.....................................................................cuggacggagaacugauCaggg......................   2     1   Fm2
.....................................................................cuggacggagaacugaCaaggg......................   1     1   Fm2
.....................................................................cuggacggagaacugauaaggU......................   45    1   Fm2
.....................................................................cuggacggagaacugauaaggA......................   31    1   Fm2
.....................................................................cuggaUggagaacugauaagggc.....................   2     1   Fm2
.....................................................................cuggacggagCacugauaagggc.....................   1     1   Fm2
.....................................................................cuggacggagaacugaCaagggc.....................   1     1   Fm2
.....................................................................cuggacggagaacugauUagggc.....................   1     1   Fm2
.....................................................................cuggacggagaacugauaagggc.....................   420   0   Fm2
.....................................................................cuggacggagaacugauCagggc.....................   2     1   Fm2
.....................................................................cuggacggagaacCgauaagggc.....................   1     1   Fm2
.....................................................................cuggacggagaacugauaagggU.....................   23    1   Fm2
.....................................................................cuggacggagaacugauaaUggc.....................   1     1   Fm2
.....................................................................cuggacggagaaUugauaagggc.....................   4     1   Fm2
.....................................................................cuggacggagaacugaAaagggc.....................   2     1   Fm2
.....................................................................cuggacggagaacugauaagggG.....................   2     1   Fm2
.....................................................................cuggacggagaacugaGaagggc.....................   2     1   Fm2
.....................................................................cuggacggagaacugauaagggca....................   11    0   Fm2
......................................................................uggacggagaacugauaU.........................   23    1   Fm2
......................................................................uggacggagaacugauGa.........................   3     1   Fm2
......................................................................uggacggagaacugauaC.........................   3     1   Fm2
......................................................................uggacggagaacugaAaa.........................   81    1   Fm2
......................................................................uggacggaUaacugauaa.........................   1     1   Fm2
......................................................................uggacggagaGcugauaa.........................   1     1   Fm2
......................................................................uggacggagaacugauaa.........................   908   0   Fm2
......................................................................uggaUggagaacugauaa.........................   1     1   Fm2
......................................................................uggacggagaacugauUa.........................   9     1   Fm2
......................................................................uggacggagaacugauCa.........................   1     1   Fm2
......................................................................uggacggagaCcugauaa.........................   2     1   Fm2
......................................................................uggacggagaacugauaG.........................   12    1   Fm2
......................................................................uggacggagaacugaGaa.........................   2     1   Fm2
......................................................................uggacNgagaacugauaag........................   2     1   Fm2
......................................................................uggacgUagaacugauaag........................   2     1   Fm2
......................................................................uggacggagaGcugauaag........................   18    1   Fm2
......................................................................Nggacggagaacugauaag........................   3     1   Fm2
......................................................................uggacggUgaacugauaag........................   2     1   Fm2
......................................................................uggacggagCacugauaag........................   2     1   Fm2
......................................................................uggacggagaCcugauaag........................   8     1   Fm2
......................................................................uggacggagaacugauGag........................   9     1   Fm2
......................................................................uUgacggagaacugauaag........................   1     1   Fm2
......................................................................uggacggagaacugauCag........................   15    1   Fm2
......................................................................uggacggagaacuCauaag........................   2     1   Fm2
......................................................................uggGcggagaacugauaag........................   1     1   Fm2
......................................................................uggacggagaacCgauaag........................   1     1   Fm2
......................................................................uggacggagaacugauUag........................   9     1   Fm2
......................................................................uggacggagaaUugauaag........................   2     1   Fm2
......................................................................uggaGggagaacugauaag........................   2     1   Fm2
......................................................................uggaUggagaacugauaag........................   9     1   Fm2
......................................................................uggacggaUaacugauaag........................   1     1   Fm2
......................................................................ugAacggagaacugauaag........................   3     1   Fm2
......................................................................uggUcggagaacugauaag........................   1     1   Fm2
```

Star Mature

```
cauuugugacaucagaaguaagucuguuucguguccucuucacuuuuccauccugucuguacucgagaacuggacggagaacugauaagggcauagggcagaccacaucaaca
......................................................................uAgacggagaacugauaag........................ 6 1 Fm2
......................................................................uggacggagaacugauaag........................ 7644 0 Fm2
......................................................................uggacggGgaacugauaag........................ 3 1 Fm2
......................................................................uggacggCgaacugauaag........................ 1 1 Fm2
......................................................................uggacggagaacugauaaU........................ 1662 1 Fm2
......................................................................uggacggagaacugauaaA........................ 3153 1 Fm2
......................................................................uggacggagaacugauaaC........................ 7 1 Fm2
......................................................................uggacggagaacugauaCg........................ 22 1 Fm2
......................................................................uggacggagaacugaAaag........................ 5 1 Fm2
......................................................................uggacggagaacugauaGg........................ 19 1 Fm2
......................................................................uggacggagaacugaCaag........................ 3 1 Fm2
......................................................................uggacUgagaacugauaag........................ 2 1 Fm2
......................................................................ugCacggagaacugauaag........................ 1 1 Fm2
......................................................................Cggacggagaacugauaag........................ 1 1 Fm2
......................................................................uggacggagUacugauaag........................ 4 1 Fm2
......................................................................uggacggagaacugauaUg........................ 17 1 Fm2
......................................................................uggacggagGacugauaag........................ 9 1 Fm2
......................................................................uggacggagaacAgauaag........................ 1 1 Fm2
......................................................................Aggacggagaacugauaag........................ 1 1 Fm2
......................................................................uggCcggagaacugauaag........................ 1 1 Fm2
......................................................................uggacAgagaacugauaag........................ 1 1 Fm2
......................................................................uggacggagaacugauaaCg....................... 1 1 Fm2
......................................................................uggacCgagaacugauaagg....................... 1 1 Fm2
......................................................................uggacggagaacugauCagg....................... 47 1 Fm2
......................................................................Aggacggagaacugauaagg....................... 16 1 Fm2
......................................................................uggacggaCaacugauaagg....................... 3 1 Fm2
......................................................................uggacggagaacugauaaAg....................... 124 1 Fm2
......................................................................uggacggagaacGgauaagg....................... 1 1 Fm2
......................................................................uAgacggagaacugauaagg....................... 10 1 Fm2
......................................................................uggacggagaacugauaagg....................... 23066 0 Fm2
......................................................................uggacgCagaacugauaagg....................... 2 1 Fm2
......................................................................uggacggagaacCgauaagg....................... 8 1 Fm2
......................................................................uggacggagaacuAauaagg....................... 4 1 Fm2
......................................................................uggacAgagaacugauaagg....................... 5 1 Fm2
......................................................................uggacggagaaUugauaagg....................... 4 1 Fm2
......................................................................uggacNgagaacugauaagg....................... 1 1 Fm2
......................................................................uggacggagaacuCauaagg....................... 2 1 Fm2
......................................................................uggacggCgaacugauaagg....................... 4 1 Fm2
......................................................................uggacggagaacugauGagg....................... 32 1 Fm2
......................................................................uggaAggagaacugauaagg....................... 3 1 Fm2
......................................................................uggacggaAaacugauaagg....................... 1 1 Fm2
......................................................................uggacgUagaacugauaagg....................... 1 1 Fm2
......................................................................Nggacggagaacugauaagg....................... 11 1 Fm2
......................................................................ugAacggagaacugauaagg....................... 6 1 Fm2
......................................................................ugCacggagaacugauaagg....................... 3 1 Fm2
......................................................................uggacggUgaacugauaagg....................... 12 1 Fm2
......................................................................uggGcggagaacugauaagg....................... 3 1 Fm2
......................................................................uggCcggagaacugauaagg....................... 5 1 Fm2
......................................................................uggacggagCacugauaagg....................... 6 1 Fm2
......................................................................uggacggagaacugauaaUg....................... 4 1 Fm2
......................................................................uggacgAagaacugauaagg....................... 3 1 Fm2
......................................................................uggacggagaacugGuaagg....................... 5 1 Fm2
......................................................................uggacggagaacugaGaagg....................... 2 1 Fm2
......................................................................uggacggagaacugauaUgg....................... 70 1 Fm2
......................................................................uggacgNagaacugauaagg....................... 2 1 Fm2
......................................................................uggacggagaacugauaGgg....................... 112 1 Fm2
......................................................................uggacggagUacugauaagg....................... 7 1 Fm2
......................................................................uggacggagaaGugauaagg....................... 1 1 Fm2
......................................................................uggacggagaacugauaagA....................... 1695 1 Fm2
......................................................................uggacggaUaacugauaagg....................... 3 1 Fm2
......................................................................uggacggagaacugauaagU....................... 534 1 Fm2
......................................................................uggacggagaacugUuaagg....................... 4 1 Fm2
......................................................................Cggacggagaacugauaagg....................... 6 1 Fm2
......................................................................uUgacggagaacugauaagg....................... 9 1 Fm2
......................................................................uggacggagaacugauaCgg....................... 93 1 Fm2
......................................................................uggacggagaacugauaagC....................... 3 1 Fm2
......................................................................uggacggagaacugaCaagg....................... 1 1 Fm2
......................................................................uggaGggagaacugauaagg....................... 1 1 Fm2
......................................................................uggacggagaCcugauaagg....................... 68 1 Fm2
......................................................................uggacggagaGcugauaagg....................... 103 1 Fm2
```

```
                                  Star                                  Mature
cauuugugacaucagaaguaagucuguuucguguccucuucacuuuuccauccugucuguacucgagaacuggacggagaacugauaagggcauagggcagaccacaucaaca
......................................................................uggUcggagaacugauaagg.......................    4      1    Fm2
......................................................................uggaUggagaacugauaagg.......................    6      1    Fm2
......................................................................uggacggagaacuUauaagg.......................    5      1    Fm2
......................................................................uggacggagGacugauaagg.......................    23     1    Fm2
......................................................................ugUacggagaacugauaagg.......................    4      1    Fm2
......................................................................uggacUgagaacugauaagg.......................    3      1    Fm2
......................................................................uggacggGgaacugauaagg.......................    7      1    Fm2
......................................................................uggacggagaacugauUagg.......................    28     1    Fm2
......................................................................uggacggagaacugGuaaggg......................    4      1    Fm2
......................................................................uggacggagaacuCauaaggg......................    2      1    Fm2
......................................................................uggCcggagaacugauaaggg......................    4      1    Fm2
......................................................................uggacgNagaacugauaaggg......................    4      1    Fm2
......................................................................uggacAgagaacugauaaggg......................    7      1    Fm2
......................................................................uggacgCagaacugauaaggg......................    3      1    Fm2
......................................................................uggaAggagaacugauaaggg......................    2      1    Fm2
......................................................................uggacggagCacugauaaggg......................    8      1    Fm2
......................................................................uggacggagaacugCuaaggg......................    2      1    Fm2
......................................................................Cggacggagaacugauaaggg......................    8      1    Fm2
......................................................................uggacggagGacugauaaggg......................    27     1    Fm2
......................................................................ugUacggagaacugauaaggg......................    5      1    Fm2
......................................................................uCgacggagaacugauaaggg......................    3      1    Fm2
......................................................................uggaGggagaacugauaaggg......................    5      1    Fm2
......................................................................uggacggagaacugauaNggg......................    1      1    Fm2
......................................................................uggacggagaacugauaagCg......................    2      1    Fm2
......................................................................uggacgUagaacugauaaggg......................    15     1    Fm2
......................................................................uggacggagaacugauaagUg......................    9      1    Fm2
......................................................................uggacggagaGcugauaaggg......................    127    1    Fm2
......................................................................uggacggagaacugauaaggA......................    6724   1    Fm2
......................................................................uggacggaAaacugauaaggg......................    2      1    Fm2
......................................................................uggacggagaacugauaaggC......................    16     1    Fm2
......................................................................ugAacggagaacugauaaggg......................    8      1    Fm2
......................................................................uggacggGgaacugauaaggg......................    4      1    Fm2
......................................................................uggacggagaaUugauaaggg......................    15     1    Fm2
......................................................................uggacggagaacugaAaaggg......................    2      1    Fm2
......................................................................uggacggagaacugauaaggU......................    824    1    Fm2
......................................................................uggacggCgaacugauaaggg......................    2      1    Fm2
......................................................................uggacggagaacugauaaUgg......................    13     1    Fm2
......................................................................uggaUggagaacugauaaggg......................    8      1    Fm2
......................................................................uggacggagaacugauaaCgg......................    3      1    Fm2
......................................................................uggacggagaacugaGaaggg......................    1      1    Fm2
......................................................................uggacggagaacugauaagAg......................    8      1    Fm2
......................................................................uggacggagaacugaCaaggg......................    6      1    Fm2
......................................................................uggacggagaacugauUaggg......................    56     1    Fm2
......................................................................uggacggagaacugauaaggg......................    35348  0    Fm2
......................................................................uUgacggagaacugauaaggg......................    10     1    Fm2
......................................................................Nggacggagaacugauaaggg......................    16     1    Fm2
......................................................................uggacggagaCcugauaaggg......................    67     1    Fm2
......................................................................uggacggUgaacugauaaggg......................    21     1    Fm2
......................................................................uggacggagaacAgauaaggg......................    3      1    Fm2
......................................................................uggacggagaacugauGaggg......................    52     1    Fm2
......................................................................uggacggagaaGugauaaggg......................    1      1    Fm2
......................................................................uggacUgagaacugauaaggg......................    9      1    Fm2
......................................................................uggacggaUaacugauaaggg......................    5      1    Fm2
......................................................................uggacggagaacugauCaggg......................    75     1    Fm2
......................................................................uggacggagaacugauaUggg......................    112    1    Fm2
......................................................................Gggacggagaacugauaaggg......................    4      1    Fm2
......................................................................uggacNgagaacugauaaggg......................    4      1    Fm2
......................................................................uAgacggagaacugauaaggg......................    20     1    Fm2
......................................................................uggacggagaacCgauaaggg......................    7      1    Fm2
......................................................................ugCacggagaacugauaaggg......................    4      1    Fm2
......................................................................Aggacggagaacugauaaggg......................    22     1    Fm2
......................................................................uggacggagaaAugauaaggg......................    1      1    Fm2
......................................................................uggacCgagaacugauaaggg......................    1      1    Fm2
......................................................................uggacggagaacugauaaAgg......................    3      1    Fm2
......................................................................uggacggagaacugUuaaggg......................    6      1    Fm2
......................................................................uggacggagUacugauaaggg......................    16     1    Fm2
......................................................................uggacggagaacuUauaaggg......................    2      1    Fm2
......................................................................uggacggagaacugauaCggg......................    130    1    Fm2
......................................................................uggacgAagaacugauaaggg......................    2      1    Fm2
......................................................................uggacggagaacugauaGggg......................    193    1    Fm2
```

Star **Mature**

```
cauuugugacaucagaaguaagucuguuucguguccucuucacuuuuccauccugucuguacucgagaacuggacggagaacugauaagggcauagggcagaccacaucaaca
......................................................................uggacggagaacuAauaaggg......................    3       1    Fm2
......................................................................uggUcggagaacugauaaggg......................    4       1    Fm2
......................................................................uggGcggagaacugauaaggg......................    5       1    Fm2
......................................................................uggacUgagaacugauaagggc.....................    58      1    Fm2
......................................................................uggacggagUacugauaagggc.....................    107     1    Fm2
......................................................................Aggacggagaacugauaagggc.....................    62      1    Fm2
......................................................................uggacggagaacugauaGgggc.....................    6080    1    Fm2
......................................................................uggacggagaCcugauaagggc.....................    221     1    Fm2
......................................................................uggacggagaacugauCagggc.....................    583     1    Fm2
......................................................................uggacggagaGcugauaagggc.....................    478     1    Fm2
......................................................................uggacggagaacGgauaagggc.....................    7       1    Fm2
......................................................................uggacggagaacugauaagggU.....................    2043    1    Fm2
......................................................................uggacggagaacugauaagCgc.....................    17      1    Fm2
......................................................................uggacggagaacugaAaagggc.....................    7       1    Fm2
......................................................................uNgacggagaacugauaagggc.....................    3       1    Fm2
......................................................................ugAacggagaacugauaagggc.....................    70      1    Fm2
......................................................................uggacggagaacugauaaUggc.....................    66      1    Fm2
......................................................................uggacggagaacugauaaggCc.....................    31      1    Fm2
......................................................................uggacggagaacAgauaagggc.....................    7       1    Fm2
......................................................................uggacggagaaGugauaagggc.....................    6       1    Fm2
......................................................................uggacggGgaacugauaagggc.....................    83      1    Fm2
......................................................................uggacggUgaacugauaagggc.....................    161     1    Fm2
......................................................................uggacggaAaacugauaagggc.....................    15      1    Fm2
......................................................................uggacggagaacugauaCgggc.....................    1038    1    Fm2
......................................................................uggacggagaacuUauaagggc.....................    30      1    Fm2
......................................................................uggacggagaacugauaagAgc.....................    38      1    Fm2
......................................................................uggacgNagaacugauaagggc.....................    16      1    Fm2
......................................................................uUgacggagaacugauaagggc.....................    77      1    Fm2
......................................................................ugCacggagaacugauaagggc.....................    28      1    Fm2
......................................................................ugNacggagaacugauaagggc.....................    2       1    Fm2
......................................................................uggacggagCacugauaagggc.....................    56      1    Fm2
......................................................................uggacggagaacugauaUgggc.....................    904     1    Fm2
......................................................................Gggacggagaacugauaagggc.....................    7       1    Fm2
......................................................................uggCcggagaacugauaagggc.....................    44      1    Fm2
......................................................................uggacggagaacugauGagggc.....................    490     1    Fm2
......................................................................uggGcggagaacugauaagggc.....................    55      1    Fm2
......................................................................uggacggagaacugaGaagggc.....................    9       1    Fm2
......................................................................uggacCgagaacugauaagggc.....................    10      1    Fm2
......................................................................uggacggaUaacugauaagggc.....................    55      1    Fm2
......................................................................uggacggagaacuCauaagggc.....................    16      1    Fm2
......................................................................uggacggagaacugauaaCggc.....................    8       1    Fm2
......................................................................uggacggagaacugauaaggUc.....................    78      1    Fm2
......................................................................uggacggagaacugauaagggG.....................    452     1    Fm2
......................................................................uggacggagaacugauaaAggc.....................    40      1    Fm2
......................................................................uggacggagaacugUuaagggc.....................    40      1    Fm2
......................................................................uggacggCgaacugauaagggc.....................    50      1    Fm2
......................................................................uggacggagaacugauaagggA.....................    13052   1    Fm2
......................................................................Cggacggagaacugauaagggc.....................    37      1    Fm2
......................................................................uggaUggagaacugauaagggc.....................    84      1    Fm2
......................................................................uggacggagaacugCuaagggc.....................    7       1    Fm2
......................................................................uggacggagaacCgauaagggc.....................    29      1    Fm2
......................................................................uggacggaCaacugauaagggc.....................    11      1    Fm2
......................................................................uggacgCagaacugauaagggc.....................    27      1    Fm2
......................................................................uggUcggagaacugauaagggc.....................    43      1    Fm2
......................................................................uggacggagaacugaCaagggc.....................    22      1    Fm2
......................................................................uCgacggagaacugauaagggc.....................    38      1    Fm2
......................................................................uggaGggagaacugauaagggc.....................    13      1    Fm2
......................................................................uggacggagaacugauaagggc.....................    272144  0    Fm2
......................................................................uggacAgagaacugauaagggc.....................    38      1    Fm2
......................................................................uggacggagaacugGuaagggc.....................    26      1    Fm2
......................................................................uggaAggagaacugauaagggc.....................    32      1    Fm2
......................................................................uAgacggagaacugauaagggc.....................    133     1    Fm2
......................................................................uggacggagaacugauaaggAc.....................    83      1    Fm2
......................................................................uggacgAagaacugauaagggc.....................    32      1    Fm2
......................................................................Nggacggagaacugauaagggc.....................    143     1    Fm2
......................................................................uggacgUagaacugauaagggc.....................    60      1    Fm2
......................................................................uggacggagaacuAauaagggc.....................    64      1    Fm2
......................................................................uggacggagaacugauUagggc.....................    345     1    Fm2
......................................................................uggacggagaaUugauaagggc.....................    70      1    Fm2
......................................................................uggacggagaaAugauaagggc.....................    5       1    Fm2
```

```
                                                  Star                                  Mature
cauuugugacaucagaaguaagucuguuucguguccucuucacuuuuccauccugucuguacucgagaacuggacggagaacugauaagggcauagggcagaccacaucaaca
......................................................................uggacggagaacugauaagUgc.....................    78      1    Fm2
......................................................................ugUacggagaacugauaagggc.....................    54      1    Fm2
......................................................................uggacggagGacugauaagggc.....................    1032    1    Fm2
......................................................................uggacNgagaacugauaagggc.....................    34      1    Fm2
......................................................................uggacggagaacugauaaggAca....................    6       1    Fm2
......................................................................uggacggagaacugauaagggca....................    25517   0    Fm2
......................................................................uggacggagaacuAauaagggca....................    4       1    Fm2
......................................................................uggacggaAaacugauaagggca....................    1       1    Fm2
......................................................................uggacggagaacugaGaagggca....................    1       1    Fm2
......................................................................Cggacggagaacugauaagggca....................    6       1    Fm2
......................................................................uggacggagaacugauaaUggca....................    5       1    Fm2
......................................................................uggacggagaacugauaaggCca....................    3       1    Fm2
......................................................................Nggacggagaacugauaagggca....................    17      1    Fm2
......................................................................uggGcggagaacugauaagggca....................    6       1    Fm2
......................................................................Gggacggagaacugauaagggca....................    1       1    Fm2
......................................................................uggaGggagaacugauaagggca....................    3       1    Fm2
......................................................................uCgacggagaacugauaagggca....................    3       1    Fm2
......................................................................uggacggagaacugauaagggcC....................    179     1    Fm2
......................................................................uggacggagaacugauaagUgca....................    8       1    Fm2
......................................................................uggacggagaacugauaaCggca....................    1       1    Fm2
......................................................................uggacggagaacCgauaagggca....................    3       1    Fm2
......................................................................uggacggagaacugauaagAgca....................    4       1    Fm2
......................................................................uggacggagUacugauaagggca....................    12      1    Fm2
......................................................................uggacggagaacugGuaagggca....................    2       1    Fm2
......................................................................uggacgAagaacugauaagggca....................    3       1    Fm2
......................................................................uggaAggagaacugauaagggca....................    2       1    Fm2
......................................................................uggacggagaacugauaaAggca....................    4       1    Fm2
......................................................................uggacggagaacugauaagggUa....................    99      1    Fm2
......................................................................uggacggagaacugauGagggca....................    65      1    Fm2
......................................................................uggacCgagaacugauaagggca....................    1       1    Fm2
......................................................................uggacggagaacugauaGgggca....................    273     1    Fm2
......................................................................uggacggagaacuUauaagggca....................    1       1    Fm2
......................................................................uggCcggagaacugauaagggca....................    3       1    Fm2
......................................................................uggacggUgaacugauaagggca....................    8       1    Fm2
......................................................................uggacggagaacugaAaagggca....................    2       1    Fm2
......................................................................uggacggagaacugauaUgggca....................    89      1    Fm2
......................................................................uggacggagaacAgauaagggca....................    1       1    Fm2
......................................................................uggacggagaacuCauaagggca....................    4       1    Fm2
......................................................................uggacggaCaacugauaagggca....................    1       1    Fm2
......................................................................uggUcggagaacugauaagggca....................    5       1    Fm2
......................................................................Aggacggagaacugauaagggca....................    2       1    Fm2
......................................................................uggacggagaacugauaagggcG....................    874     1    Fm2
......................................................................uggacAgagaacugauaagggca....................    4       1    Fm2
......................................................................uAgacggagaacugauaagggca....................    20      1    Fm2
......................................................................uggacggagaCcugauaagggca....................    6       1    Fm2
......................................................................uggacggagCacugauaagggca....................    7       1    Fm2
......................................................................uggacggagaacugauaagggAa....................    701     1    Fm2
......................................................................ugAacggagaacugauaagggca....................    4       1    Fm2
......................................................................ugUacggagaacugauaagggca....................    5       1    Fm2
......................................................................uggacggCgaacugauaagggca....................    1       1    Fm2
......................................................................uggacUgagaacugauaagggca....................    8       1    Fm2
......................................................................uggacggGgaacugauaagggca....................    7       1    Fm2
......................................................................uggacggagaacugauUagggca....................    32      1    Fm2
......................................................................uggacggagaacugauaaggUca....................    9       1    Fm2
......................................................................uggacggagaacugauaCgggca....................    110     1    Fm2
......................................................................uggacggagaUcugauaagggca....................    768     1    Fm2
......................................................................uUgacggagaacugauaagggca....................    7       1    Fm2
......................................................................uggacggagaacugUuaagggca....................    5       1    Fm2
......................................................................uggacggagaaUugauaagggca....................    5       1    Fm2
......................................................................uggacggagGacugauaagggca....................    29      1    Fm2
......................................................................uggacNgagaacugauaagggca....................    3       1    Fm2
......................................................................uggacggagaGcugauaagggca....................    39      1    Fm2
......................................................................uggacggagaacugauCagggca....................    49      1    Fm2
......................................................................uggacgUagaacugauaagggca....................    7       1    Fm2
......................................................................uggaUggagaacugauaagggca....................    9       1    Fm2
......................................................................uggacggaUaacugauaagggca....................    4       1    Fm2
......................................................................uggacUgagaacugauaagggcau...................    1       1    Fm2
......................................................................uggacggagaacugauUagggcau...................    2       1    Fm2
......................................................................uggacggagaacugauaagggcaA...................    67      1    Fm2
......................................................................uggacggagaacugauaagggUau...................    267     1    Fm2
```

Star Mature

```
cauuugugacaucagaaguaagucuguuucguguccucuucacuuuuccauccugucuguacucgagaacuggacggagaacugauaagggcauagggcagaccacaucaaca
......................................................................uggacgUagaacugauaagggcau...................   2     1   Fm2
......................................................................uggacggagaacugauaagggcau...................   1743  0   Fm2
......................................................................uCgacggagaacugauaagggcau...................   1     1   Fm2
......................................................................uggacggagaUcugauaagggcau...................   123   1   Fm2
......................................................................uUgacggagaacugauaagggcau...................   1     1   Fm2
......................................................................uggacggagaacugauaaAggcau...................   1     1   Fm2
......................................................................uggacggagaacugauaGgggcau...................   35    1   Fm2
......................................................................uggacggagaacugauaagggcaG...................   4     1   Fm2
......................................................................uggGcggagaacugauaagggcau...................   1     1   Fm2
......................................................................uggacggagaacugauaCgggcau...................   6     1   Fm2
......................................................................uggacggagaacugauaagggcGu...................   5     1   Fm2
......................................................................ugCacggagaacugauaagggcau...................   1     1   Fm2
......................................................................uggacggagaacugauGagggcau...................   3     1   Fm2
......................................................................uggacggagaGcugauaagggcau...................   2     1   Fm2
......................................................................uggacggagaacugauaUgggcau...................   8     1   Fm2
......................................................................uggacggagGacugauaagggcau...................   1     1   Fm2
......................................................................uggacggagaacugauaagggcCu...................   13    1   Fm2
......................................................................uggacggagaacugauaaCggcau...................   1     1   Fm2
......................................................................uAgacggagaacugauaagggcau...................   1     1   Fm2
......................................................................uggacggagaacugauaagUgcau...................   1     1   Fm2
......................................................................uggacggagaacugauaagggAau...................   602   1   Fm2
......................................................................uggacggagaCcugauaagggcau...................   2     1   Fm2
......................................................................Nggacggagaacugauaagggcau...................   2     1   Fm2
......................................................................uggacggagaacuCauaagggcau...................   1     1   Fm2
......................................................................uggacggagaacugauCagggcau...................   5     1   Fm2
......................................................................uggacggagaacugauaagggcUua..................   3     1   Fm2
......................................................................uggacggagaacugauaagggcauU..................   383   1   Fm2
......................................................................uggacggagaacugauaagggcauC..................   12    1   Fm2
......................................................................uggacggagaacugauaagggcauUg.................   1     1   Fm2
......................................................................uggacggagaacugauaagggcauaU.................   14    1   Fm2
.......................................................................ggacggagaacugauaaU........................   5     1   Fm2
.......................................................................ggacggagaacugauaag........................   3     0   Fm2
.......................................................................ggacggagaacugauaaA........................   2     1   Fm2
.......................................................................ggacggagaacugauaagU.......................   1     1   Fm2
.......................................................................ggacggagaacGgauaagg.......................   1     1   Fm2
.......................................................................ggacggagGacugauaagg.......................   1     1   Fm2
.......................................................................ggacggagaacugauaGgg.......................   1     1   Fm2
.......................................................................Ugacggagaacugauaagg.......................   2     1   Fm2
.......................................................................ggacggagaacugauaagg.......................   30    0   Fm2
.......................................................................ggacggagaacugauaagA.......................   2     1   Fm2
.......................................................................Ugacggagaacugauaaggg......................   5     1   Fm2
.......................................................................ggacggagaacugauaagAg......................   1     1   Fm2
.......................................................................ggacggagaacugauaagUg......................   1     1   Fm2
.......................................................................ggacggagaacugauaaggA......................   8     1   Fm2
.......................................................................ggacggagaacugauaaggg......................   39    0   Fm2
.......................................................................ggacgUagaacugauaagggc.....................   1     1   Fm2
.......................................................................ggacggagaacugauaagCgc.....................   1     1   Fm2
.......................................................................ggacggagaacuUauaagggc.....................   1     1   Fm2
.......................................................................ggacggagaacuAauaagggc.....................   2     1   Fm2
.......................................................................ggacggagaacugauaaggAc.....................   3     1   Fm2
.......................................................................ggacggagaacugauaGgggc.....................   6     1   Fm2
.......................................................................ggacggNgaacugauaagggc.....................   1     1   Fm2
.......................................................................ggacggagaacugauaagggc.....................   191   0   Fm2
.......................................................................ggacggagaaAugauaagggc.....................   1     1   Fm2
.......................................................................ggacggagaaUugauaagggc.....................   2     1   Fm2
.......................................................................ggacggagUacugauaagggc.....................   1     1   Fm2
.......................................................................ggacggagaacugauaagggA.....................   22    1   Fm2
.......................................................................ggacggagCacugauaagggc.....................   3     1   Fm2
.......................................................................ggacggagaacugauaaggCc.....................   1     1   Fm2
.......................................................................Agacggagaacugauaagggc.....................   1     1   Fm2
.......................................................................ggacggaAaacugauaagggc.....................   1     1   Fm2
.......................................................................Ugacggagaacugauaagggc.....................   13    1   Fm2
.......................................................................ggacggUgaacugauaagggc.....................   1     1   Fm2
.......................................................................ggacggagaacugauaagggU.....................   1     1   Fm2
.......................................................................ggacggagaUcugauaagggca....................   5     1   Fm2
.......................................................................ggacggagaaAugauaagggca....................   1     1   Fm2
.......................................................................ggacggagaacugauaagggca....................   31    0   Fm2
.......................................................................ggacggagaacugauaagggcG....................   1     1   Fm2
.......................................................................ggacggagaacugauaagggcau...................   2     0   Fm2
.......................................................................ggacggagaGcugauaagggcau...................   1     1   Fm2
```

```
                                  Star                                  Mature
cauuugugacaucagaaguaagucuguuucguguccucuucacuuuuccauccugucuguacucgagaacuggacggagaacugauaagggcauagggcagaccacaucaaca
........................................................................gacggagaacugauaagg.......................    1    0    Fm2
..........................................................................Uggagaacugauaagggc.....................    1    1    Fm2
..........................................................................cggagaacugauaagggc.....................    5    0    Fm2

..................................ccucuucacuuuuccauccugu.........................................................    14   0    Fm1
..................................ccucuucacuuuuccauccugG.........................................................    1    1    Fm1
..................................ccucuucacuuuuccauccuguc........................................................    2    0    Fm1
..................................ccucuucacuuuuccauccuguU........................................................    3    1    Fm1
..................................ccucuucacuuuuccauccugucugCacuc.................................................    1    1    Fm1
..................................ccucuucacuuuuccauccugucuguacuc.................................................    6    0    Fm1
..................................ccucuucacuuuuccauccugucuguacucgagaa............................................    1    0    Fm1
..................................ccucuucacuuuuccauccugucuguacucgagaacG..........................................    1    1    Fm1
.............................................uuccauccugucuguacucgaga.............................................    2    0    Fm1
...............................................................cgagaacuggacggagaacugauaagggc.....................    6    0    Fm1
...................................................................aUcuggacggagaacugauaagggc.....................    4    1    Fm1
....................................................................acuggacggagaacugauaaU........................    4    1    Fm1
....................................................................acuggacggagaacugauaagg.......................    2    0    Fm1
....................................................................acuggacggagaacugauaaggA......................    2    1    Fm1
....................................................................acuggacggagaacugauaagggc.....................    3    0    Fm1
.....................................................................cuggacggagaacugaua..........................    2    0    Fm1
.....................................................................cuggacggagGacugauaa.........................    1    1    Fm1
.....................................................................cuggacggagaacugauaa.........................    6    0    Fm1
.....................................................................cuggacggagaacugauCa.........................    1    1    Fm1
.....................................................................cuggacggagaacugaAaa.........................    5    1    Fm1
.....................................................................cuggacggagaacugauaag........................    36   0    Fm1
.....................................................................cAggacggagaacugauaag........................    1    1    Fm1
.....................................................................cuggacggagaacugauaaA........................    14   1    Fm1
.....................................................................cuggacggagaacugauaaU........................    3    1    Fm1
.....................................................................cuggacggaUaacugauaagg.......................    1    1    Fm1
.....................................................................cuggacggagaacugauaagg.......................    165  0    Fm1
.....................................................................cuggacggagaacugauCagg.......................    1    1    Fm1
.....................................................................Nuggacggagaacugauaagg.......................    1    1    Fm1
.....................................................................cuggaNggagaacugauaagg.......................    1    1    Fm1
.....................................................................cuggacggagaacugauaagA.......................    18   1    Fm1
.....................................................................cuggacggagaacugauaagU.......................    1    1    Fm1
.....................................................................cuggacggagaacAgauaagg.......................    1    1    Fm1
.....................................................................cuggacggagaacugauaaggg......................    169  0    Fm1
.....................................................................cuggacggagUacugauaaggg......................    1    1    Fm1
.....................................................................cuggacggagaacugauGaggg......................    2    1    Fm1
.....................................................................cuggacggagaacugauaaggC......................    5    1    Fm1
.....................................................................cuggacggagaacugaGaaggg......................    1    1    Fm1
.....................................................................cuggacggagCacugauaaggg......................    1    1    Fm1
.....................................................................cuggacggagaacugauaaggA......................    15   1    Fm1
.....................................................................cuggacUgagaacugauaaggg......................    1    1    Fm1
.....................................................................cuggacggagaacugauaaggU......................    32   1    Fm1
.....................................................................cuggacggagaacugauUagggc.....................    1    1    Fm1
.....................................................................cuggacAgagaacugauaagggc.....................    1    1    Fm1
.....................................................................cuggacggagaacugaAaagggc.....................    1    1    Fm1
.....................................................................cuggacggGgaacugauaagggc.....................    2    1    Fm1
.....................................................................cuggacggagaacugauaagggc.....................    422  0    Fm1
.....................................................................cuggacggagaacugauaagggU.....................    4    1    Fm1
.....................................................................cuggacUgagaacugauaagggc.....................    1    1    Fm1
.....................................................................Nuggacggagaacugauaagggc.....................    1    1    Fm1
.....................................................................cuggacggUgaacugauaagggc.....................    1    1    Fm1
.....................................................................cuggacggagaacugauCagggc.....................    1    1    Fm1
.....................................................................cuggacggagGacugauaagggc.....................    4    1    Fm1
.....................................................................cuggacggagaaUugauaagggc.....................    1    1    Fm1
.....................................................................cuggacggagaacugauaGgggc.....................    9    1    Fm1
.....................................................................cuggacggagaacugaCaagggc.....................    4    1    Fm1
.....................................................................cuggacggagUacugauaagggc.....................    2    1    Fm1
.....................................................................cuggacggagaacugauaagggA.....................    37   1    Fm1
.....................................................................Uuggacggagaacugauaagggc.....................    1    1    Fm1
.....................................................................cuggacggagaCcugauaagggc.....................    1    1    Fm1
.....................................................................cuggacggagaacugauGagggc.....................    1    1    Fm1
.....................................................................cGggacggagaacugauaagggc.....................    1    1    Fm1
.....................................................................cuggacggagaacugauaagggAa....................    2    1    Fm1
.....................................................................cuggacggagaacugauaagggcG....................    1    1    Fm1
.....................................................................cuggacggagaacugauaagggca....................    38   0    Fm1
.....................................................................cuggacggagaCcugauaagggca....................    1    1    Fm1
.....................................................................cuggacggagaacugauaagggcau...................    3    0    Fm1
```

Star Mature

```
cauuugugacaucagaaguaagucuguuucguguccucuucacuuuuccauccugucuguacucgagaacuggacggagaacugauaagggcauagggcagaccacaucaaca
......................................................................uggacggagaacugaAaa.........................   215    1   Fm1
......................................................................uggGcggagaacugauaa.........................   1      1   Fm1
......................................................................uggacggagUacugauaa.........................   1      1   Fm1
......................................................................uggacggagaacugauCa.........................   3      1   Fm1
......................................................................uAgacggagaacugauaa.........................   2      1   Fm1
......................................................................uggacggagaCcugauaa.........................   1      1   Fm1
......................................................................uggacggagaacugUuaa.........................   1      1   Fm1
......................................................................uggacggUgaacugauaa.........................   2      1   Fm1
......................................................................uggacggagaGcugauaa.........................   10     1   Fm1
......................................................................uggacggagaacugauaG.........................   14     1   Fm1
......................................................................uggacggagaacugauGa.........................   1      1   Fm1
......................................................................uggacggagaacugauaC.........................   15     1   Fm1
......................................................................Nggacggagaacugauaa.........................   1      1   Fm1
......................................................................uggacggagaacugauUa.........................   9      1   Fm1
......................................................................ugAacggagaacugauaa.........................   1      1   Fm1
......................................................................uggacggagaacugauaa.........................   2454   0   Fm1
......................................................................uggacggagaacugCuaa.........................   1      1   Fm1
......................................................................uggacggagaacugauaU.........................   88     1   Fm1
......................................................................uggacggagaacugaGaa.........................   6      1   Fm1
......................................................................uggacggagaacugauaUg........................   63     1   Fm1
......................................................................uggacggagaacugUuaag........................   6      1   Fm1
......................................................................uNgacggagaacugauaag........................   1      1   Fm1
......................................................................uggacggagaacuUauaag........................   3      1   Fm1
......................................................................uggacggagaacugauaCg........................   70     1   Fm1
......................................................................ugCacggagaacugauaag........................   2      1   Fm1
......................................................................uggacggagaaUugauaag........................   8      1   Fm1
......................................................................uggacggagaacugauCag........................   39     1   Fm1
......................................................................uUgacggagaacugauaag........................   1      1   Fm1
......................................................................uggCcggagaacugauaag........................   1      1   Fm1
......................................................................uggacggagaacugauaaA........................   6927   1   Fm1
......................................................................uggacggUgaacugauaag........................   4      1   Fm1
......................................................................uggacggagaacugauaaU........................   4300   1   Fm1
......................................................................uggacggagaacugauUag........................   24     1   Fm1
......................................................................uggaUggagaacugauaag........................   4      1   Fm1
......................................................................uggaAggagaacugauaag........................   1      1   Fm1
......................................................................uggacggagaacuCauaag........................   2      1   Fm1
......................................................................uggacggagCacugauaag........................   2      1   Fm1
......................................................................uggacCgagaacugauaag........................   1      1   Fm1
......................................................................ugAacggagaacugauaag........................   5      1   Fm1
......................................................................uggacggagaacugauaag........................   17744  0   Fm1
......................................................................uggacggagaacuAauaag........................   2      1   Fm1
......................................................................uggacggagaacAgauaag........................   1      1   Fm1
......................................................................uggacggagaacugaAaag........................   19     1   Fm1
......................................................................uggacggaAaacugauaag........................   3      1   Fm1
......................................................................uggacgAagaacugauaag........................   3      1   Fm1
......................................................................uggacNgagaacugauaag........................   4      1   Fm1
......................................................................uggacAgagaacugauaag........................   5      1   Fm1
......................................................................Aggacggagaacugauaag........................   3      1   Fm1
......................................................................uggacggagaacugauGag........................   21     1   Fm1
......................................................................uggacggagaacCgauaag........................   4      1   Fm1
......................................................................uggacggagaacugCuaag........................   1      1   Fm1
......................................................................uggacggagUacugauaag........................   3      1   Fm1
......................................................................uggacgUagaacugauaag........................   5      1   Fm1
......................................................................ugUacggagaacugauaag........................   1      1   Fm1
......................................................................uggacggagaaAugauaag........................   1      1   Fm1
......................................................................uggacggagaacugauaaC........................   30     1   Fm1
......................................................................uggacgCagaacugauaag........................   1      1   Fm1
......................................................................uggacggagGacugauaag........................   9      1   Fm1
......................................................................uggacggGgaacugauaag........................   4      1   Fm1
......................................................................uggacUgagaacugauaag........................   5      1   Fm1
......................................................................Nggacggagaacugauaag........................   10     1   Fm1
......................................................................uAgacggagaacugauaag........................   10     1   Fm1
......................................................................uggacggagaCcugauaag........................   24     1   Fm1
......................................................................uggaGggagaacugauaag........................   1      1   Fm1
......................................................................uggacggagaacugauaGg........................   72     1   Fm1
......................................................................uggacggagaacGgauaag........................   1      1   Fm1
......................................................................uggacggagaGcugauaag........................   31     1   Fm1
......................................................................uggacggaUaacugauaag........................   4      1   Fm1
......................................................................Cggacggagaacugauaag........................   2      1   Fm1
......................................................................uggacggagaacugaCaag........................   2      1   Fm1
```

```
                                   Star                                   Mature
cauuugugacaucagaaguaagucuguuucguguccucuucacuuuuccauccugucuguacucgagaacuggacggagaacugauaagggcauagggcagaccacaucaaca
......................................................................uggGcggagaacugauaag........................ 8 1 Fm1
......................................................................uggUcggagaacugauaag........................ 5 1 Fm1
......................................................................uggaAggagaacugauaagg....................... 1 1 Fm1
......................................................................uggacggaCaacugauaagg....................... 1 1 Fm1
......................................................................Aggacggagaacugauaagg....................... 17 1 Fm1
......................................................................uggacggagaacAgauaagg....................... 2 1 Fm1
......................................................................uggacggagaacugauaagC....................... 31 1 Fm1
......................................................................Nggacggagaacugauaagg....................... 17 1 Fm1
......................................................................uggacggagaacuUauaagg....................... 1 1 Fm1
......................................................................uggacggagaacugauaUgg....................... 165 1 Fm1
......................................................................uggacgNagaacugauaagg....................... 2 1 Fm1
......................................................................uggacggagaacugauaaAg....................... 266 1 Fm1
......................................................................uggacggagaacugauaagg....................... 46362 0 Fm1
......................................................................Cggacggagaacugauaagg....................... 9 1 Fm1
......................................................................uggacCgagaacugauaagg....................... 2 1 Fm1
......................................................................uNgacggagaacugauaagg....................... 2 1 Fm1
......................................................................uggacggagaacugaCaagg....................... 2 1 Fm1
......................................................................uggacggagaacugCuaagg....................... 2 1 Fm1
......................................................................uggacggagaCcugauaagg....................... 71 1 Fm1
......................................................................ugCacggagaacugauaagg....................... 1 1 Fm1
......................................................................uAgacggagaacugauaagg....................... 27 1 Fm1
......................................................................uggacgUagaacugauaagg....................... 8 1 Fm1
......................................................................uggacggagaaUugauaagg....................... 8 1 Fm1
......................................................................uggacgCagaacugauaagg....................... 2 1 Fm1
......................................................................uggacggCgaacugauaagg....................... 4 1 Fm1
......................................................................uggacggUgaacugauaagg....................... 18 1 Fm1
......................................................................ugAacggagaacugauaagg....................... 9 1 Fm1
......................................................................uggacggaAaacugauaagg....................... 2 1 Fm1
......................................................................uggacggagaacugauaagU....................... 1110 1 Fm1
......................................................................uUgacggagaacugauaagg....................... 8 1 Fm1
......................................................................uggacggagaacugauaaUg....................... 3 1 Fm1
......................................................................uggacggagaacugauaCgg....................... 177 1 Fm1
......................................................................ugUacggagaacugauaagg....................... 5 1 Fm1
......................................................................uggacUgagaacugauaagg....................... 14 1 Fm1
......................................................................uggacggagaacuAauaagg....................... 9 1 Fm1
......................................................................uggacggagaacugaAaagg....................... 2 1 Fm1
......................................................................uggUcggagaacugauaagg....................... 8 1 Fm1
......................................................................uggacggagaaGugauaagg....................... 1 1 Fm1
......................................................................uggGcggagaacugauaagg....................... 16 1 Fm1
......................................................................uggacgAagaacugauaagg....................... 4 1 Fm1
......................................................................uggacggagaacugauaaCg....................... 4 1 Fm1
......................................................................uggacggagaacugGuaagg....................... 7 1 Fm1
......................................................................uggaUggagaacugauaagg....................... 9 1 Fm1
......................................................................uggacggagaacugauaagA....................... 4462 1 Fm1
......................................................................uggacggagUacugauaagg....................... 17 1 Fm1
......................................................................uggacggagaacugauGagg....................... 63 1 Fm1
......................................................................uggacggagGacugauaagg....................... 51 1 Fm1
......................................................................uggacggagaGcugauaagg....................... 149 1 Fm1
......................................................................uggacggagaacCgauaagg....................... 3 1 Fm1
......................................................................uggacggagaacugauaGgg....................... 199 1 Fm1
......................................................................uggacggagaacugauCagg....................... 122 1 Fm1
......................................................................uggacNgagaacugauaagg....................... 2 1 Fm1
......................................................................uggCcggagaacugauaagg....................... 9 1 Fm1
......................................................................uggacggagaacugauUagg....................... 70 1 Fm1
......................................................................uggacggGgaacugauaagg....................... 8 1 Fm1
......................................................................uCgacggagaacugauaagg....................... 4 1 Fm1
......................................................................uggacggagaacugUuaagg....................... 9 1 Fm1
......................................................................uggacggagaacuCauaagg....................... 4 1 Fm1
......................................................................uggacggagCacugauaagg....................... 6 1 Fm1
......................................................................uggacAgagaacugauaagg....................... 5 1 Fm1
......................................................................uggacggaUaacugauaagg....................... 8 1 Fm1
......................................................................uCgacggagaacugauaaggg...................... 5 1 Fm1
......................................................................uggacggagaacugauaaggg...................... 58344 0 Fm1
......................................................................uggacggagaacuAauaaggg...................... 13 1 Fm1
......................................................................uggacggagaacugGuaaggg...................... 13 1 Fm1
......................................................................uggacggagaacugauaagCg...................... 5 1 Fm1
......................................................................uggacggagaacAgauaaggg...................... 4 1 Fm1
......................................................................uggacggagaacuUauaaggg...................... 4 1 Fm1
......................................................................uggaUggagaacugauaaggg...................... 13 1 Fm1
......................................................................uggacggagaacugaCaaggg...................... 2 1 Fm1
```

```
                                  Star                                  Mature
cauuugugacaucagaaguaagucuguuucguguccucuucacuuuuccauccuguc uguacucgagaacuggacggagaacugauaagggcauagggcagaccacaucaaca
......................................................................uggacggagaacugauGaggg......................  97     1  Fm1
......................................................................uggaGggagaacugauaaggg......................  3      1  Fm1
......................................................................uggacggagaacugauaaggA......................  11343  1  Fm1
......................................................................uggacgNagaacugauaaggg......................  5      1  Fm1
......................................................................uggacggUgaacugauaaggg......................  20     1  Fm1
......................................................................uggacggaUaacugauaaggg......................  14     1  Fm1
......................................................................uggacggagaacugauaGggg......................  524    1  Fm1
......................................................................uggUcggagaacugauaaggg......................  6      1  Fm1
......................................................................Cggacggagaacugauaaggg......................  9      1  Fm1
......................................................................uggacNgagaacugauaaggg......................  11     1  Fm1
......................................................................uggacggagaacuCauaaggg......................  5      1  Fm1
......................................................................ugNacggagaacugauaaggg......................  1      1  Fm1
......................................................................uggacggagUacugauaaggg......................  19     1  Fm1
......................................................................uggacggagaacugauaagUg......................  14     1  Fm1
......................................................................uggacggagaacugauaUggg......................  223    1  Fm1
......................................................................uggGcggagaacugauaaggg......................  14     1  Fm1
......................................................................Gggacggagaacugauaaggg......................  1      1  Fm1
......................................................................Nggacggagaacugauaaggg......................  26     1  Fm1
......................................................................ugCacggagaacugauaaggg......................  1      1  Fm1
......................................................................uggacggagCacugauaaggg......................  8      1  Fm1
......................................................................uggacggagaacugauCaggg......................  154    1  Fm1
......................................................................Aggacggagaacugauaaggg......................  24     1  Fm1
......................................................................uAgacggagaacugauaaggg......................  30     1  Fm1
......................................................................uggaAggagaacugauaaggg......................  1      1  Fm1
......................................................................uggacggagaacGgauaaggg......................  1      1  Fm1
......................................................................uggacggagaacugaAaaggg......................  2      1  Fm1
......................................................................uggacggagGacugauaaggg......................  59     1  Fm1
......................................................................uggacggagaacCgauaaggg......................  12     1  Fm1
......................................................................uggacggagaacugCuaaggg......................  4      1  Fm1
......................................................................uggCcggagaacugauaaggg......................  10     1  Fm1
......................................................................uggacCgagaacugauaaggg......................  4      1  Fm1
......................................................................uggacggaCaacugauaaggg......................  3      1  Fm1
......................................................................uggacggagaacugauaaUgg......................  8      1  Fm1
......................................................................uggacggagaCcugauaaggg......................  72     1  Fm1
......................................................................uggacggagaGcugauaaggg......................  136    1  Fm1
......................................................................uggacggagaacugauaaAgg......................  6      1  Fm1
......................................................................uggacUgagaacugauaaggg......................  15     1  Fm1
......................................................................ugUacggagaacugauaaggg......................  3      1  Fm1
......................................................................uggacggagaaUugauaaggg......................  20     1  Fm1
......................................................................uggacggagaacugaGaaggg......................  2      1  Fm1
......................................................................uggacggagaacugauaCggg......................  225    1  Fm1
......................................................................ugAacggagaacugauaaggg......................  12     1  Fm1
......................................................................uUgacggagaacugauaaggg......................  14     1  Fm1
......................................................................uggacgAagaacugauaaggg......................  8      1  Fm1
......................................................................uggacggagaacugauaagAg......................  22     1  Fm1
......................................................................uggacggCgaacugauaaggg......................  5      1  Fm1
......................................................................uggacggagaacugauaaggU......................  1894   1  Fm1
......................................................................uggacggagaacugauaaggC......................  348    1  Fm1
......................................................................uggacAgagaacugauaaggg......................  8      1  Fm1
......................................................................uggacggGgaacugauaaggg......................  18     1  Fm1
......................................................................uggacggaAaacugauaaggg......................  2      1  Fm1
......................................................................uggacgUagaacugauaaggg......................  16     1  Fm1
......................................................................uggacggagaacugauUaggg......................  93     1  Fm1
......................................................................uggacggagaacugUuaaggg......................  11     1  Fm1
......................................................................uggacgCagaacugauaaggg......................  3      1  Fm1
......................................................................Aggacggagaacugauaagggc.....................  153    1  Fm1
......................................................................uggacggagaacugauaagCgc.....................  50     1  Fm1
......................................................................uggacggagaacugaGaagggc.....................  21     1  Fm1
......................................................................uggacggagaacugauaaggCc.....................  116    1  Fm1
......................................................................uggacggagaacuAauaagggc.....................  170    1  Fm1
......................................................................uggCcggagaacugauaagggc.....................  174    1  Fm1
......................................................................uggacggagaCcugauaagggc.....................  672    1  Fm1
......................................................................uggacggagCacugauaagggc.....................  169    1  Fm1
......................................................................uggaUggagaacugauaagggc.....................  194    1  Fm1
......................................................................uggacggagaacugauaaCggc.....................  16     1  Fm1
......................................................................uCgacggagaacugauaagggc.....................  73     1  Fm1
......................................................................uggacggaUaacugauaagggc.....................  154    1  Fm1
......................................................................uggacggagaacugauaNgggc.....................  2      1  Fm1
......................................................................uggacggUgaacugauaagggc.....................  431    1  Fm1
......................................................................uggacggagaacugauaaAggc.....................  61     1  Fm1
```

```
                                  Star                                  Mature
cauuugugacaucagaaguaagucuguuucguguccucuucacuuuuccauccuguCuguacucgagaacuggacggagaacugauaagggcauagggcagaccacaucaaca
......................................................................uggacNgagaacugauaagggc.....................	135	1	Fm1
......................................................................uggacgNagaacugauaagggc.....................	46	1	Fm1
......................................................................uggacggagaacugauCagggc.....................	1755	1	Fm1
......................................................................uggacggagaacugauaagAgc.....................	127	1	Fm1
......................................................................uggacgUagaacugauaagggc.....................	178	1	Fm1
......................................................................uggacggagaacugauaaggUc.....................	223	1	Fm1
......................................................................Gggacggagaacugauaagggc.....................	22	1	Fm1
......................................................................uggacggagaacugauaagggU.....................	7010	1	Fm1
......................................................................ugUacggagaacugauaagggc.....................	131	1	Fm1
......................................................................uggacggagGacugauaagggc.....................	1684	1	Fm1
......................................................................uggaGggagaacugauaagggc.....................	38	1	Fm1
......................................................................uggacggagaacugauaUgggc.....................	2648	1	Fm1
......................................................................uggacggagaacCgauaagggc.....................	121	1	Fm1
......................................................................uUgacggagaacugauaagggc.....................	193	1	Fm1
......................................................................uggacggagaacugauUagggc.....................	1143	1	Fm1
......................................................................uggUcggagaacugauaagggc.....................	135	1	Fm1
......................................................................uggacggagaacugGuaagggc.....................	99	1	Fm1
......................................................................uggacggagaacAgauaagggc.....................	31	1	Fm1
......................................................................uggGcggagaacugauaagggc.....................	170	1	Fm1
......................................................................uggacggagaacugauaagggA.....................	21574	1	Fm1
......................................................................uggacggagaacugUuaagggc.....................	110	1	Fm1
......................................................................uggacggagaacuUauaagggc.....................	78	1	Fm1
......................................................................uAgacggagaacugauaagggc.....................	390	1	Fm1
......................................................................uggacggagaacugCuaagggc.....................	25	1	Fm1
......................................................................uggacggagaacGgauaagggc.....................	33	1	Fm1
......................................................................uggacUgagaacugauaagggc.....................	145	1	Fm1
......................................................................uggacggagaacugaAaagggc.....................	18	1	Fm1
......................................................................uggacgCagaacugauaagggc.....................	47	1	Fm1
......................................................................uggacggagaacugauaGgggc.....................	8811	1	Fm1
......................................................................uggacggagaacugauaaggAc.....................	313	1	Fm1
......................................................................uggacggagaacugauGagggc.....................	1216	1	Fm1
......................................................................Nggacggagaacugauaagggc.....................	440	1	Fm1
......................................................................uggaAggagaacugauaagggc.....................	66	1	Fm1
......................................................................uggacAgagaacugauaagggc.....................	137	1	Fm1
......................................................................ugAacggagaacugauaagggc.....................	183	1	Fm1
......................................................................uggacggagaacugauaagggc.....................	846342	0	Fm1
......................................................................uggacggagUacugauaagggc.....................	282	1	Fm1
......................................................................uggacggagaacugauaagggG.....................	874	1	Fm1
......................................................................uggacggaAaacugauaagggc.....................	49	1	Fm1
......................................................................ugCacggagaacugauaagggc.....................	65	1	Fm1
......................................................................uggacggagaacugauaaUggc.....................	128	1	Fm1
......................................................................uggacggagaaGugauaagggc.....................	18	1	Fm1
......................................................................ugNacggagaacugauaagggc.....................	4	1	Fm1
......................................................................uggacggagaacugauaagUgc.....................	175	1	Fm1
......................................................................uggacggagaaAugauaagggc.....................	22	1	Fm1
......................................................................uggacggCgaacugauaagggc.....................	86	1	Fm1
......................................................................uggacggaCaacugauaagggc.....................	44	1	Fm1
......................................................................uggacggagaaUugauaagggc.....................	284	1	Fm1
......................................................................uggacgAagaacugauaagggc.....................	98	1	Fm1
......................................................................uggacggagaacugauaCgggc.....................	3205	1	Fm1
......................................................................uggacggagaacugaCaagggc.....................	80	1	Fm1
......................................................................uggacggGgaacugauaagggc.....................	194	1	Fm1
......................................................................uNgacggagaacugauaagggc.....................	7	1	Fm1
......................................................................uggacCgagaacugauaagggc.....................	34	1	Fm1
......................................................................Cggacggagaacugauaagggc.....................	102	1	Fm1
......................................................................uggacggagaacuCauaagggc.....................	49	1	Fm1
......................................................................uggacggagaGcugauaagggc.....................	1253	1	Fm1
......................................................................uggacggagGacugauaagggca....................	27	1	Fm1
......................................................................Gggacggagaacugauaagggca....................	2	1	Fm1
......................................................................uggacggagaacugauaagggAa....................	1120	1	Fm1
......................................................................uggacggagaacugauCagggca....................	121	1	Fm1
......................................................................uggacgNagaacugauaagggca....................	6	1	Fm1
......................................................................uggacggaUaacugauaagggca....................	9	1	Fm1
......................................................................uggacggagaaAugauaagggca....................	2	1	Fm1
......................................................................uggacggagaacugauaCgggca....................	189	1	Fm1
......................................................................uAgacggagaacugauaagggca....................	24	1	Fm1
......................................................................uggacggagaacAgauaagggca....................	3	1	Fm1
......................................................................uggacggagaacugauaagUgca....................	15	1	Fm1
......................................................................uggacggaAaacugauaagggca....................	7	1	Fm1
......................................................................ugUacggagaacugauaagggca....................	12	1	Fm1
```

```
                                  Star                               Mature
cauuugugacaucagaaguaagucuguuucguguccucuucacuuuuccauccuguc uguacucgagaacuggacggagaacugauaagggcauagggcagaccacaucaaca
```

```
......................................................................uggacggagaacugauaUgggca.................... 195 1 Fm1
......................................................................uggacggagaacugauaaCggca.................... 2 1 Fm1
......................................................................uggacggCgaacugauaagggca.................... 2 1 Fm1
......................................................................uggacggagaaUugauaagggca.................... 17 1 Fm1
......................................................................uggacggagaacuAauaagggca.................... 13 1 Fm1
......................................................................Cggacggagaacugauaagggca.................... 5 1 Fm1
......................................................................uggacggagaacGgauaagggca.................... 2 1 Fm1
......................................................................uggCcggagaacugauaagggca.................... 8 1 Fm1
......................................................................uggacggagaacugUuaagggca.................... 11 1 Fm1
......................................................................uggacCgagaacugauaagggca.................... 4 1 Fm1
......................................................................uggacggagaacuUauaagggca.................... 3 1 Fm1
......................................................................uggacggagaacugGuaagggca.................... 6 1 Fm1
......................................................................uggacggagaacugauaaggAca.................... 12 1 Fm1
......................................................................uggaGggagaacugauaagggca.................... 2 1 Fm1
......................................................................uggacggaCaacugauaagggca.................... 2 1 Fm1
......................................................................uggacAgagaacugauaagggca.................... 7 1 Fm1
......................................................................uggacgUagaacugauaagggca.................... 15 1 Fm1
......................................................................uggacggagaacugauaagggUa.................... 193 1 Fm1
......................................................................uggacNgagaacugauaagggca.................... 9 1 Fm1
......................................................................uUgacggagaacugauaagggca.................... 4 1 Fm1
......................................................................uggacggagaacugauGagggca.................... 91 1 Fm1
......................................................................uggGcggagaacugauaagggca.................... 15 1 Fm1
......................................................................uggacUgagaacugauaagggca.................... 7 1 Fm1
......................................................................uggacggagaacugauUagggca.................... 85 1 Fm1
......................................................................uggacggagCacugauaagggca.................... 8 1 Fm1
......................................................................uggacggagaacugauaagggca.................... 56469 0 Fm1
......................................................................uggacggagaacugauaagAgca.................... 9 1 Fm1
......................................................................uggacggUgaacugauaagggca.................... 23 1 Fm1
......................................................................uggacgCagaacugauaagggca.................... 3 1 Fm1
......................................................................uggacggagaacuCauaagggca.................... 2 1 Fm1
......................................................................uNgacggagaacugauaagggca.................... 2 1 Fm1
......................................................................uCgacggagaacugauaagggca.................... 7 1 Fm1
......................................................................uggacggagaacugauaagggcG.................... 2275 1 Fm1
......................................................................uggacggagaacugaCaagggca.................... 2 1 Fm1
......................................................................Aggacggagaacugauaagggca.................... 9 1 Fm1
......................................................................uggacggagaacugauaagggcC.................... 616 1 Fm1
......................................................................uggacggagaacugauaaggCca.................... 15 1 Fm1
......................................................................uggacggagaacugCuaagggca.................... 2 1 Fm1
......................................................................uggacggagaacCgauaagggca.................... 18 1 Fm1
......................................................................uggacggagaacugauaGgggca.................... 399 1 Fm1
......................................................................uggacggagaCcugauaagggca.................... 44 1 Fm1
......................................................................uggacggagaacugauaaggUca.................... 22 1 Fm1
......................................................................uggUcggagaacugauaagggca.................... 4 1 Fm1
......................................................................Nggacggagaacugauaagggca.................... 28 1 Fm1
......................................................................ugNacggagaacugauaagggca.................... 2 1 Fm1
......................................................................ugCacggagaacugauaagggca.................... 4 1 Fm1
......................................................................uggaUggagaacugauaagggca.................... 16 1 Fm1
......................................................................uggacggagaGcugauaagggca.................... 78 1 Fm1
......................................................................uggacggagaacugauaaUggca.................... 8 1 Fm1
......................................................................uggacggagaacugauaagggGa.................... 6 1 Fm1
......................................................................uggacgAagaacugauaagggca.................... 6 1 Fm1
......................................................................uggacggGgaacugauaagggca.................... 13 1 Fm1
......................................................................uggacggagaUcugauaagggca.................... 1901 1 Fm1
......................................................................ugAacggagaacugauaagggca.................... 17 1 Fm1
......................................................................uggaAggagaacugauaagggca.................... 4 1 Fm1
......................................................................uggacggagUacugauaagggca.................... 17 1 Fm1
......................................................................uggacggagaaGugauaagggca.................... 1 1 Fm1
......................................................................uggacggagaacugauaagCgca.................... 4 1 Fm1
......................................................................uggacggagaacugauaaAggca.................... 7 1 Fm1
......................................................................uNgacggagaacugauaagggcau................... 1 1 Fm1
......................................................................uggacggagaacuAauaagggcau................... 2 1 Fm1
......................................................................uggacggagaUcugauaagggcau................... 151 1 Fm1
......................................................................uggacggagaacugauaUgggcau................... 8 1 Fm1
......................................................................Nggacggagaacugauaagggcau................... 1 1 Fm1
......................................................................uggacggagaacugauaagggAau................... 832 1 Fm1
......................................................................uggacggagaacuUauaagggcau................... 3 1 Fm1
......................................................................uggacggagaacugauGagggcau................... 7 1 Fm1
......................................................................uggacggagaacugauaagggcaA................... 139 1 Fm1
......................................................................uggacggCgaacugauaagggcau................... 2 1 Fm1
......................................................................uggUcggagaacugauaagggcau................... 1 1 Fm1
```

Star Mature

```
cauuugugacaucagaaguaagucuguuucguguccucuucacuuuuccauccugucuguacucgagaacuggacggagaacugauaagggcauagggcagaccacaucaaca
......................................................................uUgacggagaacugauaagggcau...................    2      1    Fm1
......................................................................uggacggUgaacugauaagggcau...................    2      1    Fm1
......................................................................uggacggagaacugauaaUggcau...................    1      1    Fm1
......................................................................uggacgUagaacugauaagggcau...................    1      1    Fm1
......................................................................uggacggagaacugauaagggUau...................    208    1    Fm1
......................................................................uggacggagaacugauUagggcau...................    9      1    Fm1
......................................................................uggacggagCacugauaagggcau...................    2      1    Fm1
......................................................................uggacggagaacugauaaggUcau...................    1      1    Fm1
......................................................................uggaGggagaacugauaagggcau...................    2      1    Fm1
......................................................................uggGcggagaacugauaagggcau...................    1      1    Fm1
......................................................................uggacggagaacugauaagggcGu...................    30     1    Fm1
......................................................................Aggacggagaacugauaagggcau...................    1      1    Fm1
......................................................................uggaAggagaacugauaagggcau...................    2      1    Fm1
......................................................................uCgacggagaacugauaagggcau...................    1      1    Fm1
......................................................................ugUacggagaacugauaagggcau...................    1      1    Fm1
......................................................................uggacggagaacugauaagggcCu...................    95     1    Fm1
......................................................................uggacggagaGcugauaagggcau...................    5      1    Fm1
......................................................................uggacggagaacugauaGgggcau...................    51     1    Fm1
......................................................................uggacggagaacugauCagggcau...................    12     1    Fm1
......................................................................uAgacggagaacugauaagggcau...................    2      1    Fm1
......................................................................uggacggagaacugauaagggcau...................    3686   0    Fm1
......................................................................uggacggagaacugauaCgggcau...................    10     1    Fm1
......................................................................uggacggagaacugauaagCgcau...................    1      1    Fm1
......................................................................uggacggagaacugauaagggcaC...................    138    1    Fm1
......................................................................uggacggagaacugauaagggcaG...................    69     1    Fm1
......................................................................uggacggagaacugauaagggcauG..................    1      1    Fm1
......................................................................uggacggagaacugauaagggAaua..................    4      1    Fm1
......................................................................uggacggagaacugauaagggcUua..................    30     1    Fm1
......................................................................uggacggagaacugauaagggcauC..................    2      1    Fm1
......................................................................uggacggagaacugauaagggcaua..................    2      0    Fm1
......................................................................uggacggagaacugauaagggcauU..................    654    1    Fm1
......................................................................uggacggagaacugauaagggcauaU.................    15     1    Fm1
......................................................................uggacggagaacugauaagggcauUg.................    1      1    Fm1
.......................................................................ggacggagaacugauaag........................    21     0    Fm1
.......................................................................ggacggagaacugauaaU........................    16     1    Fm1
.......................................................................ggacggagGacugauaag........................    1      1    Fm1
.......................................................................ggGcggagaacugauaag........................    1      1    Fm1
.......................................................................ggacggaAaacugauaag........................    1      1    Fm1
.......................................................................Ugacggagaacugauaag........................    1      1    Fm1
.......................................................................ggacggagaacugauaUg........................    1      1    Fm1
.......................................................................ggacggagaacugauaaA........................    8      1    Fm1
.......................................................................Ugacggagaacugauaagg.......................    2      1    Fm1
.......................................................................ggacggagaacGgauaagg.......................    1      1    Fm1
.......................................................................ggacggagaacugauaagA.......................    4      1    Fm1
.......................................................................ggacggagaaAugauaagg.......................    1      1    Fm1
.......................................................................ggacggagaacugauaagg.......................    60     0    Fm1
.......................................................................ggacggagaacugauaagU.......................    1      1    Fm1
.......................................................................ggacggagaacugauaaggA......................    16     1    Fm1
.......................................................................ggacCgagaacugauaaggg......................    1      1    Fm1
.......................................................................ggacggagaacugauaaggg......................    73     0    Fm1
.......................................................................ggacggagaacugauaaggU......................    4      1    Fm1
.......................................................................ggacggagaacugauaaggC......................    1      1    Fm1
.......................................................................ggacggagCacugauaaggg......................    1      1    Fm1
.......................................................................ggacggagaaAugauaaggg......................    1      1    Fm1
.......................................................................ggacggagaacugauGaggg......................    1      1    Fm1
.......................................................................ggacggagaacugauCaggg......................    1      1    Fm1
.......................................................................Ugacggagaacugauaaggg......................    4      1    Fm1
.......................................................................gUacggagaacugauaagggc.....................    1      1    Fm1
.......................................................................ggacggagaaGugauaagggc.....................    1      1    Fm1
.......................................................................ggacggCgaacugauaagggc.....................    4      1    Fm1
.......................................................................ggacggagaacugauUagggc.....................    1      1    Fm1
.......................................................................ggacggagaacugauaagggA.....................    51     1    Fm1
.......................................................................ggacggagUacugauaagggc.....................    2      1    Fm1
.......................................................................ggGcggagaacugauaagggc.....................    2      1    Fm1
.......................................................................ggacggagaacugauaaggUc.....................    2      1    Fm1
.......................................................................ggacggagaacugauaGgggc.....................    9      1    Fm1
.......................................................................ggacggagaacugauaagAgc.....................    2      1    Fm1
.......................................................................ggaGggagaacugauaagggc.....................    1      1    Fm1
.......................................................................ggacggagaacugauCagggc.....................    1      1    Fm1
.......................................................................ggacggagaacugauaaggCc.....................    4      1    Fm1
```

Star Mature

```
cauuugugacaucagaaguaagucuguuucguguccucuucacuuuuccauccugucuguacucgagaacuggacggagaacugauaagggcauagggcagaccacaucaaca
.......................................................................ggacggaUaacugauaagggc.....................	1	1	Fm1
.......................................................................ggacggagaaAugauaagggc.....................	2	1	Fm1
.......................................................................ggacggagaacugauaCgggc.....................	1	1	Fm1
.......................................................................ggacggUgaacugauaagggc.....................	1	1	Fm1
.......................................................................ggacggagaacugUuaagggc.....................	1	1	Fm1
.......................................................................ggacggagaacugauaUgggc.....................	1	1	Fm1
.......................................................................ggacUgagaacugauaagggc.....................	1	1	Fm1
.......................................................................ggacggagaacugauaaggAc.....................	1	1	Fm1
.......................................................................ggacggagGacugauaagggc.....................	4	1	Fm1
.......................................................................ggacgUagaacugauaagggc.....................	1	1	Fm1
.......................................................................ggacggagaacugauaagggc.....................	1101	0	Fm1
.......................................................................ggacggagaaUugauaagggc.....................	4	1	Fm1
.......................................................................ggacggagaacugauaagUgc.....................	2	1	Fm1
.......................................................................ggacggGgaacugauaagggc.....................	1	1	Fm1
.......................................................................ggacggagaacugauaagggU.....................	12	1	Fm1
.......................................................................Ugacggagaacugauaagggc.....................	45	1	Fm1
.......................................................................ggCcggagaacugauaagggc.....................	2	1	Fm1
.......................................................................ggacggagaCcugauaagggc.....................	3	1	Fm1
.......................................................................ggacggaAaacugauaagggc.....................	2	1	Fm1
.......................................................................ggaAggagaacugauaagggc.....................	4	1	Fm1
.......................................................................ggacggagCacugauaagggc.....................	16	1	Fm1
.......................................................................ggacggagaacugaAaagggc.....................	2	1	Fm1
.......................................................................ggacggagaacGgauaagggc.....................	1	1	Fm1
.......................................................................ggacggagaacCgauaagggc.....................	3	1	Fm1
.......................................................................ggacggagaacAgauaagggc.....................	1	1	Fm1
.......................................................................ggacggagaaAugauaagggca....................	1	1	Fm1
.......................................................................ggUcggagaacugauaagggca....................	1	1	Fm1
.......................................................................Ugacggagaacugauaagggca....................	1	1	Fm1
.......................................................................ggacggagaacugauaaCggca....................	1	1	Fm1
.......................................................................ggacggagaacugauaagggcG....................	4	1	Fm1
.......................................................................ggacggagaUcugauaagggca....................	8	1	Fm1
.......................................................................ggacggCgaacugauaagggca....................	1	1	Fm1
.......................................................................ggacggagaacGgauaagggca....................	1	1	Fm1
.......................................................................ggCcggagaacugauaagggca....................	1	1	Fm1
.......................................................................ggacggagaacugauaagggAa....................	4	1	Fm1
.......................................................................ggacggagCacugauaagggca....................	1	1	Fm1
.......................................................................ggacggagaacugauaagggca....................	76	0	Fm1
.......................................................................ggacggagaacugauaagggcau...................	4	0	Fm1
.......................................................................ggacggagaacugauaagggUau...................	2	1	Fm1
.......................................................................ggacggagaacugauaagggcaC...................	1	1	Fm1
........................................................................gacggagaacugauaagggc.....................	4	0	Fm1
........................................................................Uacggagaacugauaagggc.....................	3	1	Fm1
..........................................................................cggagaacugauaagggc.....................	3	0	Fm1
..........................................................................Uggagaacugauaagggc.....................	10	1	Fm1

....................................................................Ucuggacggagaacugauaagggc.....................	1	1	Mm1
.....................................................................cuggacggagaacugauaag........................	1	0	Mm1
.....................................................................cuggacggagaacugauaagA.......................	7	1	Mm1
.....................................................................cuggacggagCacugauaagg.......................	1	1	Mm1
.....................................................................cuggacggagaacugauaagg.......................	43	0	Mm1
.....................................................................cuggacggagaacugauaaggg......................	17	0	Mm1
.....................................................................cuggacggagCacugauaaggg......................	1	1	Mm1
.....................................................................cuggacggagaacugauaagggc.....................	45	0	Mm1
.....................................................................cuggacggagCacugauaagggc.....................	1	1	Mm1
.....................................................................cugUacggagaacugauaagggc.....................	1	1	Mm1
.....................................................................cuggacggagaacugauaagggca....................	5	0	Mm1
......................................................................uAgacggagaacugauaa.........................	2	1	Mm1
......................................................................uggacgUagaacugauaa.........................	1	1	Mm1
......................................................................uggacggagaacugauaG.........................	2	1	Mm1
......................................................................uggacggagaacugauaU.........................	29	1	Mm1
......................................................................uggacggagaGcugauaa.........................	1	1	Mm1
......................................................................Nggacggagaacugauaa.........................	2	1	Mm1
......................................................................uggacggagaacugauUa.........................	6	1	Mm1
......................................................................uggacggagaacugauCa.........................	3	1	Mm1
......................................................................uggacggagaacugauGa.........................	1	1	Mm1
......................................................................uggacggagaacugaCaa.........................	1	1	Mm1
......................................................................uggacggagaacuAauaa.........................	1	1	Mm1
......................................................................Aggacggagaacugauaa.........................	1	1	Mm1
......................................................................ugAacggagaacugauaa.........................	1	1	Mm1
......................................................................uggacgAagaacugauaa.........................	1	1	Mm1
```

Star Mature

```
cauuugugacaucagaaguaagucuguuucguguccucuucacuuuuccauccugucuguacucgagaacuggacggagaacugauaagggcauagggcagaccacaucaaca
......................................................................uggacggagaacugaAaa.........................  64    1  Mm1
......................................................................uggacggagaacugauaC.........................  2     1  Mm1
......................................................................uggacUgagaacugauaa.........................  1     1  Mm1
......................................................................uggacggagaacugauaa.........................  995   0  Mm1
......................................................................uggacggagaacugauUag........................  5     1  Mm1
......................................................................ugCacggagaacugauaag........................  1     1  Mm1
......................................................................uggacggaAaacugauaag........................  2     1  Mm1
......................................................................uggaUggagaacugauaag........................  1     1  Mm1
......................................................................uggacggagaacugauaaU........................  1409  1  Mm1
......................................................................uggacggagaacugauaCg........................  16    1  Mm1
......................................................................ugAacggagaacugauaag........................  1     1  Mm1
......................................................................Nggacggagaacugauaag........................  3     1  Mm1
......................................................................uggacggagaacugUuaag........................  2     1  Mm1
......................................................................uggacggagaacugauaGg........................  15    1  Mm1
......................................................................Aggacggagaacugauaag........................  3     1  Mm1
......................................................................uggacggagGacugauaag........................  6     1  Mm1
......................................................................uggacggagaGcugauaag........................  19    1  Mm1
......................................................................uggacggagaacugauaaA........................  2940  1  Mm1
......................................................................uggacggagUacugauaag........................  2     1  Mm1
......................................................................uggacAgagaacugauaag........................  2     1  Mm1
......................................................................uggacggagaacugauaag........................  3971  0  Mm1
......................................................................uggacggagaacugGuaag........................  1     1  Mm1
......................................................................uggacggagaacugauGag........................  3     1  Mm1
......................................................................uggacggagaacugauaUg........................  14    1  Mm1
......................................................................uggacgAagaacugauaag........................  1     1  Mm1
......................................................................uggacggagaacugauaaC........................  20    1  Mm1
......................................................................uggacggagaacugauCag........................  2     1  Mm1
......................................................................uggUcggagaacugauaag........................  1     1  Mm1
......................................................................uggacggagaCcugauaag........................  7     1  Mm1
......................................................................uAgacggagaacugauaag........................  2     1  Mm1
......................................................................uggacgCagaacugauaag........................  1     1  Mm1
......................................................................uggacgAagaacugauaagg.......................  1     1  Mm1
......................................................................uggacggagaGcugauaagg.......................  122   1  Mm1
......................................................................Aggacggagaacugauaagg.......................  53    1  Mm1
......................................................................uggacggagaacugauaCgg.......................  71    1  Mm1
......................................................................uggacggagaacugauaaAg.......................  24    1  Mm1
......................................................................uggacggagaacugauCagg.......................  29    1  Mm1
......................................................................uggacggagaacugaCaagg.......................  1     1  Mm1
......................................................................uggacggagaacugauaagU.......................  365   1  Mm1
......................................................................uggacgUagaacugauaagg.......................  7     1  Mm1
......................................................................uggacggagaacugGuaagg.......................  3     1  Mm1
......................................................................uggacggagaaUugauaagg.......................  14    1  Mm1
......................................................................uggacggagaacuCauaagg.......................  2     1  Mm1
......................................................................uggaGggagaacugauaagg.......................  1     1  Mm1
......................................................................uggacggagaacugauaUgg.......................  70    1  Mm1
......................................................................uggacggagaacugUuaagg.......................  2     1  Mm1
......................................................................uggUcggagaacugauaagg.......................  6     1  Mm1
......................................................................uggacUgagaacugauaagg.......................  3     1  Mm1
......................................................................uggacggagaacugauGagg.......................  40    1  Mm1
......................................................................uAgacggagaacugauaagg.......................  5     1  Mm1
......................................................................ugUacggagaacugauaagg.......................  3     1  Mm1
......................................................................uggaAggagaacugauaagg.......................  2     1  Mm1
......................................................................ugAacggagaacugauaagg.......................  1     1  Mm1
......................................................................uggacgCagaacugauaagg.......................  1     1  Mm1
......................................................................Cggacggagaacugauaagg.......................  3     1  Mm1
......................................................................uggGcggagaacugauaagg.......................  4     1  Mm1
......................................................................uggacggagaacCgauaagg.......................  4     1  Mm1
......................................................................uggacggagaacugaAaagg.......................  1     1  Mm1
......................................................................uggacggagaacugauaagC.......................  3     1  Mm1
......................................................................uggacggagaaGugauaagg.......................  1     1  Mm1
......................................................................uggacggCgaacugauaagg.......................  1     1  Mm1
......................................................................uggaUggagaacugauaagg.......................  3     1  Mm1
......................................................................uCgacggagaacugauaagg.......................  2     1  Mm1
......................................................................uggacggagCacugauaagg.......................  5     1  Mm1
......................................................................uggacggagaacugauUagg.......................  29    1  Mm1
......................................................................uggacggagaacuUauaagg.......................  1     1  Mm1
......................................................................uUgacggagaacugauaagg.......................  5     1  Mm1
......................................................................uggacggagaacugauaGgg.......................  77    1  Mm1
......................................................................uggacggGgaacugauaagg.......................  1     1  Mm1
......................................................................uggacAgagaacugauaagg.......................  5     1  Mm1
```

Star **Mature**

cauuugugacaucagaaguaagucuguuucguguccucuucacuuuuccauccugucuguacucgagaacuggacggagaacugauaagggcauagggcagaccacaucaaca			
....uggacggagUacugauaagg.......................	6	1	Mm1
....uggacggagaacugauaagg.......................	17362	0	Mm1
....uggacggagGacugauaagg.......................	8	1	Mm1
....uggacNgagaacugauaagg.......................	3	1	Mm1
....uggacggaUaacugauaagg.......................	1	1	Mm1
....uggacggUgaacugauaagg.......................	6	1	Mm1
....uggacggagaCcugauaagg.......................	70	1	Mm1
....uggacCgagaacugauaagg.......................	2	1	Mm1
....uggacggagaacugauaaUg.......................	1	1	Mm1
....uggacggagaacugauaagA.......................	1788	1	Mm1
....uggacggagaacAgauaagg.......................	2	1	Mm1
....ugCacggagaacugauaagg.......................	2	1	Mm1
....uggacggagaacugaGaagg.......................	2	1	Mm1
....Nggacggagaacugauaagg.......................	6	1	Mm1
....uggacggagaacugauaaggg......................	14223	0	Mm1
....uggacggagaacugauaCggg......................	59	1	Mm1
....uggacggagaacugauaaggU......................	413	1	Mm1
....uggacNgagaacugauaaggg......................	1	1	Mm1
....uggacggagCacugauaaggg......................	2	1	Mm1
....uggacgCagaacugauaaggg......................	1	1	Mm1
....uggacggagaacugauaaggC......................	5	1	Mm1
....uCgacggagaacugauaaggg......................	3	1	Mm1
....uUgacggagaacugauaaggg......................	1	1	Mm1
....uggacggagaacugauaUggg......................	41	1	Mm1
....uggacggagaacAgauaaggg......................	1	1	Mm1
....uggacggaUaacugauaaggg......................	1	1	Mm1
....uggGcggagaacugauaaggg......................	3	1	Mm1
....uggacggagaacugGuaaggg......................	5	1	Mm1
....uggacggagaacugUuaaggg......................	4	1	Mm1
....uggacgUagaacugauaaggg......................	2	1	Mm1
....uggacggagaacugauCaggg......................	29	1	Mm1
....uggacggagaacCgauaaggg......................	2	1	Mm1
....Aggacggagaacugauaaggg......................	31	1	Mm1
....uggacggagaacugauaagUg......................	3	1	Mm1
....uggacggagaacugauGaggg......................	18	1	Mm1
....Cggacggagaacugauaaggg......................	2	1	Mm1
....uggacggagaacugauaaggA......................	3716	1	Mm1
....uAgacggagaacugauaaggg......................	22	1	Mm1
....uggacggagaacugauaaUgg......................	3	1	Mm1
....uggacggagUacugauaaggg......................	7	1	Mm1
....uggacUgagaacugauaaggg......................	3	1	Mm1
....uggacggagaacuAauaaggg......................	3	1	Mm1
....uggacggagaaAugauaaggg......................	1	1	Mm1
....uggacggagaacugauaGggg......................	82	1	Mm1
....uggacggagaGcugauaaggg......................	85	1	Mm1
....Nggacggagaacugauaaggg......................	6	1	Mm1
....uggacggagaacugauaagCg......................	1	1	Mm1
....uggacggUgaacugauaaggg......................	4	1	Mm1
....ugAacggagaacugauaaggg......................	3	1	Mm1
....uggacAgagaacugauaaggg......................	3	1	Mm1
....uggacggGgaacugauaaggg......................	2	1	Mm1
....uggacggagaCcugauaaggg......................	33	1	Mm1
....uggacggagGacugauaaggg......................	3	1	Mm1
....uggacggCgaacugauaaggg......................	1	1	Mm1
....uggUcggagaacugauaaggg......................	3	1	Mm1
....uggacggagaacugauUaggg......................	24	1	Mm1
....ugUacggagaacugauaaggg......................	1	1	Mm1
....uggaUggagaacugauaaggg......................	1	1	Mm1
....uggacggagaaUugauaaggg......................	1	1	Mm1
....uggacggagUacugauaagggc.....................	49	1	Mm1
....uggacggagaacAgauaagggc.....................	5	1	Mm1
....Nggacggagaacugauaagggc.....................	48	1	Mm1
....uggGcggagaacugauaagggc.....................	25	1	Mm1
....uggUcggagaacugauaagggc.....................	13	1	Mm1
....uggacggUgaacugauaagggc.....................	47	1	Mm1
....uggacggagaGcugauaagggc.....................	209	1	Mm1
....uggacgAagaacugauaagggc.....................	8	1	Mm1
....uggacUgagaacugauaagggc.....................	17	1	Mm1
....uggCcggagaacugauaagggc.....................	24	1	Mm1
....uggacgUagaacugauaagggc.....................	17	1	Mm1

```
                                  Star                                Mature
cauuugugacaucagaaguaagucuguuucguguccucuucacuuuuccauccugucuguacucgagaacuggacggagaacugauaagggcauagggcagaccacaucaaca
......................................................................uggaGggagaacugauaagggc.....................   1      1   Mm1
......................................................................uggacggagaacugGuaagggc.....................   9      1   Mm1
......................................................................Aggacggagaacugauaagggc.....................   53     1   Mm1
......................................................................uggaUggagaacugauaagggc.....................   20     1   Mm1
......................................................................uggacggagaaUugauaagggc.....................   20     1   Mm1
......................................................................uggacggagaacugauaGgggc.....................   762    1   Mm1
......................................................................uggacgCagaacugauaagggc.....................   7      1   Mm1
......................................................................uggacAgagaacugauaagggc.....................   10     1   Mm1
......................................................................ugCacggagaacugauaagggc.....................   8      1   Mm1
......................................................................uggacggagaacugauaaggAc.....................   18     1   Mm1
......................................................................uUgacggagaacugauaagggc.....................   18     1   Mm1
......................................................................ugAacggagaacugauaagggc.....................   19     1   Mm1
......................................................................uggacggagaacugauaaUggc.....................   17     1   Mm1
......................................................................uggacggagaCcugauaagggc.....................   88     1   Mm1
......................................................................uggacggaCaacugauaagggc.....................   6      1   Mm1
......................................................................uggacggagaacugauUagggc.....................   148    1   Mm1
......................................................................uggacggagaacuAauaagggc.....................   18     1   Mm1
......................................................................uggaAggagaacugauaagggc.....................   8      1   Mm1
......................................................................uggacggagaaAugauaagggc.....................   6      1   Mm1
......................................................................uggacggGgaacugauaagggc.....................   32     1   Mm1
......................................................................uggacggagGacugauaagggc.....................   251    1   Mm1
......................................................................uggacggagaacugauNagggc.....................   1      1   Mm1
......................................................................uggacggagaacugaCaagggc.....................   11     1   Mm1
......................................................................uggacggagaacugauaagCgc.....................   8      1   Mm1
......................................................................uggacggagaacCgauaagggc.....................   10     1   Mm1
......................................................................uggacggagaacugauaagggA.....................   4855   1   Mm1
......................................................................uggacggagaaGugauaagggc.....................   2      1   Mm1
......................................................................ugUacggagaacugauaagggc.....................   10     1   Mm1
......................................................................uggacggagCacugauaagggc.....................   27     1   Mm1
......................................................................uggacggagaacugauaCgggc.....................   394    1   Mm1
......................................................................uggacgNagaacugauaagggc.....................   3      1   Mm1
......................................................................uggacggagaacugauaaAggc.....................   21     1   Mm1
......................................................................uggacNgagaacugauaagggc.....................   11     1   Mm1
......................................................................uggacggCgaacugauaagggc.....................   3      1   Mm1
......................................................................uggacggagaacugUuaagggc.....................   9      1   Mm1
......................................................................uggacggagaacugauaagUgc.....................   36     1   Mm1
......................................................................uggacggagaacugauaaggUc.....................   26     1   Mm1
......................................................................uggacggagaacugCuaagggc.....................   3      1   Mm1
......................................................................uggacggagaacugaAaagggc.....................   3      1   Mm1
......................................................................uggacggagaacuCauaagggc.....................   12     1   Mm1
......................................................................uggacggagaacugauaUgggc.....................   321    1   Mm1
......................................................................uggacggagaacugauaagAgc.....................   24     1   Mm1
......................................................................uAgacggagaacugauaagggc.....................   47     1   Mm1
......................................................................Cggacggagaacugauaagggc.....................   9      1   Mm1
......................................................................uNgacggagaacugauaagggc.....................   1      1   Mm1
......................................................................uggacggagaacugauaagggc.....................   99594  0   Mm1
......................................................................uggacggagaacGgauaagggc.....................   3      1   Mm1
......................................................................uCgacggagaacugauaagggc.....................   10     1   Mm1
......................................................................uggacggaAaacugauaagggc.....................   6      1   Mm1
......................................................................uggacggagaacugauGagggc.....................   154    1   Mm1
......................................................................uggacggagaacugauCagggc.....................   209    1   Mm1
......................................................................uggacggagaacugauaaggCc.....................   6      1   Mm1
......................................................................uggacggagaacugauaaCggc.....................   2      1   Mm1
......................................................................uggacggaUaacugauaagggc.....................   19     1   Mm1
......................................................................uggacggagaacugauaagggG.....................   16     1   Mm1
......................................................................uggacggagaacugauaagggU.....................   821    1   Mm1
......................................................................uggacggagaacuUauaagggc.....................   8      1   Mm1
......................................................................uggacUgagaacugauaagggca....................   2      1   Mm1
......................................................................uggacggCgaacugauaagggca....................   2      1   Mm1
......................................................................uggacggagaacugauaaggUca....................   4      1   Mm1
......................................................................uggacggagaGcugauaagggca....................   20     1   Mm1
......................................................................uggacggagaacugauaaCggca....................   1      1   Mm1
......................................................................uggacggagaaGugauaagggca....................   1      1   Mm1
......................................................................uggacggagaacugauUagggca....................   16     1   Mm1
......................................................................uggaUggagaacugauaagggca....................   1      1   Mm1
......................................................................Nggacggagaacugauaagggca....................   6      1   Mm1
......................................................................uggacggagGacugauaagggca....................   22     1   Mm1
......................................................................Cggacggagaacugauaagggca....................   3      1   Mm1
......................................................................uggacggagaacugauaaAggca....................   1      1   Mm1
......................................................................uggacggagaacugCuaagggca....................   1      1   Mm1
```

Star **Mature**

```
cauuugugacaucagaaguaagucuguuucguguccucuucacuuuuccauccugucuguacucgagaacuggacggagaacugauaagggcauagggcagaccacaucaaca
......................................................................uggacggagUacugauaagggca....................  4      1  Mm1
......................................................................uggacggagaacugauaUgggca....................  32     1  Mm1
......................................................................uggacggagaacugUuaagggca....................  3      1  Mm1
......................................................................uggacNgagaacugauaagggca....................  1      1  Mm1
......................................................................uggUcggagaacugauaagggca....................  4      1  Mm1
......................................................................uggacggagaCcugauaagggca....................  12     1  Mm1
......................................................................uggacgUagaacugauaagggca....................  2      1  Mm1
......................................................................uggacggagaaUugauaagggca....................  2      1  Mm1
......................................................................uggacggagaacugauaagggcC....................  44     1  Mm1
......................................................................uggacggagCacugauaagggca....................  5      1  Mm1
......................................................................uggacggagaacugauCagggca....................  20     1  Mm1
......................................................................uggacggagaacugauaagggUa....................  42     1  Mm1
......................................................................uAgacggagaacugauaagggca....................  3      1  Mm1
......................................................................uggacgNagaacugauaagggca....................  1      1  Mm1
......................................................................ugAacggagaacugauaagggca....................  4      1  Mm1
......................................................................uggacggagaacugGuaagggca....................  1      1  Mm1
......................................................................uggacggagaacugauaaggCca....................  4      1  Mm1
......................................................................uggacggagaacAgauaagggca....................  1      1  Mm1
......................................................................ugUacggagaacugauaagggca....................  2      1  Mm1
......................................................................uggacggagaacugauaagggcG....................  66     1  Mm1
......................................................................uggacggagaUcugauaagggca....................  1469   1  Mm1
......................................................................uggacggagaacugauaagCgca....................  1      1  Mm1
......................................................................uggacggagaacCgauaagggca....................  5      1  Mm1
......................................................................uggacggUgaacugauaagggca....................  2      1  Mm1
......................................................................uggacggagaacugauaagUgca....................  3      1  Mm1
......................................................................uggacggGgaacugauaagggca....................  4      1  Mm1
......................................................................uggacgAagaacugauaagggca....................  1      1  Mm1
......................................................................uggacggagaacugauaagAgca....................  1      1  Mm1
......................................................................uggacggagaacugauaagggca....................  11464  0  Mm1
......................................................................uggacggagaacugauaaggAca....................  2      1  Mm1
......................................................................uggCcggagaacugauaagggca....................  2      1  Mm1
......................................................................ugCacggagaacugauaagggca....................  2      1  Mm1
......................................................................uggacggaUaacugauaagggca....................  1      1  Mm1
......................................................................uUgacggagaacugauaagggca....................  3      1  Mm1
......................................................................Aggacggagaacugauaagggca....................  2      1  Mm1
......................................................................uggacggagaacugauGagggca....................  16     1  Mm1
......................................................................uggacggagaacuAauaagggca....................  3      1  Mm1
......................................................................uggGcggagaacugauaagggca....................  2      1  Mm1
......................................................................Gggacggagaacugauaagggca....................  1      1  Mm1
......................................................................uggacggagaacugauaagggAa....................  339    1  Mm1
......................................................................uggacAgagaacugauaagggca....................  1      1  Mm1
......................................................................uggacggagaacugauaCgggca....................  55     1  Mm1
......................................................................uggacggagaacugauaGgggca....................  57     1  Mm1
......................................................................uggacggagaacugauaagggGa....................  2      1  Mm1
......................................................................uggacggagaacugauaaUggca....................  1      1  Mm1
......................................................................uggacggagaacugauaCgggcau...................  2      1  Mm1
......................................................................uggacggagaacugauaGgggcau...................  4      1  Mm1
......................................................................uggacggagaacugauaagggcaA...................  30     1  Mm1
......................................................................uggacggagaacugauaagggcau...................  951    0  Mm1
......................................................................Cggacggagaacugauaagggcau...................  2      1  Mm1
......................................................................uggacggagaUcugauaagggcau...................  194    1  Mm1
......................................................................uggaUggagaacugauaagggcau...................  1      1  Mm1
......................................................................uggacggagaacugauGagggcau...................  2      1  Mm1
......................................................................uggacggagaacugauaagggcGu...................  7      1  Mm1
......................................................................uggacggagaacugauaagggcaG...................  18     1  Mm1
......................................................................uggacggagaacugaCaagggcau...................  1      1  Mm1
......................................................................uggacggagaacugauCagggcau...................  4      1  Mm1
......................................................................uggacggagaacugauaagggAau...................  202    1  Mm1
......................................................................uggacggagaacugauaagggcaC...................  14     1  Mm1
......................................................................uggacggagaacugauaagggcCu...................  5      1  Mm1
......................................................................uggacggagaacugauaagggUau...................  39     1  Mm1
......................................................................uggacggagaacugauaUgggcau...................  3      1  Mm1
......................................................................uggacggagaGcugauaagggcau...................  2      1  Mm1
......................................................................Nggacggagaacugauaagggcau...................  1      1  Mm1
......................................................................uggacggagaacugauUagggcau...................  2      1  Mm1
......................................................................uggacggagaacuAauaagggcau...................  1      1  Mm1
......................................................................uggacggagaacugauaagggcauC..................  5      1  Mm1
......................................................................uggacggagaacugauaagggcUua..................  5      1  Mm1
......................................................................uggacggagaacugauaagggcauU..................  101    1  Mm1
......................................................................uggacggagaacugauaagggcaua..................  2      0  Mm1
```

```
                                  Star                        Mature
cauuugugacaucagaaguaagucuguuucguguccucuucacuuuuccauccugucuguacucgagaacuggacggagaacugauaagggcauagggcagaccacaucaaca
......................................................................uggacggagaacugGuaagggcaua..................	1	1	Mm1
......................................................................uggacggagaUcugauaagggcauag.................	6	1	Mm1
......................................................................uggacggagaacugauaagggcauaU.................	2	1	Mm1
.......................................................................ggacggagaaAugauaag........................	1	1	Mm1
.......................................................................ggacggagaacugauaaA........................	9	1	Mm1
.......................................................................ggacggagaacugauaag........................	9	0	Mm1
.......................................................................ggacggagaacugauaagg.......................	27	0	Mm1
.......................................................................ggacggagaacugauaagU.......................	5	1	Mm1
.......................................................................ggacggagCacugauaagg.......................	1	1	Mm1
.......................................................................ggGcggagaacugauaaggg......................	1	1	Mm1
.......................................................................ggacggagaacugauaaggC......................	1	1	Mm1
.......................................................................ggacggagaacugauaaggg......................	43	0	Mm1
.......................................................................ggacggagaacugauaaggA......................	7	1	Mm1
.......................................................................Ugacggagaacugauaaggg......................	1	1	Mm1
.......................................................................ggacggagaacugauaagggU.....................	2	1	Mm1
.......................................................................ggacggagaacugauaagggA.....................	7	1	Mm1
.......................................................................ggacggagGacugauaagggc.....................	2	1	Mm1
.......................................................................ggacggagaCcugauaagggc.....................	1	1	Mm1
.......................................................................ggacggagCacugauaagggc.....................	1	1	Mm1
.......................................................................ggacggagaacGgauaagggc.....................	1	1	Mm1
.......................................................................ggacggagaaGugauaagggc.....................	2	1	Mm1
.......................................................................ggGcggagaacugauaagggc.....................	2	1	Mm1
.......................................................................ggacggagaacugauaaUggc.....................	1	1	Mm1
.......................................................................ggacggagaaAugauaagggc.....................	1	1	Mm1
.......................................................................ggacggaAaacugauaagggc.....................	1	1	Mm1
.......................................................................Ugacggagaacugauaagggc.....................	4	1	Mm1
.......................................................................ggacggagaacugauaaggAc.....................	1	1	Mm1
.......................................................................ggacggagaacugauaagggc.....................	178	0	Mm1
.......................................................................ggaAggagaacugauaagggc.....................	1	1	Mm1
.......................................................................ggacggagaUcugauaagggca....................	10	1	Mm1
.......................................................................ggacggagaacugauaaggCca....................	1	1	Mm1
.......................................................................ggacggagaacugauaagggca....................	17	0	Mm1
.......................................................................ggacggagaacugauaagggAau...................	3	1	Mm1
..........................................................................Uggagaacugauaagggc.....................	1	1	Mm1

..................................ccucuucacuuuuccauccugu.........................................................	28	0	Mm2
..................................ccucuucacuuuuccauccuguU........................................................	1	1	Mm2
..................................ccucuucacuuuuccauccugucuguacuc.................................................	3	0	Mm2
..................................ccucuucacuuuuccauccugucuguacucgagaa............................................	1	0	Mm2
....................................ucuucacuuuuccauccugucuguacu..................................................	1	0	Mm2
..........................................cuuuuccauccugucuguacucgaga.............................................	1	0	Mm2
........................................................cuguacucgagaacuggacggagaacugauaagggc.....................	1	0	Mm2
..........................................................guacucgagaacuggacggagaacugauaagggc.....................	2	0	Mm2
.............................................................cucgagaacuggacggagaacugauaagggc.....................	1	0	Mm2
...............................................................cgagaacuggacggagaacugauaaggg......................	1	0	Mm2
...............................................................cgagaacuggacggagaacugauaagggc.....................	2	0	Mm2
...................................................................aUcuggacggagaacugauaagggc.....................	3	1	Mm2
....................................................................acuggacggagaacugauaag........................	1	0	Mm2
....................................................................acuggacggagaacugauaagg.......................	2	0	Mm2
....................................................................Ucuggacggagaacugauaaggg......................	2	1	Mm2
....................................................................Ucuggacggagaacugauaagggc.....................	8	1	Mm2
....................................................................Ucuggacggagaacugauaagggca....................	1	1	Mm2
.....................................................................cuggacggagaacugaua..........................	1	0	Mm2
.....................................................................cuggacggagaacugauaa.........................	3	0	Mm2
.....................................................................cuggacggagaacugauaaA........................	6	1	Mm2
.....................................................................cuggacggagaacugauaaU........................	1	1	Mm2
.....................................................................Uuggacggagaacugauaag........................	1	1	Mm2
.....................................................................cuggacggagaacugauaag........................	21	0	Mm2
.....................................................................cuggacggagaacugaCaagg.......................	1	1	Mm2
.....................................................................cuggacggagaacugauaagU.......................	1	1	Mm2
.....................................................................cuggacggagaacugauaagA.......................	11	1	Mm2
.....................................................................cuggacggagaacugauUagg.......................	1	1	Mm2
.....................................................................cuggacggagaacugauaagg.......................	71	0	Mm2
.....................................................................cuggacggagaacugauaaggg......................	97	0	Mm2
.....................................................................cuggacggagaacugauaaggA......................	8	1	Mm2
.....................................................................cuggGcggagaacugauaaggg......................	1	1	Mm2
.....................................................................cuggacggagaacugauaaggC......................	1	1	Mm2
.....................................................................cuggacggagaacugauaaggU......................	16	1	Mm2
.....................................................................cuggacggagaacugUuaaggg......................	1	1	Mm2
.....................................................................cuggacggagaacugauGagggc.....................	1	1	Mm2
```

Star **Mature**

cauuugugacaucagaaguaagucuguuucguguccucuucacuuuuccauccuguCuguacucgagaacuggacggagaacugauaagggcauagggcagaccacaucaaca			
....cuggacggagaacugauaagggU.....................	5	1	Mm2
....cuggacggagaacugaCaagggc.....................	1	1	Mm2
....cuggacggagaacugauaGgggc.....................	1	1	Mm2
....Auggacggagaacugauaagggc.....................	2	1	Mm2
....cuggacggagaacugauaagggc.....................	233	0	Mm2
....cAggacggagaacugauaagggc.....................	1	1	Mm2
....cuggacggagaacugauaagggA.....................	2	1	Mm2
....Uuggacggagaacugauaagggc.....................	1	1	Mm2
....cugAacggagaacugauaagggc.....................	1	1	Mm2
....cuggacggagaUcugauaagggca....................	2	1	Mm2
....cuggacggagaacugauaagggca....................	16	0	Mm2
....cuggacUgagaacugauaagggca....................	1	1	Mm2
....cuggacggagaacugauaagggcC....................	2	1	Mm2
....uggacggagaacugauUa.........................	6	1	Mm2
....uggacggagaacugauGa.........................	2	1	Mm2
....uggacgCagaacugauaa.........................	1	1	Mm2
....uggacggGgaacugauaa.........................	1	1	Mm2
....ugCacggagaacugauaa.........................	2	1	Mm2
....uggacggagaacugUuaa.........................	1	1	Mm2
....uggacggagaacugaAaa.........................	136	1	Mm2
....uggacggagaacugauaa.........................	1306	0	Mm2
....uggacggagaacugauaG.........................	9	1	Mm2
....uggacggagaGcugauaa.........................	1	1	Mm2
....uggUcggagaacugauaa.........................	2	1	Mm2
....uggacggagaacugauaC.........................	8	1	Mm2
....uggacggagaacugauCa.........................	1	1	Mm2
....uggacggUgaacugauaa.........................	2	1	Mm2
....uggGcggagaacugauaa.........................	1	1	Mm2
....uggacggagaacugauaU.........................	39	1	Mm2
....Nggacggagaacugauaag........................	4	1	Mm2
....Cggacggagaacugauaag........................	2	1	Mm2
....uggacggagaacCgauaag........................	3	1	Mm2
....uggacUgagaacugauaag........................	3	1	Mm2
....ugCacggagaacugauaag........................	1	1	Mm2
....uggacggagaacugaGaag........................	1	1	Mm2
....uggacggagaacugGuaag........................	1	1	Mm2
....uggacggagaacugauaCg........................	33	1	Mm2
....uUgacggagaacugauaag........................	1	1	Mm2
....uggaUggagaacugauaag........................	1	1	Mm2
....uggacgCagaacugauaag........................	1	1	Mm2
....uggacggagaacugauaaU........................	1753	1	Mm2
....uggacggagaacugauaGg........................	24	1	Mm2
....uggacggGgaacugauaag........................	2	1	Mm2
....uggacggagGacugauaag........................	12	1	Mm2
....uggacggagaacugauCag........................	40	1	Mm2
....ugAacggagaacugauaag........................	2	1	Mm2
....uggGcggagaacugauaag........................	1	1	Mm2
....uggacggagaacuCauaag........................	1	1	Mm2
....uggaAggagaacugauaag........................	1	1	Mm2
....uggacgUagaacugauaag........................	1	1	Mm2
....uggacggagaacugauaag........................	8357	0	Mm2
....uggacggUgaacugauaag........................	1	1	Mm2
....uggacggagaacuAauaag........................	2	1	Mm2
....uggacggagaacugaAaag........................	2	1	Mm2
....uggacggagaacugauGag........................	6	1	Mm2
....uCgacggagaacugauaag........................	1	1	Mm2
....uggacggagaacugaCaag........................	1	1	Mm2
....uggacggaUaacugauaag........................	2	1	Mm2
....uggacggagUacugauaag........................	2	1	Mm2
....uggacggagaacugUuaag........................	1	1	Mm2
....uggacggagaGcugauaag........................	20	1	Mm2
....uggacAgagaacugauaag........................	1	1	Mm2
....uggacggagaacugauaaA........................	3696	1	Mm2
....uAgacggagaacugauaag........................	4	1	Mm2
....uggacggagaacugauaUg........................	26	1	Mm2
....uggacggagaacugauaaC........................	11	1	Mm2
....Aggacggagaacugauaag........................	2	1	Mm2
....uggacggagaCcugauaag........................	10	1	Mm2
....uNgacggagaacugauaag........................	1	1	Mm2
....uggacggagaacugauUag........................	12	1	Mm2

Star **Mature**

cauuugugacaucagaaguaagucuguuucguguccucuucacuuuuccauccugucuguacucgagaacuggacggagaacugauaagggcauagggcagaccacaucaaca			
....uggUcggagaacugauaag........................	1	1	Mm2
....uggacNgagaacugauaag........................	1	1	Mm2
....uggaUggagaacugauaagg.......................	1	1	Mm2
....uggacggaAaacugauaagg.......................	1	1	Mm2
....uggacggagaacugaCaagg.......................	3	1	Mm2
....uggCcggagaacugauaagg.......................	3	1	Mm2
....uggacggaUaacugauaagg.......................	4	1	Mm2
....uggacggagaGcugauaagg.......................	103	1	Mm2
....uggacggagaacugauaagg.......................	18110	0	Mm2
....uggacggagaacuCauaagg.......................	4	1	Mm2
....uggacggagaCcugauaagg.......................	60	1	Mm2
....uggacggagaacGgauaagg.......................	1	1	Mm2
....uggacggagaacugUuaagg.......................	1	1	Mm2
....uCgacggagaacugauaagg.......................	5	1	Mm2
....uggacggagUacugauaagg.......................	3	1	Mm2
....Cggacggagaacugauaagg.......................	4	1	Mm2
....uggGcggagaacugauaagg.......................	4	1	Mm2
....uggacggagaacugauaagC.......................	10	1	Mm2
....uggacUgagaacugauaagg.......................	11	1	Mm2
....uUgacggagaacugauaagg.......................	5	1	Mm2
....uggacggagaacugauUagg.......................	30	1	Mm2
....Gggacggagaacugauaagg.......................	1	1	Mm2
....uggacggagaacugGuaagg.......................	2	1	Mm2
....uggacggagaacugauaagU.......................	554	1	Mm2
....uggacgUagaacugauaagg.......................	8	1	Mm2
....uggacggagaacugauaagA.......................	2256	1	Mm2
....uggacggagCacugauaagg.......................	2	1	Mm2
....Aggacggagaacugauaagg.......................	32	1	Mm2
....uggacgAagaacugauaagg.......................	1	1	Mm2
....uggacgNagaacugauaagg.......................	3	1	Mm2
....uggacggagaacuUauaagg.......................	1	1	Mm2
....uggacggagGacugauaagg.......................	24	1	Mm2
....uggaGggagaacugauaagg.......................	1	1	Mm2
....uggacggagaacugauaaAg.......................	116	1	Mm2
....uggacAgagaacugauaagg.......................	4	1	Mm2
....uggacggagaacugauGagg.......................	16	1	Mm2
....uggacggUgaacugauaagg.......................	4	1	Mm2
....uggacNgagaacugauaagg.......................	3	1	Mm2
....uggacggagaacugauCagg.......................	35	1	Mm2
....Nggacggagaacugauaagg.......................	7	1	Mm2
....uggacggagaacugauaCgg.......................	71	1	Mm2
....uggacggagaaUugauaagg.......................	3	1	Mm2
....ugAacggagaacugauaagg.......................	5	1	Mm2
....uggacggagaacCgauaagg.......................	2	1	Mm2
....uggacggagaacugauaUgg.......................	63	1	Mm2
....uggUcggagaacugauaagg.......................	1	1	Mm2
....ugUacggagaacugauaagg.......................	6	1	Mm2
....uggacggagaacugauaaUg.......................	1	1	Mm2
....uggacggagaacuAauaagg.......................	1	1	Mm2
....uAgacggagaacugauaagg.......................	13	1	Mm2
....ugCacggagaacugauaagg.......................	1	1	Mm2
....uggaAggagaacugauaagg.......................	2	1	Mm2
....uggacggagaacugauaGgg.......................	90	1	Mm2
....uggacggGgaacugauaagg.......................	1	1	Mm2
....uggacggagaacugauaUggg......................	129	1	Mm2
....uggacggagaacugauaCggg......................	173	1	Mm2
....uggacggagaacugauaaUgg......................	5	1	Mm2
....uggaUggagaacugauaaggg......................	9	1	Mm2
....uggacggagaacCgauaaggg......................	7	1	Mm2
....uggacggagaacugauaaAgg......................	5	1	Mm2
....uggacggagaacugUuaaggg......................	6	1	Mm2
....uggacggagaaUugauaaggg......................	16	1	Mm2
....uggacggUgaacugauaaggg......................	8	1	Mm2
....Gggacggagaacugauaaggg......................	1	1	Mm2
....uggacgCagaacugauaaggg......................	5	1	Mm2
....uggacggaUaacugauaaggg......................	8	1	Mm2
....ugCacggagaacugauaaggg......................	4	1	Mm2
....uggacggagaacugauaagAg......................	15	1	Mm2
....uggacgNagaacugauaaggg......................	4	1	Mm2
....uggacggagaacugauaagCg......................	3	1	Mm2

```
                                  Star                                  Mature
cauuugugacaucagaaguaagucuguuucguguccucuucacuuuuccauccugucuguacucgagaacuggacggagaacugauaagggcauagggcagaccacaucaaca
......................................................................uggacNgagaacugauaaggg......................  9      1  Mm2
......................................................................uggacAgagaacugauaaggg......................  12     1  Mm2
......................................................................Nggacggagaacugauaaggg......................  22     1  Mm2
......................................................................uggacggagaacugCuaaggg......................  5      1  Mm2
......................................................................uggacggagaacugauaaggC......................  215    1  Mm2
......................................................................ugUacggagaacugauaaggg......................  8      1  Mm2
......................................................................uggacggGgaacugauaaggg......................  10     1  Mm2
......................................................................uggacggagaaGugauaaggg......................  1      1  Mm2
......................................................................uggCcggagaacugauaaggg......................  3      1  Mm2
......................................................................uggacggagaacugaCaaggg......................  4      1  Mm2
......................................................................uggacggagaGcugauaaggg......................  158    1  Mm2
......................................................................uggacggaCaacugauaaggg......................  4      1  Mm2
......................................................................uggacggagaacugauaGggg......................  262    1  Mm2
......................................................................Cggacggagaacugauaaggg......................  8      1  Mm2
......................................................................uggacggagaacugauGaggg......................  49     1  Mm2
......................................................................uggacggagaacuCauaaggg......................  3      1  Mm2
......................................................................uggacggagaacugGuaaggg......................  6      1  Mm2
......................................................................uggacUgagaacugauaaggg......................  14     1  Mm2
......................................................................uggaAggagaacugauaaggg......................  3      1  Mm2
......................................................................uggacggagaacuAauaaggg......................  7      1  Mm2
......................................................................uggacggagaacugauaagUg......................  6      1  Mm2
......................................................................uggacggagaacugauaaggA......................  5255   1  Mm2
......................................................................uggacggagaacugauaaCgg......................  1      1  Mm2
......................................................................uggUcggagaacugauaaggg......................  2      1  Mm2
......................................................................uggacggagaacGgauaaggg......................  4      1  Mm2
......................................................................uAgacggagaacugauaaggg......................  11     1  Mm2
......................................................................uggacCgagaacugauaaggg......................  1      1  Mm2
......................................................................ugAacggagaacugauaaggg......................  14     1  Mm2
......................................................................uggaGggagaacugauaaggg......................  1      1  Mm2
......................................................................uggacggagaacugauCaggg......................  83     1  Mm2
......................................................................uggacggagaacugauaaggg......................  41055  0  Mm2
......................................................................uggacggagaacugauaaggU......................  899    1  Mm2
......................................................................Aggacggagaacugauaaggg......................  41     1  Mm2
......................................................................uggacggagCacugauaaggg......................  3      1  Mm2
......................................................................uggGcggagaacugauaaggg......................  7      1  Mm2
......................................................................uggacgAagaacugauaaggg......................  9      1  Mm2
......................................................................uggacggagUacugauaaggg......................  15     1  Mm2
......................................................................uggacggagGacugauaaggg......................  50     1  Mm2
......................................................................uggacggaAaacugauaaggg......................  3      1  Mm2
......................................................................uggacggagaaAugauaaggg......................  3      1  Mm2
......................................................................uggacggagaacuUauaaggg......................  11     1  Mm2
......................................................................uggacggCgaacugauaaggg......................  4      1  Mm2
......................................................................uggacggagaacugauUaggg......................  62     1  Mm2
......................................................................uggacggagaCcugauaaggg......................  90     1  Mm2
......................................................................uggacgUagaacugauaaggg......................  10     1  Mm2
......................................................................uUgacggagaacugauaaggg......................  12     1  Mm2
......................................................................uggacggagaacugauaaUggc.....................  76     1  Mm2
......................................................................uggacggagaaAugauaagggc.....................  14     1  Mm2
......................................................................uggacggagaacugaGaagggc.....................  8      1  Mm2
......................................................................uggacggaAaacugauaagggc.....................  31     1  Mm2
......................................................................uggacggaCaacugauaagggc.....................  27     1  Mm2
......................................................................uggacggagaacugauaagCgc.....................  25     1  Mm2
......................................................................uggacggagaacugauCagggc.....................  1033   1  Mm2
......................................................................uggacgCagaacugauaagggc.....................  18     1  Mm2
......................................................................uggacggagaacAgauaagggc.....................  20     1  Mm2
......................................................................ugCacggagaacugauaagggc.....................  46     1  Mm2
......................................................................uggacggagaacugCuaagggc.....................  17     1  Mm2
......................................................................uggacggagaacugUuaagggc.....................  68     1  Mm2
......................................................................uggaUggagaacugauaagggc.....................  99     1  Mm2
......................................................................uggacggagaacugGuaagggc.....................  51     1  Mm2
......................................................................uggacggagaacugauaaCggc.....................  14     1  Mm2
......................................................................uggacggagGacugauaagggc.....................  998    1  Mm2
......................................................................Nggacggagaacugauaagggc.....................  258    1  Mm2
......................................................................uggacggagUacugauaagggc.....................  112    1  Mm2
......................................................................ugUacggagaacugauaagggc.....................  92     1  Mm2
......................................................................uggacggagaacuUauaagggc.....................  45     1  Mm2
......................................................................uggacggagaGcugauaagggc.....................  844    1  Mm2
......................................................................uNgacggagaacugauaagggc.....................  7      1  Mm2
......................................................................uggacggagaacugauaaggCc.....................  101    1  Mm2
......................................................................uggacggagaacugauaCgggc.....................  1933   1  Mm2
```

```
                                             Star                              Mature
cauuugugacaucagaaguaagucuguuucguguccucuucacuuuuccauccugucuguacucgagaacuggacggagaacugauaagggcauagggcagaccacaucaaca
......................................................................Aggacggagaacugauaagggc.....................  129     1  Mm2
......................................................................uggacggagaacugauaagggA.....................  11213   1  Mm2
......................................................................uggUcggagaacugauaagggc.....................  89      1  Mm2
......................................................................uggacggagaacugauaagggU.....................  3929    1  Mm2
......................................................................uggacggaUaacugauaagggc.....................  93      1  Mm2
......................................................................uggacggagaacuCauaagggc.....................  36      1  Mm2
......................................................................uggCcggagaacugauaagggc.....................  116     1  Mm2
......................................................................uggacggagaacGgauaagggc.....................  22      1  Mm2
......................................................................uggacggagaacugauaaggAc.....................  162     1  Mm2
......................................................................uggacNgagaacugauaagggc.....................  79      1  Mm2
......................................................................uggaAggagaacugauaagggc.....................  29      1  Mm2
......................................................................uggacAgagaacugauaagggc.....................  82      1  Mm2
......................................................................uggacggagaacugauaNgggc.....................  1       1  Mm2
......................................................................uggacggagaaGugauaagggc.....................  14      1  Mm2
......................................................................uggacUgagaacugauaagggc.....................  84      1  Mm2
......................................................................uCgacggagaacugauaagggc.....................  37      1  Mm2
......................................................................uggacggagaacCgauaagggc.....................  74      1  Mm2
......................................................................uggacggagaacugauaagUgc.....................  132     1  Mm2
......................................................................uggacggagaacuAauaagggc.....................  105     1  Mm2
......................................................................uggacggagaacugauaaAggc.....................  46      1  Mm2
......................................................................uUgacggagaacugauaagggc.....................  92      1  Mm2
......................................................................uggacggagCacugauaagggc.....................  55      1  Mm2
......................................................................uggacggagaacugaCaagggc.....................  45      1  Mm2
......................................................................uggacgAagaacugauaagggc.....................  63      1  Mm2
......................................................................uggacggagaCcugauaagggc.....................  435     1  Mm2
......................................................................uggacCgagaacugauaagggc.....................  13      1  Mm2
......................................................................Gggacggagaacugauaagggc.....................  27      1  Mm2
......................................................................Cggacggagaacugauaagggc.....................  64      1  Mm2
......................................................................uggaGggagaacugauaagggc.....................  18      1  Mm2
......................................................................uggacggagaacugauaagAgc.....................  84      1  Mm2
......................................................................uggacggagaacugauGagggc.....................  745     1  Mm2
......................................................................uggacgUagaacugauaagggc.....................  127     1  Mm2
......................................................................uggacggagaacugauUagggc.....................  638     1  Mm2
......................................................................uggacggagaacugaAaagggc.....................  14      1  Mm2
......................................................................uggacggagaacugauaUgggc.....................  1591    1  Mm2
......................................................................uggacggagaacugauaGgggc.....................  5049    1  Mm2
......................................................................uggGcggagaacugauaagggc.....................  131     1  Mm2
......................................................................uggacggCgaacugauaagggc.....................  23      1  Mm2
......................................................................ugAacggagaacugauaagggc.....................  103     1  Mm2
......................................................................uggacggagaacugauaaggUc.....................  123     1  Mm2
......................................................................uggacggGgaacugauaagggc.....................  113     1  Mm2
......................................................................ugNacggagaacugauaagggc.....................  5       1  Mm2
......................................................................uggacggagaacugauaagggG.....................  327     1  Mm2
......................................................................uggacggagaaUugauaagggc.....................  79      1  Mm2
......................................................................uggacggUgaacugauaagggc.....................  113     1  Mm2
......................................................................uAgacggagaacugauaagggc.....................  244     1  Mm2
......................................................................uggacggagaacugauaagggc.....................  505969  0  Mm2
......................................................................uggacgNagaacugauaagggc.....................  22      1  Mm2
......................................................................uggUcggagaacugauaagggca....................  7       1  Mm2
......................................................................uggacggagaacugauaagUgca....................  12      1  Mm2
......................................................................uggacggagaacuAauaagggca....................  9       1  Mm2
......................................................................uggacUgagaacugauaagggca....................  7       1  Mm2
......................................................................uggacggagaUcugauaagggca....................  2228    1  Mm2
......................................................................uggacggagaacCgauaagggca....................  5       1  Mm2
......................................................................uggacggagaacugauGagggca....................  47      1  Mm2
......................................................................uggacggagUacugauaagggca....................  7       1  Mm2
......................................................................uggacggagaacugCuaagggca....................  2       1  Mm2
......................................................................uUgacggagaacugauaagggca....................  7       1  Mm2
......................................................................uggacggagaacugauaagCgca....................  4       1  Mm2
......................................................................uggacggUgaacugauaagggca....................  11      1  Mm2
......................................................................uAgacggagaacugauaagggca....................  14      1  Mm2
......................................................................uggacggagCacugauaagggca....................  4       1  Mm2
......................................................................uggacggagaGcugauaagggca....................  60      1  Mm2
......................................................................uCgacggagaacugauaagggca....................  4       1  Mm2
......................................................................uggacggagaacAgauaagggca....................  1       1  Mm2
......................................................................uggacAgagaacugauaagggca....................  3       1  Mm2
......................................................................uggacgCagaacugauaagggca....................  1       1  Mm2
......................................................................uggacggagaacGgauaagggca....................  2       1  Mm2
......................................................................ugUacggagaacugauaagggca....................  8       1  Mm2
......................................................................Cggacggagaacugauaagggca....................  6       1  Mm2
```

Star Mature

cauuugugacaucagaaguaagucuguuucguguccucuucacuuuuccauccugucuguacucgagaacuggacggagaacugauaagggcauagggcagaccacaucaaca

....uggacggagaacugauaagggAa....................	612	1	Mm2
....uggacggGgaacugauaagggca....................	9	1	Mm2
....uggacggagaacugauaagggcG....................	1203	1	Mm2
....uggacggagGacugauaagggca....................	39	1	Mm2
....ugCacggagaacugauaagggca....................	3	1	Mm2
....uggacggagaacugauaaggUca....................	12	1	Mm2
....uggCcggagaacugauaagggca....................	9	1	Mm2
....uggacggagaacugaAaagggca....................	1	1	Mm2
....uggacgUagaacugauaagggca....................	6	1	Mm2
....uggacggagaacugauaagAgca....................	2	1	Mm2
....uggacggagaacugGuaagggca....................	3	1	Mm2
....uggGcggagaacugauaagggca....................	11	1	Mm2
....uggacggagaacugauaagggUa....................	161	1	Mm2
....uggacggagaacugauUagggca....................	58	1	Mm2
....uggacggagaacugauCagggca....................	64	1	Mm2
....uggacggagaacugauaagggcC....................	305	1	Mm2
....uggacggagaacugauaGgggca....................	238	1	Mm2
....Aggacggagaacugauaagggca....................	3	1	Mm2
....Nggacggagaacugauaagggca....................	24	1	Mm2
....uggacggagaacuUauaagggca....................	5	1	Mm2
....uggacggagaacugauaagggca....................	36577	0	Mm2
....uggacggagaacugauaaUggca....................	9	1	Mm2
....uggacggagaacugaCaagggca....................	3	1	Mm2
....uggacggagaCcugauaagggca....................	31	1	Mm2
....uggacggagaacuCauaagggca....................	4	1	Mm2
....uggacggagaacugauaaAggca....................	5	1	Mm2
....uggacggagaacugauaCgggca....................	145	1	Mm2
....uggacggagaaUugauaagggca....................	7	1	Mm2
....uggacggagaacugauaaggCca....................	27	1	Mm2
....uggacCgagaacugauaagggca....................	1	1	Mm2
....uggacggagaacugUuaagggca....................	6	1	Mm2
....uggacggCgaacugauaagggca....................	3	1	Mm2
....uggacggagaacugauaaggAca....................	10	1	Mm2
....uggaUggagaacugauaagggca....................	10	1	Mm2
....uggacNgagaacugauaagggca....................	8	1	Mm2
....uggaAggagaacugauaagggca....................	2	1	Mm2
....uggacggaUaacugauaagggca....................	10	1	Mm2
....uggacggaAaacugauaagggca....................	3	1	Mm2
....uggacgAagaacugauaagggca....................	6	1	Mm2
....ugAacggagaacugauaagggca....................	10	1	Mm2
....uggacggagaacugauaagggGa....................	3	1	Mm2
....uggacggagaacugauaUgggca....................	121	1	Mm2
....uggacgNagaacugauaagggca....................	1	1	Mm2
....uggacggagaacugauaagggcaA...................	92	1	Mm2
....uggaAggagaacugauaagggcau...................	1	1	Mm2
....uggacggagaacugauUagggcau...................	3	1	Mm2
....uggacggagaacugauaagggUau...................	175	1	Mm2
....uggacggagaacugauaGgggcau...................	23	1	Mm2
....uggacggagaacugauaCgggcau...................	8	1	Mm2
....uggacggagaacugauaaggCcau...................	1	1	Mm2
....uggacggagaacugauaagggcGu...................	27	1	Mm2
....uggacggagaacugauaUgggcau...................	11	1	Mm2
....Cggacggagaacugauaagggcau...................	1	1	Mm2
....Nggacggagaacugauaagggcau...................	1	1	Mm2
....uggacggaUaacugauaagggcau...................	1	1	Mm2
....uggacggagaaUugauaagggcau...................	1	1	Mm2
....uggacggagaUcugauaagggcau...................	187	1	Mm2
....uggacggagaacugauCagggcau...................	2	1	Mm2
....ugCacggagaacugauaagggcau...................	1	1	Mm2
....uggacggagaCcugauaagggcau...................	2	1	Mm2
....uggCcggagaacugauaagggcau...................	1	1	Mm2
....uggacggagaGcugauaagggcau...................	7	1	Mm2
....uggacggagaacugauaagggcCu...................	20	1	Mm2
....uggacggagGacugauaagggcau...................	1	1	Mm2
....uggacggagaacugauaagUgcau...................	2	1	Mm2
....uggacggagaacugauaagggcau...................	2426	0	Mm2
....uggacggagaacugauaagggcaC...................	27	1	Mm2
....uggacggagaacugauaagggAau...................	449	1	Mm2
....uggacggagaacugauaagAgcau...................	1	1	Mm2
....uggacggagaacugauGagggcau...................	4	1	Mm2

Star / Mature			
cauuugugacaucagaaguaagucuguuucgugu**ccucuucacuuuuccauccugu**cuguacucgagaac**uggacggagaacugauaagggc**auagggcagaccacaucaaca			
....uggacggagaacugauaagggGau...................	1	1	Mm2
....uggacggUgaacugauaagggcau...................	2	1	Mm2
....uggacggagCacugauaagggcau...................	1	1	Mm2
....uggacggagaacugauaagggcaG...................	52	1	Mm2
....uAgacggagaacugauaagggcau...................	1	1	Mm2
....uggacggagaacugauaaUggcau...................	1	1	Mm2
....uggacggagaacugauaagggcauC..................	5	1	Mm2
....uggacggagaacugauaagggcUua..................	8	1	Mm2
....uggacggagaacugauaagggcaAa..................	8	1	Mm2
....uggacggagaacugauaagggcauU..................	313	1	Mm2
....uggacggagaacugauaagggcaua..................	7	0	Mm2
....uggacggagaacugauaagggcauG..................	1	1	Mm2
....uggacggagaacugauaagggcauUg.................	1	1	Mm2
....uggacggagaacugauaagggcauaU.................	9	1	Mm2
....ggacggagaacugauaag........................	17	0	Mm2
....ggacggagaaAugauaag........................	1	1	Mm2
....ggacggagaacugauaaA........................	8	1	Mm2
....ggacggagaacugauaaU........................	1	1	Mm2
....ggacggagaacugauaagU.......................	1	1	Mm2
....ggacggagaGcugauaagg.......................	1	1	Mm2
....ggacggagaacugauaagA.......................	5	1	Mm2
....ggacggagaacugauaagg.......................	47	0	Mm2
....ggacggagaacuUauaagg.......................	1	1	Mm2
....ggacggagaacugauaUgg.......................	1	1	Mm2
....ggGcggagaacugauaagg.......................	1	1	Mm2
....Ugacggagaacugauaagg.......................	2	1	Mm2
....ggacggaAaacugauaaggg......................	1	1	Mm2
....ggacggagaaAugauaaggg......................	2	1	Mm2
....ggacggagaacugauaaggA......................	11	1	Mm2
....ggacggagUacugauaaggg......................	1	1	Mm2
....ggacggagaacugauaaggC......................	2	1	Mm2
....ggacggagaacugaCaaggg......................	1	1	Mm2
....ggacggagaacugauaaggg......................	48	0	Mm2
....Ugacggagaacugauaaggg......................	1	1	Mm2
....ggacggagaacugauaaggU......................	3	1	Mm2
....ggacggagaGcugauaagggc.....................	1	1	Mm2
....ggacggaAaacugauaagggc.....................	1	1	Mm2
....ggacggagaacugauaaUggc.....................	1	1	Mm2
....ggacggagaaGugauaagggc.....................	4	1	Mm2
....Ugacggagaacugauaagggc.....................	20	1	Mm2
....ggacggagaacugauaaggUc.....................	3	1	Mm2
....ggacggagaCcugauaagggc.....................	1	1	Mm2
....ggacggagaacugauaaggCc.....................	5	1	Mm2
....ggacggagaacugauaagUgc.....................	1	1	Mm2
....ggacggagaacugauaaggAc.....................	2	1	Mm2
....ggacggagaacugauaagAgc.....................	1	1	Mm2
....ggacggagCacugauaagggc.....................	8	1	Mm2
....ggacggagUacugauaagggc.....................	1	1	Mm2
....ggGcggagaacugauaagggc.....................	2	1	Mm2
....ggacggagaacugauaUgggc.....................	2	1	Mm2
....ggacCgagaacugauaagggc.....................	1	1	Mm2
....ggacggagaacugauaGgggc.....................	12	1	Mm2
....gAacggagaacugauaagggc.....................	2	1	Mm2
....ggaAggagaacugauaagggc.....................	1	1	Mm2
....ggacggGgaacugauaagggc.....................	1	1	Mm2
....ggacgUagaacugauaagggc.....................	1	1	Mm2
....ggacggagaacugauaagggU.....................	9	1	Mm2
....ggaGggagaacugauaagggc.....................	1	1	Mm2
....ggacggagaacugauaCgggc.....................	4	1	Mm2
....ggacggagaaAugauaagggc.....................	2	1	Mm2
....ggacggagaacugauaagggA.....................	26	1	Mm2
....ggacgAagaacugauaagggc.....................	1	1	Mm2
....ggacggagaacuUauaagggc.....................	2	1	Mm2
....ggacggagaacugauaagggc.....................	887	0	Mm2
....ggacggagaaUugauaagggc.....................	3	1	Mm2
....ggacggagaacugauaagCgc.....................	2	1	Mm2
....ggacggagaacugauCagggc.....................	3	1	Mm2
....ggUcggagaacugauaagggc.....................	2	1	Mm2
....Ugacggagaacugauaagggca....................	2	1	Mm2
....ggacggagaacugauaagggcC....................	1	1	Mm2

Star Mature

```
cauuugugacaucagaaguaagucuguuucguguccucuucacuuuuccauccuguc uguacucgagaacuggacggagaacugauaagggcauagggcagaccacaucaaca
```

```
cauuugugacaucagaaguaagucuguuucguguccucuucacuuuuccauccugucuguacucgagaacuggacggagaacugauaagggcauagggcagaccacaucaaca
.......................................................................ggacggagaacugauaagggca....................  79  0  Mm2
.......................................................................ggacggagaacugauaagAgca....................  1  1  Mm2
.......................................................................ggacggagaacugauaagggcG....................  3  1  Mm2
.......................................................................ggacggagaaAugauaagggca....................  1  1  Mm2
.......................................................................ggacggagaUcugauaagggca....................  4  1  Mm2
.......................................................................ggacggagaacugauaagggAau...................  2  1  Mm2
.......................................................................ggaAggagaacugauaagggcau...................  1  1  Mm2
.......................................................................ggacggagaaUugauaagggcau...................  1  1  Mm2
.......................................................................ggacggagaacugauaagggcau...................  3  0  Mm2
........................................................................gacggagaacugauaagggc.....................  1  0  Mm2
........................................................................gacggagaacugauaagggcauU..................  1  1  Mm2
.........................................................................acggagaacugauaaggg......................  1  0  Mm2
.........................................................................acggagaacugauaagggc.....................  3  0  Mm2
.........................................................................acUgagaacugauaagggc.....................  1  1  Mm2
..........................................................................Uggagaacugauaagggc.....................  3  1  Mm2
..........................................................................cggagaacugauaagggc.....................  1  0  Mm2
...........................................................................ggagaacugauaagggca....................  1  0  Mm2
```
